# Genetic analysis implicates *APOE, SNCA* and suggests lysosomal dysfunction in the etiology of dementia with Lewy bodies

**DOI:** 10.1093/hmg/ddu334

**Published:** 2014-06-27

**Authors:** Jose Bras, Rita Guerreiro, Lee Darwent, Laura Parkkinen, Olaf Ansorge, Valentina Escott-Price, Dena G. Hernandez, Michael A. Nalls, Lorraine N. Clark, Lawrence S. Honig, Karen Marder, Wiesje M. Van Der Flier, Afina Lemstra, Philip Scheltens, Ekaterina Rogaeva, Peter St George-Hyslop, Elisabet Londos, Henrik Zetterberg, Sara Ortega-Cubero, Pau Pastor, Tanis J. Ferman, Neill R. Graff-Radford, Owen A. Ross, Imelda Barber, Anne Braae, Kristelle Brown, Kevin Morgan, Walter Maetzler, Daniela Berg, Claire Troakes, Safa Al-Sarraj, Tammaryn Lashley, Yaroslau Compta, Tamas Revesz, Andrew Lees, Nigel Cairns, Glenda M. Halliday, David Mann, Stuart Pickering-Brown, Dennis W. Dickson, Andrew Singleton, John Hardy

**Affiliations:** 1 Department of Molecular Neuroscience,; 2 Queen Square Brain Bank, Department of Molecular Neuroscience, and; 3 Reta Lila Weston Research Laboratories, Department of Molecular Neuroscience, UCL Institute of Neurology, London WC1N 3BG, UK,; 4 Nuffield Department of Clinical Neurosciences, Oxford Parkinson's Disease Centre, University of Oxford, Oxford, UK,; 5 MRC Centre for Neuropsychiatric Genetics and Genomics, School of Medicine, Cardiff University, Cardiff, UK,; 6 Laboratory of Neurogenetics, National Institutes on Aging, NIH, Bethesda, USA,; 7 Taub Institute for Alzheimer Disease and the Aging Brain,; 8 Department of Pathology and Cell Biology, and; 9 Department of Neurology, Columbia University, New York, NY, USA,; 10 Department of Neurology and Alzheimer Center, Neuroscience Campus Amsterdam, VU University Medical Center, Amsterdam, the Netherlands,; 11 Department of Medicine, Tanz Centre for Research in Neurodegenerative Diseases, University of Toronto, Toronto, Ontario, Canada,; 12 Cambridge Institute for Medical Research, and Cambridge National Institute of Health Research Biomedical Research Unit in Dementia, University of Cambridge, Cambridge CB2 0XY, UK,; 13 Clinical Memory Research Unit, Institute Clinical Sciences Malmö, Lund University, Sweden,; 14 Clinical Neurochemistry Laboratory, Department of Psychiatry and Neurochemistry, Institute of Neuroscience and Physiology, the Sahlgrenska Academy at the University of Gothenburg, Mölndal, Sweden,; 15 Neurogenetics Laboratory, Division of Neurosciences, Center for Applied Medical Research, University of Navarra, Pamplona, Spain,; 16 Department of Neurology, Clínica Universidad de Navarra, University of Navarra School of Medicine, Pamplona, Spain,; 17 CIBERNED, Centro de Investigación Biomédica en Red de Enfermedades Neurodegenerativas, Instituto de Salud Carlos III, Madrid, Spain,; 18 Department of Psychiatry,; 19 Department of Psychology,; 20 Department of Neurology and; 21 Department of Neuroscience, Mayo Clinic, Jacksonville, FL, USA,; 22 Translation Cell Sciences - Human Genetics, School of Life Sciences, Queens Medical Centre, University of Nottingham, Nottingham, UK,; 23 Hertie Institute for Clinical Brain Research, Department of Neurodegeneration, Center of Neurology, University of Tuebingen, and DZNE, German Center for Neurodegenerative Diseases, Tuebingen, Germany,; 24 MRC London Neurodegenerative Diseases Brain Bank, Department of Clinical Neuroscience, King's College London, Institute of Psychiatry, London, UK,; 25 Knight Alzheimer's Disease Research Center and; 26 Department of Neurology, Washington University School of Medicine, Saint Louis, MO, USA,; 27 Neuroscience Research Australia, Sydney, Australia,; 28 School of Medical Sciences, Faculty of Medicine, University of New South Wales, Sydney, Australia,; 29 Institute of Brain, Behaviour and Mental Health, Faculty of Medical and Human Sciences, University of Manchester, Manchester, UK and; 30 Parkinson's disease and Movement Disorders Unit, Neurology Service, IDIBAPS, CIBERNED, Hospital Clínic, Barcelona, Catalonia, Spain

## Abstract

Clinical and neuropathological similarities between dementia with Lewy bodies (DLB), Parkinson's and Alzheimer's diseases (PD and AD, respectively) suggest that these disorders may share etiology. To test this hypothesis, we have performed an association study of 54 genomic regions, previously implicated in PD or AD, in a large cohort of DLB cases and controls. The cohort comprised 788 DLB cases and 2624 controls. To minimize the issue of potential misdiagnosis, we have also performed the analysis including only neuropathologically proven DLB cases (667 cases). The results show that the *APOE* is a strong genetic risk factor for DLB, confirming previous findings, and that the *SNCA* and *SCARB2* loci are also associated after a study-wise Bonferroni correction, although these have a different association profile than the associations reported for the same loci in PD. We have previously shown that the p.N370S variant in *GBA* is associated with DLB, which, together with the findings at the *SCARB2* locus, suggests a role for lysosomal dysfunction in this disease. These results indicate that DLB has a unique genetic risk profile when compared with the two most common neurodegenerative diseases and that the lysosome may play an important role in the etiology of this disorder. We make all these data available.

## INTRODUCTION

Dementia with Lewy bodies (DLB) is commonly regarded as the second largest neuropathological subgroup of neurodegenerative dementing disorders, preceded only by Alzheimer's disease (AD) (1). DLB and Parkinson's disease with dementia (PDD) are both progressive neurodegenerative disorders and together they account for 15–20% of all people with dementia (2). The clinical characteristics of DLB include cognitive decline, parkinsonian signs, fluctuations in cognition and attention, and visual hallucinations (3). Neuropathological diagnosis of DLB is based on the widespread finding of Lewy bodies (LB) throughout the nervous system, both CNS and PNS. The overlap in clinical presentation between DLB and both AD and PDD, often causes difficulty in diagnosing DLB (4). The incidence of motor symptoms in patients with AD is much higher than expected in age-matched controls (5). Dementia is frequent in late phases of PD and present in up to 80% of patients with 20-year duration of motor symptoms (6). If dementia occurs before or within the first year from the start of the motor symptoms, the patients are diagnosed with DLB (7). Neuropathological examination is not always conclusive for a diagnosis of DLB. A proportion of DLB neuropathologically diagnosed patients present concomitant AD pathology in various degrees (8), and LBs are the hallmark of PD with and without dementia, thus increasing the problems with diagnosis. On the other hand, the fact that these three disorders have overlaps in both clinical presentations and in neuropathological features suggests that they may also share etiology.

Over the last few years, several advances in the understanding of the genetics underlying neurological diseases have been made. Several loci, and sometimes genes, have been conclusively shown to modulate risk for a given phenotype. This was the case for both AD (9–12) and PD (13,14) each with several publications replicating initial findings. The same however has not been true for DLB and the disease etiology still remains mostly elusive, with only a small number of reports suggesting the involvement of *APOE* and *GBA* as risk factors for the development of the disease (15–18). DLB is generally considered a sporadic disorder, but a few cases of familial aggregation have been described. However, even in such cases, the identification of the underlying causal gene has not been a successful endeavor (19).

Given the similarities between DLB and both PD and AD, at the clinical as well as at the neuropathological level, we have tested the most recently associated loci for AD and PD, in a large multi-national cohort of DLB cases and controls.

## RESULTS

The final analysis data set comprised of 788 cases and 2624 controls, with genotypes at 6078 loci. These loci were selected from the NeuroX array, based on 500 kb flanking regions of each top association hit from the latest PD and AD genome-wide association studies (GWAS). Supplementary Material, Table S1 details these regions, as well as the level of coverage in NeuroX for each of the reported top hits. The majority of top hits are well covered in our study with markers showing *r*-squared values >0.6.

**Figure 1.**
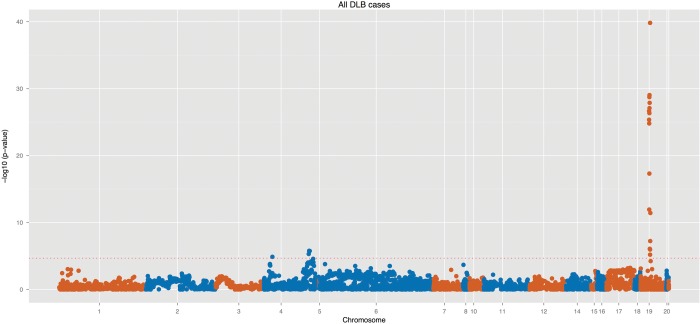
Genomic overview of the association at 54 genomic regions in a cohort of 788 DLB cases and 2624 controls. These loci correspond to 500 kb flanking regions of each top association hit from the latest PD and AD GWAS (32 regions from PD and 22 from AD GWAS). The dotted horizontal red line represents our study-wise Bonferroni correction threshold of 3.7 × 10^−5^.

Three regions showed evidence of strong association with DLB: the *APOE*, *SNCA* and the *SCARB2* loci (Table 1). The first is a widely known genetic risk factor for AD and has also been reported to be a risk factor for DLB in smaller size studies, the other two loci are known to impart risk for PD, albeit with very disparate effect sizes. Figure 1 shows a genomic overview of the association and Supplementary Material, Table S2 compares the most significant results from this study with the previously reported hits at each one of the regions.

**Table 1.** Most significantly associated hits from the present study and comparison with previously reported hits from PD and AD associations

Study/named region	Reported top hit	Reported *P*-value	Reported OR	Best DLB hit	*P*-value	OR (95% CI)
AD_IGAP2013:APOE	NA	NA	NA	exm-rs769449	1.52E−40	2.786 (2.397–3.239)
PD_MegaMeta:SNCA	rs356182	4.16x-73	0.76	NeuroX_rs894280	1.67E−06	0.754 (0.6725–0.8468)
PD_MegaMeta:FAM47E/SCARB2	rs6812193	2.95x-11	0.907	NeuroX_rs6825004	1.35E−05	0.749 (0.658–0.854)

**Figure 2.**
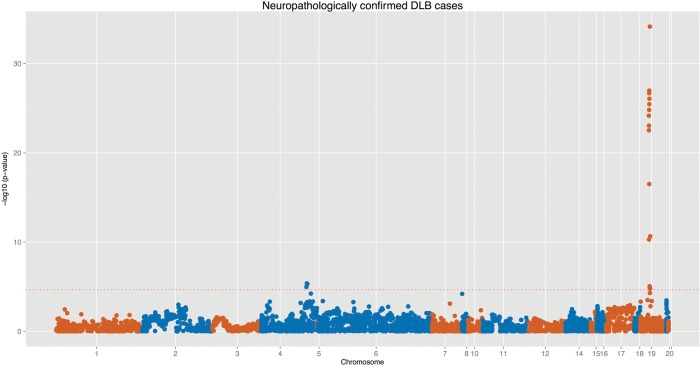
Genomic overview of the association using only neuropathologically confirmed DLB cases at the previously reported association hits for PD and AD. These loci correspond to 500 kb flanking regions of each top association hit from the latest PD and AD GWAS (32 regions from PD and 22 from AD GWAS). The dotted horizontal red line represents our study-wise Bonferroni correction threshold of 3.7 × 10^−5^.

Because the diagnosis of DLB is frequently a complicated task, given the overlaps with PD and AD, this may lead to misdiagnosis of the subjects. Since the combination of a clinical and a neuropathological workup is more accurate than just a clinical workup, we performed the association using only neuropathologically proven cases. This led to the inclusion of 667 cases and 2624 controls. When performing logistic regression in this subcohort both *APOE* and *SNCA* remained significant, even after multiple test correction. *SCARB2,* in turn, shows a suggestive level of association (*P* = 0.0004), consistent with a similar effect size from a smaller number of samples (Fig. 2). Interestingly, the *MAPT* locus, one of the strongest hits in PD, showed no evidence of association with DLB, using any of the approaches.

A single marker in *FGF20* becomes nearly statistically significant (*P* = 6.47 × 10^−05^) when using only pathologically confirmed DLB cases. *FGF20* is a fibroblast growth factor expressed throughout the brain and it was recently shown to be associated with PD (14,20).

**Figure 3.**
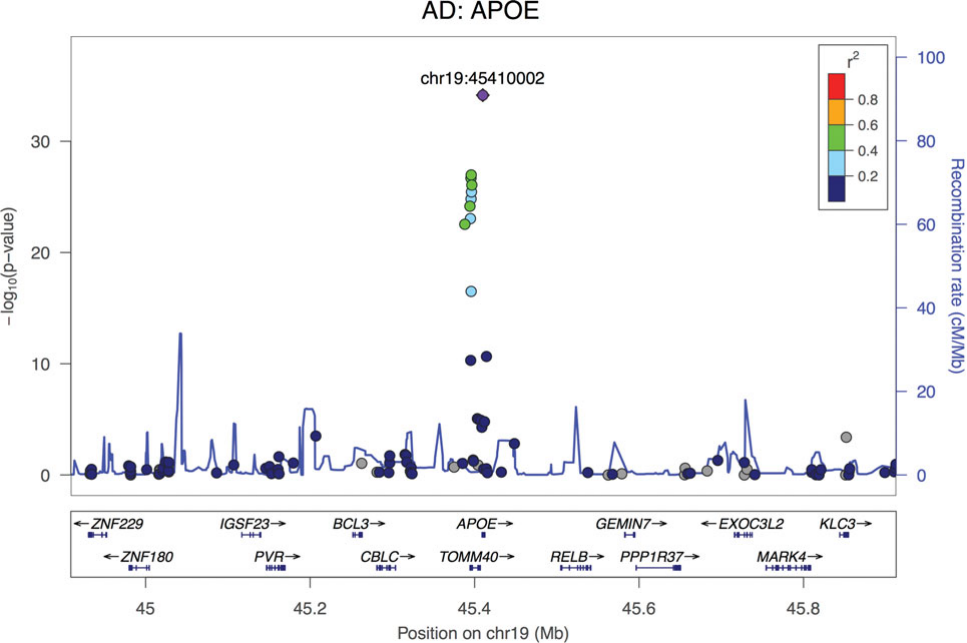
Regional association plot of the *APOE* genomic locus (the top hit from GWAS in AD) in a cohort of 788 DLB cases and 2624 controls. Coloring is based on linkage disequilibrium (LD) with the most associated SNP.

**Figure 4.**
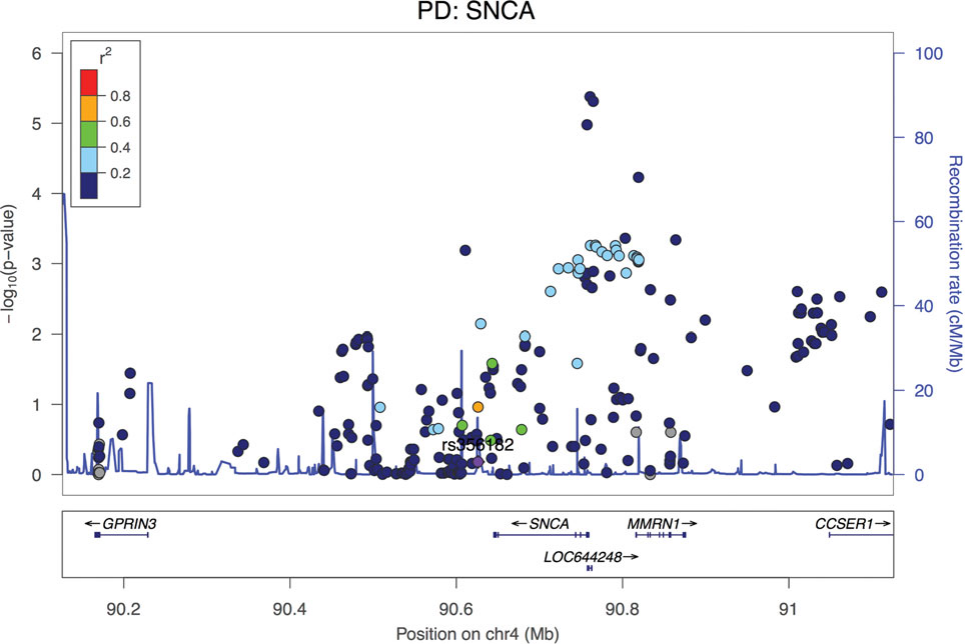
Regional association plot at the *SNCA* locus (the top hit from GWAS in PD) in a cohort of 788 DLB cases and 2624 controls. Interestingly, the top hit at this locus for PD is not significant in our DLB study (in purple) and the association seems to be 5′ of the gene. Coloring represents LD with the top reported hit for PD.

**Figure 5.**
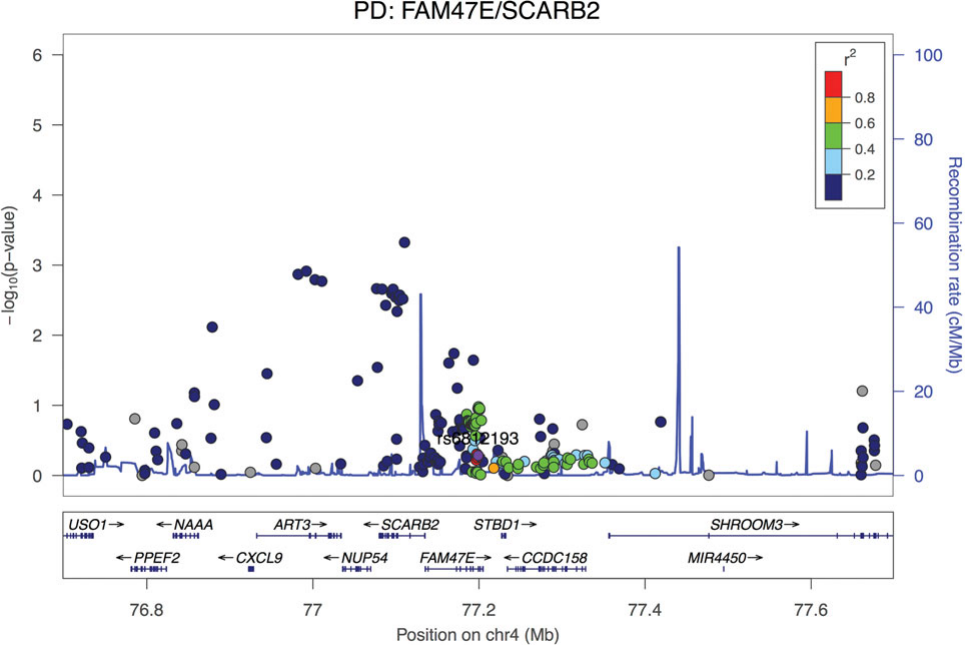
Regional association plot at the *SCARB2* locus (one of the top hits from GWAS in PD) in a cohort of 788 DLB cases and 2624 controls. The top hit in DLB is not in high LD with the reported hit for PD (in purple). Coloring represents LD with the top reported hit for PD.

Supplementary Material, Table S3 details the top statistically significant results at the loci and Figures 3–5 show the regional association at the three most significant hits. None of the remaining regions previously shown to be associated with PD or AD by GWAS showed sign of strong association with DLB (Supplementary Material, Figures); including *LRRK2*, which is known to have both disease-causing mutations as well as risk-conferring variants for PD.

Although not a GWAS hit, the *GBA* gene is a known strong risk factor for PD and the p.N370S variant has been identified as the most common variant to impart risk for this disease in Caucasian populations (21). Our results do not strongly confirm the association of this variant with DLB (*P* = 0.005). Though not a Gaucher's disease-causing mutation, the p.E326K variant in *GBA* has recently been shown to also increase risk for PD and DLB (22,23). Again, our data do not strongly support the involvement of the p.E326K variant in DLB (*P* = 0.08).

## DISCUSSION

The main aim of this study was to determine whether genetic loci associated with PD or AD are involved in DLB. To that end, we used a large DLB cohort, comprised mostly of neuropathologically diagnosed samples, and genotyped markers in previously reported loci.

A study of this size does not have sufficient statistical power to identify novel associations of small to intermediate effect size. However, we estimate that we have excellent power to detect ORs of >10 for rare variants (<0.01 frequency in the general population) as is the case of APOE in AD, and over 80% power to detect variants with OR of 3 and a frequency in the general population >0.02. Despite these facts, this is the largest ever association study reported in DLB and therefore it is the most powerful study published so far.

Three loci were unequivocally associated with DLB in this cohort: the *APOE*, *SNCA* and *SCARB2* regions.

The association at *APOE* is not surprising, as previous studies had shown this association (24), which is driven by the epsilon4 allele. As the strongest genetic risk factor for AD, a disease characterized by beta-amyloid deposition and not LB formation, it is interesting that the same *APOE* risk factor involved in AD is also the strongest genetic risk factor for DLB. Since DLB is not only characterized by LBs but also cerebral β-amyloidosis (25), it is likely that the association of DLB with the epsilon4 allele of *APOE* is driven by the Aβ pathology-promoting effect of this particular variant. However, Aβ-independent mechanisms cannot be excluded. While this result could be viewed as a product of the inclusion of AD cases misdiagnosed as DLB in our cohort, a few factors argue against that hypothesis. Firstly, the association remains highly significant when we restrict the analysis to neuropathologically diagnosed DLB cases; and second, if a contamination with AD cases were to have occurred, we would expect to see associations with the other reported GWAS hits in AD.

**Figure 6.**
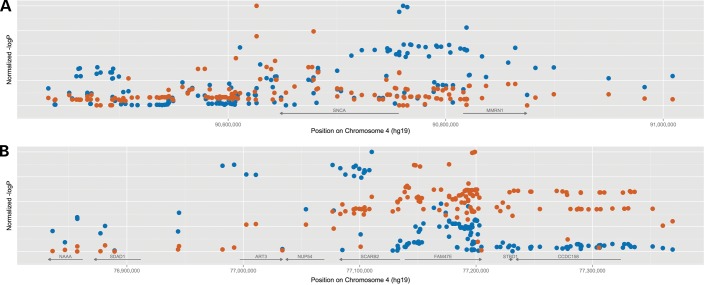
Comparison of the regional association at the *SNCA* and *SCARB2* loci between DLB and PD. Red symbols represent the PD association (restricted to the top hits per region), while the blue ones represent DLB. *P*-values were normalized for each region/study to allow for a better comparison of regional association.

An association at the *SNCA* locus is also not entirely surprising given that the product of that gene, the protein alpha-synuclein, is the major protein component of LBs. It is, however, surprising that the association at this locus appears to be distinct from the one reported in PD. In that disease, *SNCA* is the strongest common genetic risk factor closely followed by the *MAPT* locus. However, when we compare the association region between PD and DLB, it is clear that the haplotype conferring risk is different in these two diseases, with PD having an association 3′ to gene and DLB appearing to occur 5′ of the gene (Fig. 6A). Although it is not clear at this stage what are the implications of this disparity, it is possible that it has an influence on the distribution of the LBs in the brain tissue, given that these are generally localized to brainstem in PD and have a more widespread distribution in all DLB cases (26), presumably through differential expression of the gene. The association is maintained when only neuropathologically proven DLB cases are included in the analysis. This shows that this novel association is DLB specific and not due to misdiagnosis of PD with dementia cases as DLB.

The third association is also on chromosome 4 and also a PD-reported locus. The *SCARB2* gene encodes a lysosomal protein that is associated with PD, a disease where other lysosomal genes have been implicated. However, it is not one of the strongest genetic risk factors for PD and only after large meta-analysis of data, does it become statistically significant (14). It is thus interesting that it is the third strongest risk factor for DLB in our study. However, and similarly to the *SNCA* locus, the association profile of *SCARB2* is also different between PD and DLB, with the top hit in PD not being significant in DLB (Fig. 6B). Strengthening the hypothesis of the involvement of the lysosome in DLB is the fact that the p.N370S variant in *GBA* was shown to be associated with DLB. However, as genes in the array are not randomly drawn from the genome, it is impossible to formally assess whether there is an enrichment of a lysosomal dysfunction signal in these data and this, therefore, requires further investigation.

It is notable that *GBA* shows an association with DLB and that we have not seen an association between DLB and *LRRK2* variants. In agreement with this, others have shown that *GBA* encoded PD is more prone to developing dementia than *LRRK2*-encoded disease (27,28).

We have previously shown that AD and PD do not share association regions between them (29). The data we present in this paper offers a possible explanation for this lack of overlap. Diagnoses of PD and AD are designed to separate these entities. DLB is the diagnosis made in those who have elements of both pathologies, and in agreement with this, they share risk loci with AD and PD. However, we also show that the associations with DLB are different from those in PD, in that they implicate different regions of the pathogenic loci, presumably relating to different gene expression control elements.

We have also tested if there is an overrepresentation of previously associated variants for PD and AD as associated with DLB in our cohort. We were able to either directly assay or use a proxy (*r*^2^ > 0.8) for 37 of the 54 previously associated markers with PD or AD. Of these, 33 showed a *P*-value <0.05. Using a binomial test, we calculated that there is a significant overrepresentation of significantly associated variants (*P* < 2.2 × 10^−16^).

Here, we have performed a large association study in DLB to assess if loci previously implicated in AD and PD also play a role in this disease. We identified three genomic regions that are involved in DLB. We also show that the association at the *APOE* locus is similar to the one that occurs in AD, in accordance with previous studies, and that the *SNCA* and *SCARB2* associations are different to the ones reported in PD and possibly PD with dementia. The possible involvement of *SCARB2* and the previously identified results at *GBA* seem to implicate the lysosome in the etiology of this prevalent disorder. Overall, these results suggest that the etiology of DLB is influenced by some of the same genetic risk factors for AD and PD, but that these loci may act in subtly different manners. Clearly, large studies on this disorder are needed and to assist these we make our data available (Supplementary Material, Table S4).

## MATERIALS AND METHODS

DLB cases were selected based on clinical and/or neuropathological diagnosis criteria. In both cases, diagnosis was made according to the most recently published criteria (7). A total of 121 clinically and 667 neuropathologically diagnosed samples, originating from 12 study centers, were included in the study (Table 2). A total of 2624 samples from healthy individuals were included as a control group (M:F ratio = 0.87). All samples are from European or North American descent.

**Table 2.** DLB cohort description

Cohort	*n*	Neuropathological diagnosis	M:F ratio
Swe/H	17	Yes	3.25
Aus/G	63	Yes	2.25
UK/Man	94	Yes	2.65
UK/Not	40	Yes	1.65
UK/Oxf	50	Yes	1.21
US/AG	143	Yes	0.76
UK/QS	31	Yes	3.63
US/Mayo	152	Yes	1.38
US/LC	77	Yes	2.2
Can/E	27	No	0.93
SP/PP	30	No	1.12
Ams/W	64	No	2.9
Total	788		

*n*, number of samples included; M:F ratio, male to female ratio.

All samples were genotyped using the NeuroX array (Illumina, Inc.), which is a publicly available custom array designed specifically for the study of neurological diseases. In addition to the base content on Illumina's Exome BeadChip, the NeuroX contains markers specific for neurological diseases, in the form of known and unpublished GWAS hits, rare, high effect size variants as well as rare variants identified through exome-sequencing studies of neurodegenerative diseases.

After DNA preparation and genotyping according to standard Illumina Infinium methods, variants were clustered using Illumina's GenomeStudio. The base content of the array was clustered according to the published cluster file from the CHARGE consortium (30), while the custom content, comprising ∼25 000 variants, was manually assessed/clustered whenever GenTrain scores were <0.9. Markers that were impossible to cluster properly were excluded. The entire array content was used for QC methods, which included removing markers with missingness >10%, a minor allele frequency of <1%, those deviating from the Hardy–Weinberg equilibrium (*P* < 1 × 10^−4^) and finally also excluding samples with >10% of missing genotypes. Following these QC steps, the total cohort comprised of 788 cases and 2624 controls genotyped at 79 152 markers.

Since the aim of this study was to verify if DLB shares genetic risk with PD and AD, we extracted all variants within 500 kb of reported association hits for these two diseases. For AD, we used the data recently published (11), while for PD, because the work is currently under submission, we used unpublished data. For each study, we selected the top hit in each associated genomic region surpassing genome-wide Bonferroni correction (relevant to each separate study) and identified a region of interest of 1 Mb in total. A total of 7275 markers were extracted from the entire content, with 1325 being independent variants (pairwise *r*^2^ <0.5).

To generate covariates for logistic regression models, multidimensional scaling was used to quantify genetic distances between members of the case–control cohort. Association testing was performed using the logistic regression function in PLINK (31) using gender and the first 10 principal components as covariates to adjust for possible population substructure.

We used a conservative Bonferroni significance threshold of *P* = 3.7 × 10^−5^ for all associations tested. This threshold was based on the total number of independent tests performed at the previously reported hit regions (0.05/1325).

## SUPPLEMENTARY MATERIAL

Supplementary Material is available at *HMG* online.

*Conflict of Interest statement*. None declared.

## FUNDING

This work was supported in part by a Parkinson's UK Innovation Award (K-1204) and by the Wellcome Trust/MRC Joint Call in Neurodegeneration award (WT089698) to the UK Parkinson's Disease Consortium whose members are from the UCL Institute of Neurology, the University of Sheffield, and the MRC Protein Phosphorylation Unit at the University of Dundee and by an anonymous Foundation. R.G. is supported by an Alzheimer's Research UK Travelling Fellowship. The authors would like to acknowledge Elena Lorenzo for her technical assistance. This study was supported in part by grants from the Spanish Ministry of Science and Innovation SAF2006-10126 (2006–2009) and SAF2010-22329-C02-01 (2011–2013) to P.P. and by the UTE project FIMA to P.P. The sample collection and database of the Amsterdam Dementia Cohort was funded by Stichting Dioraphte and Stichting VUMC fonds. G.M.H. is a Senior Principal Research Fellow of the National Health and Medical Research Council of Australia. For the neuropathologically confirmed samples from Australia, brain tissue was received from the Sydney Brain Bank, which is supported by Neuroscience Research Australia, the University of New South Wales and the National Health and Medical Research Council of Australia. This study was also partially funded by the Wellcome Trust, Medical Research Council, Canadian Institutes of Health Research, Ontario Research Fund. The Nottingham Genetics Group is supported by ARUK and The Big Lottery Fund. The effort from Columbia University was supported by the Taub Institute, the Panasci Fund, the Parkinson's Disease Foundation, and NIH grants NS060113 (L. Clark), P50AG008702 (P.I. Scott Small), P50NS038370 (P.I. R. Burke), and UL1TR000040 (P.I. H. Ginsberg). O.A.R. is supported by the Michael J. Fox Foundation, NINDS R01# NS078086. The Mayo Clinic Jacksonville is a Morris K. Udall Parkinson's Disease Research Center of Excellence (NINDS P50 #NS072187) and is supported by the Mangurian Foundation for Lewy body research. This work has received support from The Queen Square Brain Bank at the UCL Institute of Neurology. Some of the tissue samples studied were provided by the MRC London Neurodegenerative Diseases Brain Bank and the Brains for Dementia Research project (funded by Alzheimer's Society and ARUK). This research was supported in part by both the NIHR UCLH Biomedical Research Centre and the Queen Square Dementia Biomedical Research Unit. This work was supported in part by the Intramural Research Program of the National Institute on Aging, National Institutes of Health, Department of Health and Human Services; project AG000951-12. Funding to pay the Open Access publication charges for this article was provided by the Wellcome Trust and the Medical Research Council.

## Supplementary Material

Supplementary Data

## References

[1] Barber R., Panikkar A., McKeith I.G. (2001). Dementia with Lewy bodies: diagnosis and management. Int. J. Geriatr. Psychiatry.

[2] Lippa C.F., Duda J.E., Grossman M., Hurtig H.I., Aarsland D., Boeve B.F., Brooks D.J., Dickson D.W., Dubois B., Emre M. (2007). DLB and PDD boundary issues: diagnosis, treatment, molecular pathology, and biomarkers. Neurology.

[3] McKeith I.G., Galasko D., Kosaka K., Perry E.K., Dickson D.W., Hansen L.A., Salmon D.P., Lowe J., Mirra S.S., Byrne E.J. (1996). Consensus guidelines for the clinical and pathologic diagnosis of dementia with Lewy bodies (DLB): report of the consortium on DLB international workshop. Neurology.

[4] McKeith I.G. (2002). Dementia with Lewy bodies. The British Journal of Psychiatry: the Journal of Mental Science.

[5] Scarmeas N., Hadjigeorgiou G.M., Papadimitriou A., Dubois B., Sarazin M., Brandt J., Albert M., Marder K., Bell K., Honig L.S. (2004). Motor signs during the course of Alzheimer disease. Neurology.

[6] Hely M.A., Reid W.G., Adena M.A., Halliday G.M., Morris J.G. (2008). The Sydney multicenter study of Parkinson's disease: the inevitability of dementia at 20 years. Move. Disord..

[7] McKeith I.G., Dickson D.W., Lowe J., Emre M., O'Brien J.T., Feldman H., Cummings J., Duda J.E., Lippa C., Perry E.K. (2005). Diagnosis and management of dementia with Lewy bodies: third report of the DLB Consortium. Neurology.

[8] Marui W., Iseki E., Kato M., Akatsu H., Kosaka K. (2004). Pathological entity of dementia with Lewy bodies and its differentiation from Alzheimer's disease. Acta Neuropathol..

[9] Lambert J.C., Heath S., Even G., Campion D., Sleegers K., Hiltunen M., Combarros O., Zelenika D., Bullido M.J., Tavernier B. (2009). Genome-wide association study identifies variants at CLU and CR1 associated with Alzheimer's disease. Nat. Genet..

[10] Harold D., Abraham R., Hollingworth P., Sims R., Gerrish A., Hamshere M.L., Pahwa J.S., Moskvina V., Dowzell K., Williams A. (2009). Genome-wide association study identifies variants at CLU and PICALM associated with Alzheimer's disease. Nat. Genet..

[11] Lambert J.C., Ibrahim-Verbaas C.A., Harold D., Naj A.C., Sims R., Bellenguez C., Jun G., Destefano A.L., Bis J.C., Beecham G.W. (2013). Meta-analysis of 74,046 individuals identifies 11 new susceptibility loci for Alzheimer's disease. Nat. Genet..

[12] Naj A.C., Jun G., Beecham G.W., Wang L.S., Vardarajan B.N., Buros J., Gallins P.J., Buxbaum J.D., Jarvik G.P., Crane P.K. (2011). Common variants at MS4A4/MS4A6E, CD2AP, CD33 and EPHA1 are associated with late-onset Alzheimer's disease. Nat. Genet..

[13] Simon-Sanchez J., Schulte C., Bras J.M., Sharma M., Gibbs J.R., Berg D., Paisan-Ruiz C., Lichtner P., Scholz S.W., Hernandez D.G. (2009). Genome-wide association study reveals genetic risk underlying Parkinson's disease. Nat. Genet..

[14] Nalls M.A., Plagnol V., Hernandez D.G., Sharma M., Sheerin U.M., Saad M., Simon-Sanchez J., Schulte C., Lesage S., Sveinbjornsdottir S. (2011). Imputation of sequence variants for identification of genetic risks for Parkinson's disease: a meta-analysis of genome-wide association studies. Lancet.

[15] Clark L.N., Kartsaklis L.A., Wolf Gilbert R., Dorado B., Ross B.M., Kisselev S., Verbitsky M., Mejia-Santana H., Cote L.J., Andrews H. (2009). Association of glucocerebrosidase mutations with dementia with Lewy bodies. Arch. Neurol..

[16] Kobayashi S., Tateno M., Park T.W., Utsumi K., Sohma H., Ito Y.M., Kokai Y., Saito T. (2011). Apolipoprotein E4 frequencies in a Japanese population with Alzheimer's disease and dementia with Lewy bodies. PLoS One.

[17] Pickering-Brown S.M., Mann D.M., Bourke J.P., Roberts D.A., Balderson D., Burns A., Byrne J., Owen F. (1994). Apolipoprotein E4 and Alzheimer's disease pathology in Lewy body disease and in other beta-amyloid-forming diseases. Lancet.

[18] Hardy J., Crook R., Prihar G., Roberts G., Raghavan R., Perry R. (1994). Senile dementia of the Lewy body type has an apolipoprotein E epsilon 4 allele frequency intermediate between controls and Alzheimer's disease. Neurosci. Lett..

[19] Bogaerts V., Engelborghs S., Kumar-Singh S., Goossens D., Pickut B., van der Zee J., Sleegers K., Peeters K., Martin J.J., Del-Favero J. (2007). A novel locus for dementia with Lewy bodies: a clinically and genetically heterogeneous disorder. Brain.

[20] van der Walt J.M., Noureddine M.A., Kittappa R., Hauser M.A., Scott W.K., McKay R., Zhang F., Stajich J.M., Fujiwara K., Scott B.L. (2004). Fibroblast growth factor 20 polymorphisms and haplotypes strongly influence risk of Parkinson disease. Am. J. Hum. Genet..

[21] Sidransky E., Nalls M.A., Aasly J.O., Aharon-Peretz J., Annesi G., Barbosa E.R., Bar-Shira A., Berg D., Bras J., Brice A. (2009). Multicenter analysis of glucocerebrosidase mutations in Parkinson's disease. N. Engl. J. Med..

[22] Duran R., Mencacci N.E., Angeli A.V., Shoai M., Deas E., Houlden H., Mehta A., Hughes D., Cox T.M., Deegan P. (2013). The glucocerobrosidase E326K variant predisposes to Parkinson's disease, but does not cause Gaucher's disease. Move. Disord..

[23] Nalls M.A., Duran R., Lopez G., Kurzawa-Akanbi M., McKeith I.G., Chinnery P.F., Morris C.M., Theuns J., Crosiers D., Cras P. (2013). A multicenter study of glucocerebrosidase mutations in dementia with Lewy bodies. JAMA Neurol..

[24] Tsuang D., Leverenz J.B., Lopez O.L., Hamilton R.L., Bennett D.A., Schneider J.A., Buchman A.S., Larson E.B., Crane P.K., Kaye J.A. (2013). APOE epsilon4 increases risk for dementia in pure synucleinopathies. JAMA Neurol..

[25] McKeith I.G., Ince P., Jaros E.B., Fairbairn A., Ballard C., Grace J., Morris C.M., Perry R.H. (1998). What are the relations between Lewy body disease and AD? *J*. Neural Transm. Suppl..

[26] Halliday G., Hely M., Reid W., Morris J. (2008). The progression of pathology in longitudinally followed patients with Parkinson's disease. Acta Neuropathol..

[27] Brice A. (2005). Genetics of Parkinson's disease: LRRK2 on the rise. Brain.

[28] Beavan M.S., Schapira A.H. (2013). Glucocerebrosidase mutations and the pathogenesis of Parkinson disease. Ann. Med..

[29] Moskvina V., Harold D., Russo G., Vedernikov A., Sharma M., Saad M., Holmans P., Bras J.M., Bettella F., Keller M.F. (2013). Analysis of genome-wide association studies of Alzheimer disease and of Parkinson disease to determine if these 2 diseases share a common genetic risk. JAMA Neurol.

[30] Grove M.L., Yu B., Cochran B.J., Haritunians T., Bis J.C., Taylor K.D., Hansen M., Borecki I.B., Cupples L.A., Fornage M. (2013). Best practices and joint calling of the HumanExome BeadChip: the CHARGE Consortium. PLoS One.

[31] Purcell S., Neale B., Todd-Brown K., Thomas L., Ferreira M.A., Bender D., Maller J., Sklar P., de Bakker P.I., Daly M.J. (2007). PLINK: a tool set for whole-genome association and population-based linkage analyses. Am. J. Hum. Genet..

