# Supplementary material for: Genetic analysis implicates APOE, SNCA and suggests lysosomal dysfunction in the etiology of dementia with Lewy bodies
Source: Hum Mol Genet. 2014 Jun 27;23(23):6139–46. doi: 10.1093/hmg/ddu334 (PMC4222357; doi:10.1093/hmg/ddu334)
Supplement: Supplementary Data [file supp_23_23_6139__index.html]

Genetic analysis implicates APOE, SNCA and suggests lysosomal dysfunction in the etiology of dementia with Lewy bodies — Genetic analysis implicates APOE, SNCA and suggests lysosomal dysfunction in the etiology of dementia with Lewy bodies — Supplementary Data 

# Genetic analysis implicates *APOE, SNCA* and suggests lysosomal dysfunction in the etiology of dementia with Lewy bodies

## Supplementary Data

Supplementary Data

**Files in this Data Supplement:**

- Supplementary Figures - pdf file
- Supplementary Table 1 to 3 - docx file
- Supplementary Table 4 - xlsx file
