# Supplementary material for: Genetic analysis implicates APOE, SNCA and suggests lysosomal dysfunction in the etiology of dementia with Lewy bodies
Source: Hum Mol Genet. 2014 Jun 27;23(23):6139–46. doi: 10.1093/hmg/ddu334 (PMC4222357; doi:10.1093/hmg/ddu334)
Supplement: Supplementary Data [file supp_ddu334_ddu334supp_figs.pdf]

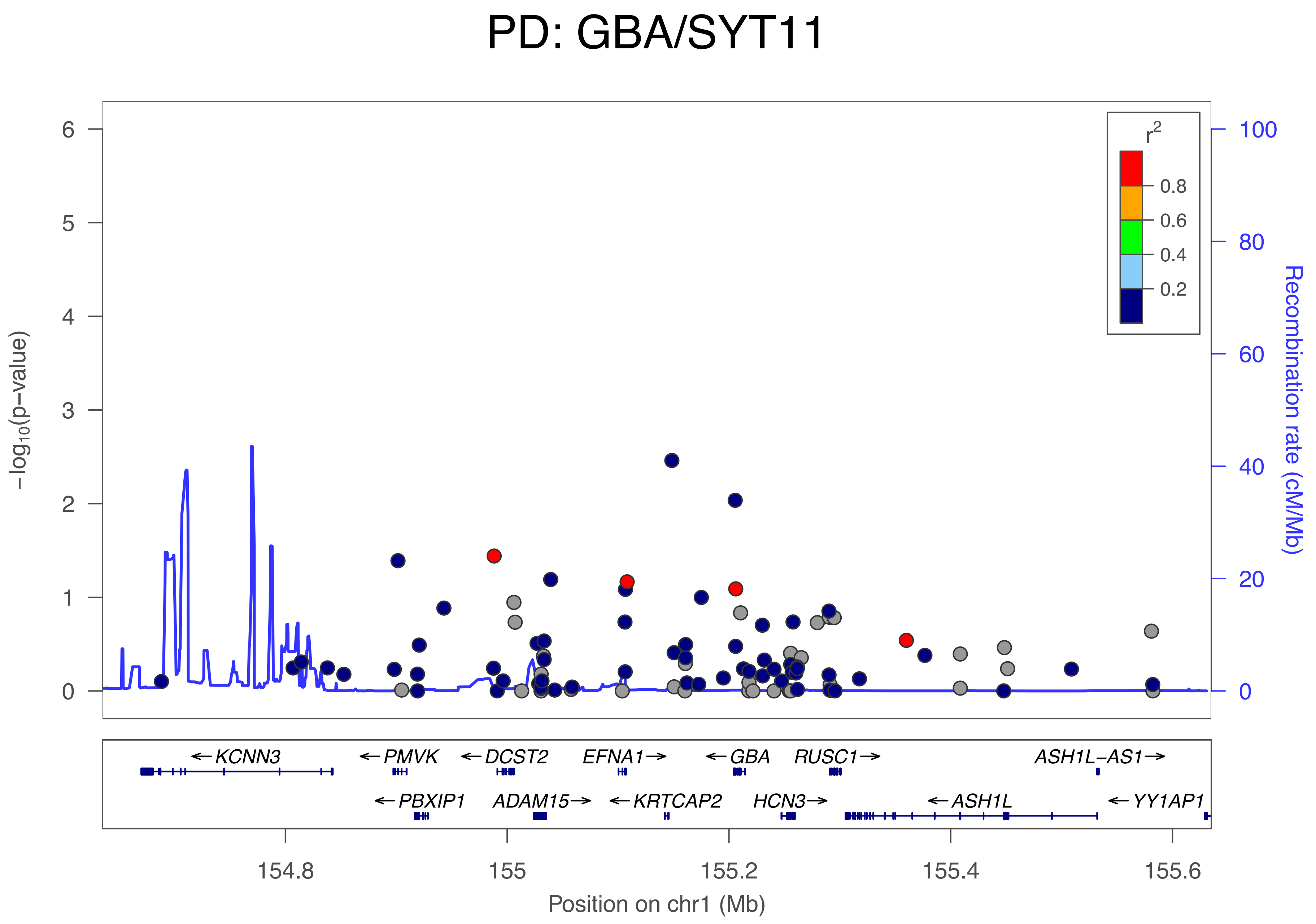
PD: GBA/SYT11
-log10(p-value)
Recombination rate (cM/Mb)
r2
0.8
0.6
0.4
0.2
KCNN3
PMVK
DCST2
EFNA1
GBA
RUSC1
ASH1L-AS1
PBXIP1
ADAM15
KRTCAP2
HCN3
ASH1L
YY1AP1
154.8
155
155.2
155.4
155.6
Position on chr1 (Mb)

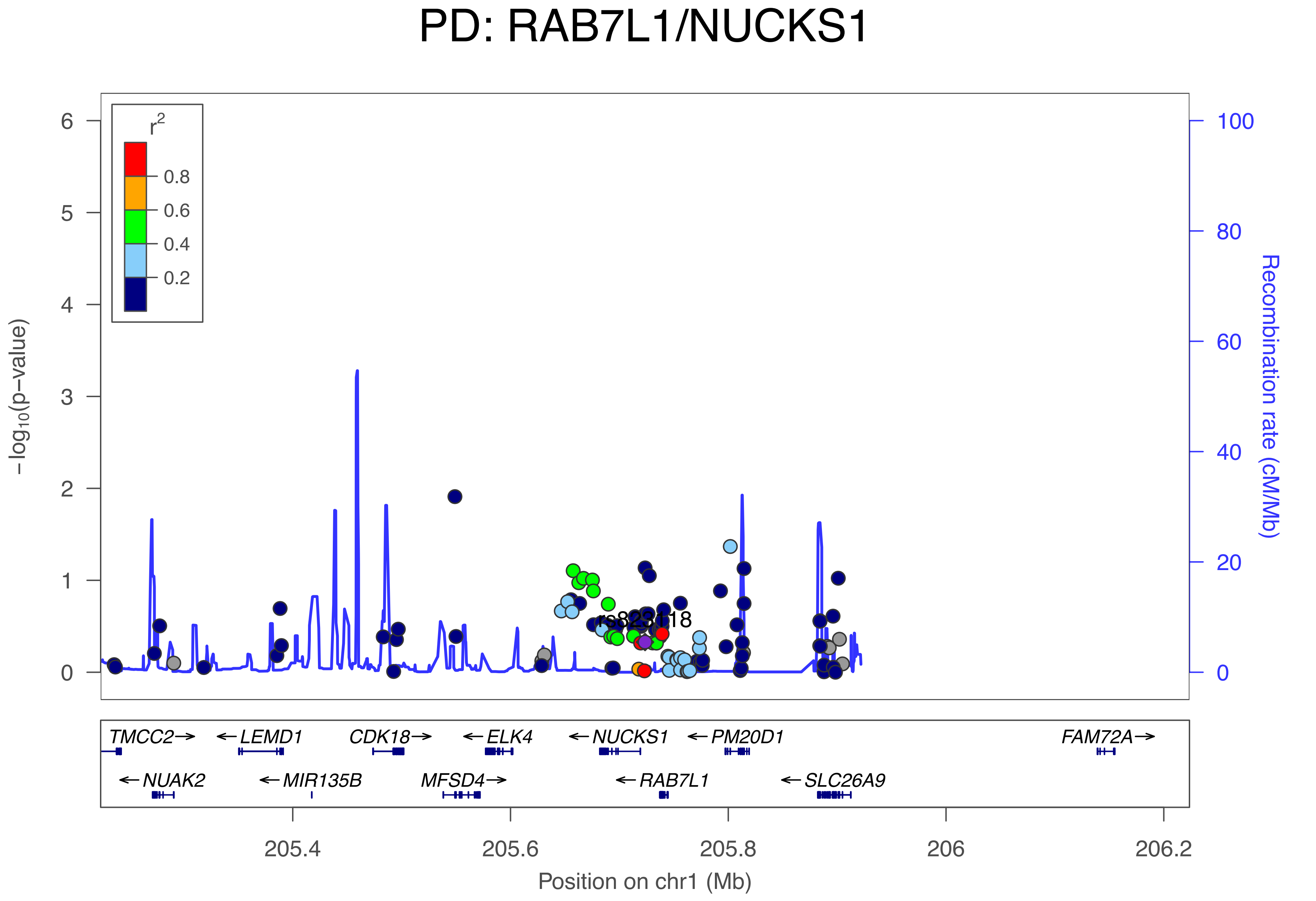

PD: RAB7L1/NUCKS1
r2
0.8
0.6
0.4
0.2
6
5
4
3
2
1
0
-log10(p-value)
100
80
60
40
20
0
Recombination rate (cM/Mb)
rs823118
TMCC2→
←LEMD1
CDK18→
←ELK4
←NUCKS1
←PM20D1
FAM72A→
←NUAK2
←MIR135B
MFSD4→
←RAB7L1
←SLC26A9
205.4
205.6
205.8
206
206.2
Position on chr1 (Mb)

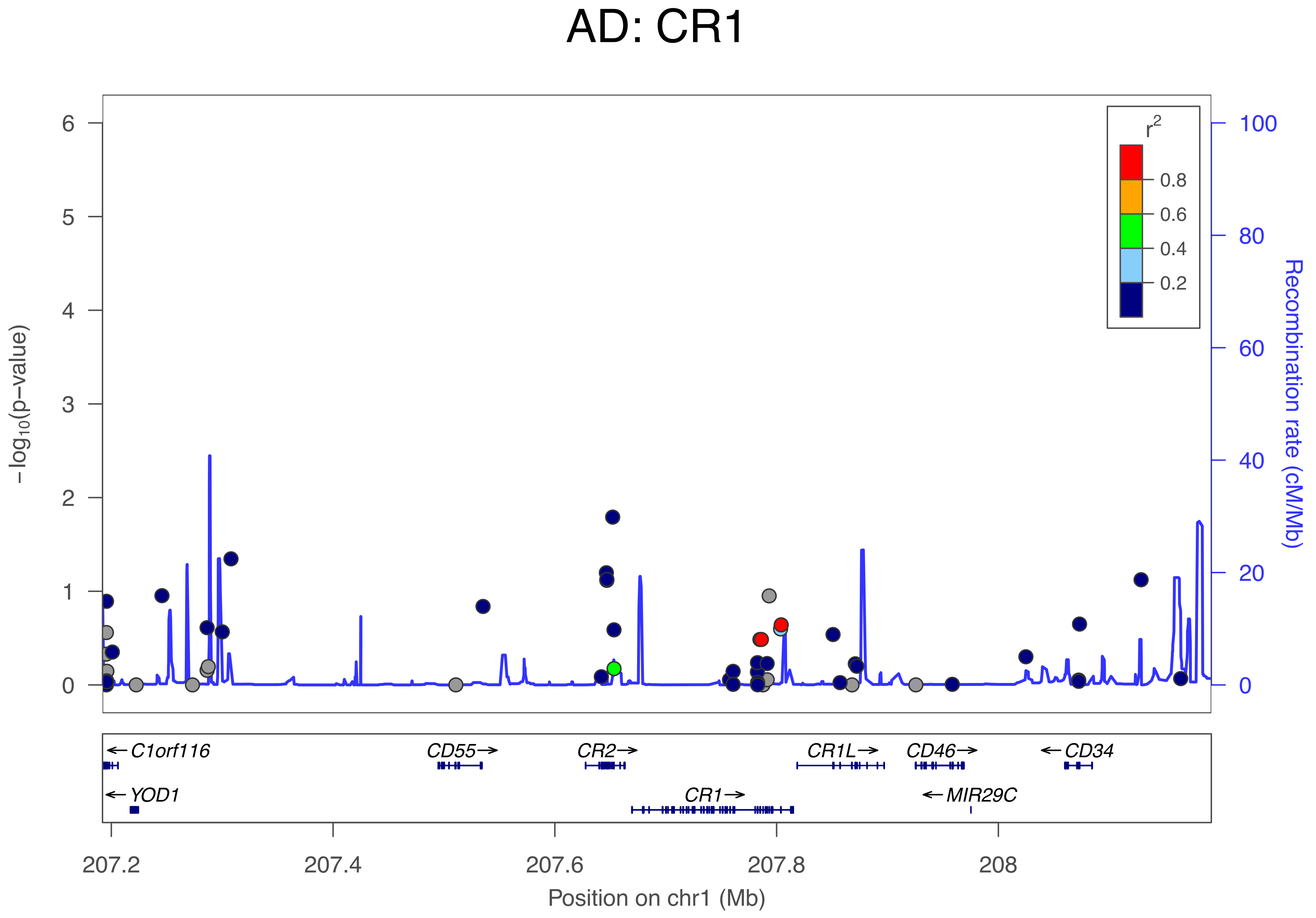
AD: CR1
−log10(p−value)
0
1
2
3
4
5
6
Recombination rate (cM/Mb)
0
20
40
60
80
100
r2
0.8
0.6
0.4
0.2
← C1orf116
← YOD1
CD55 →
CR2 →
CR1 →
CR1L →
CD46 →
← MIR29C
← CD34
207.2
207.4
207.6
207.8
208
Position on chr1 (Mb)

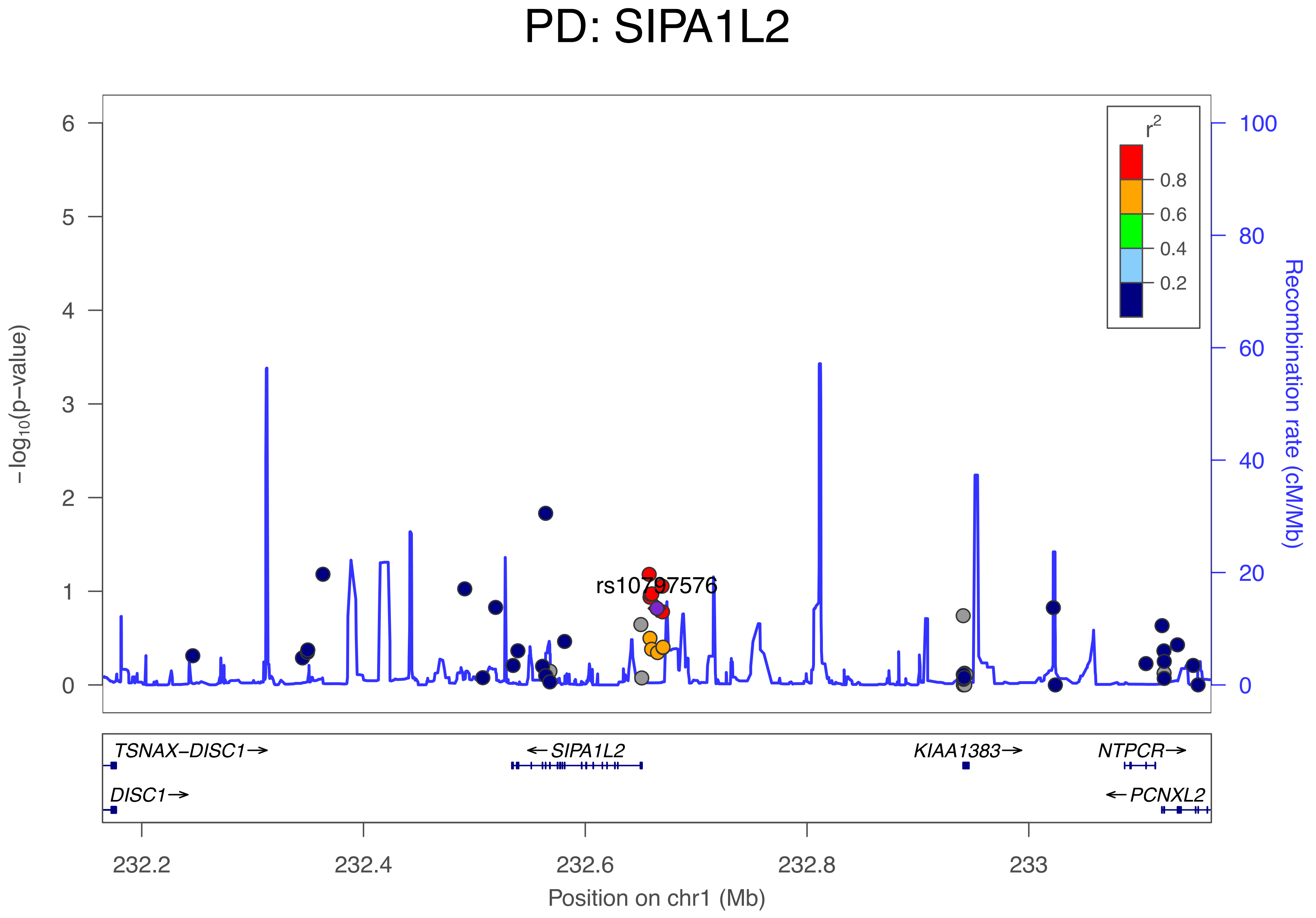
PD: SIPA1L2
−log10(p−value)
Recombination rate (cM/Mb)
r2
0.8
0.6
0.4
0.2
rs10797576
TSNAX−DISC1→
←SIPA1L2
KIAA1383→
NTPCR→
DISC1→
←PCNXL2
232.2
232.4
232.6
232.8
233
Position on chr1 (Mb)
0
1
2
3
4
5
6
0
20
40
60
80
100

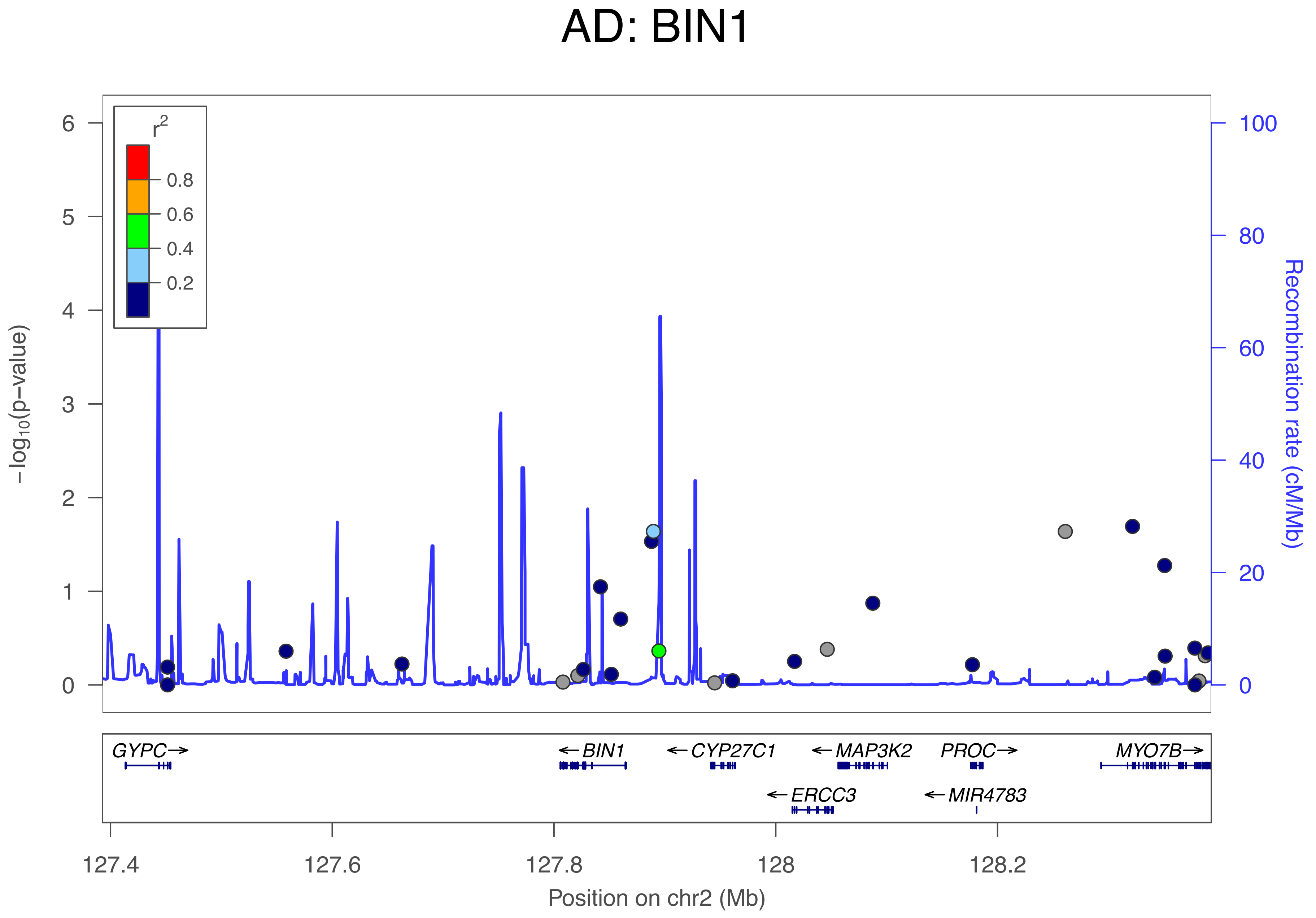
AD: BIN1
r2
0.8
0.6
0.4
0.2
−log10(p−value)
Recombination rate (cM/Mb)
GYPC→
←BIN1
←CYP27C1
←MAP3K2
PROC→
MYO7B→
←ERCC3
←MIR4783
127.4
127.6
127.8
128
128.2
Position on chr2 (Mb)

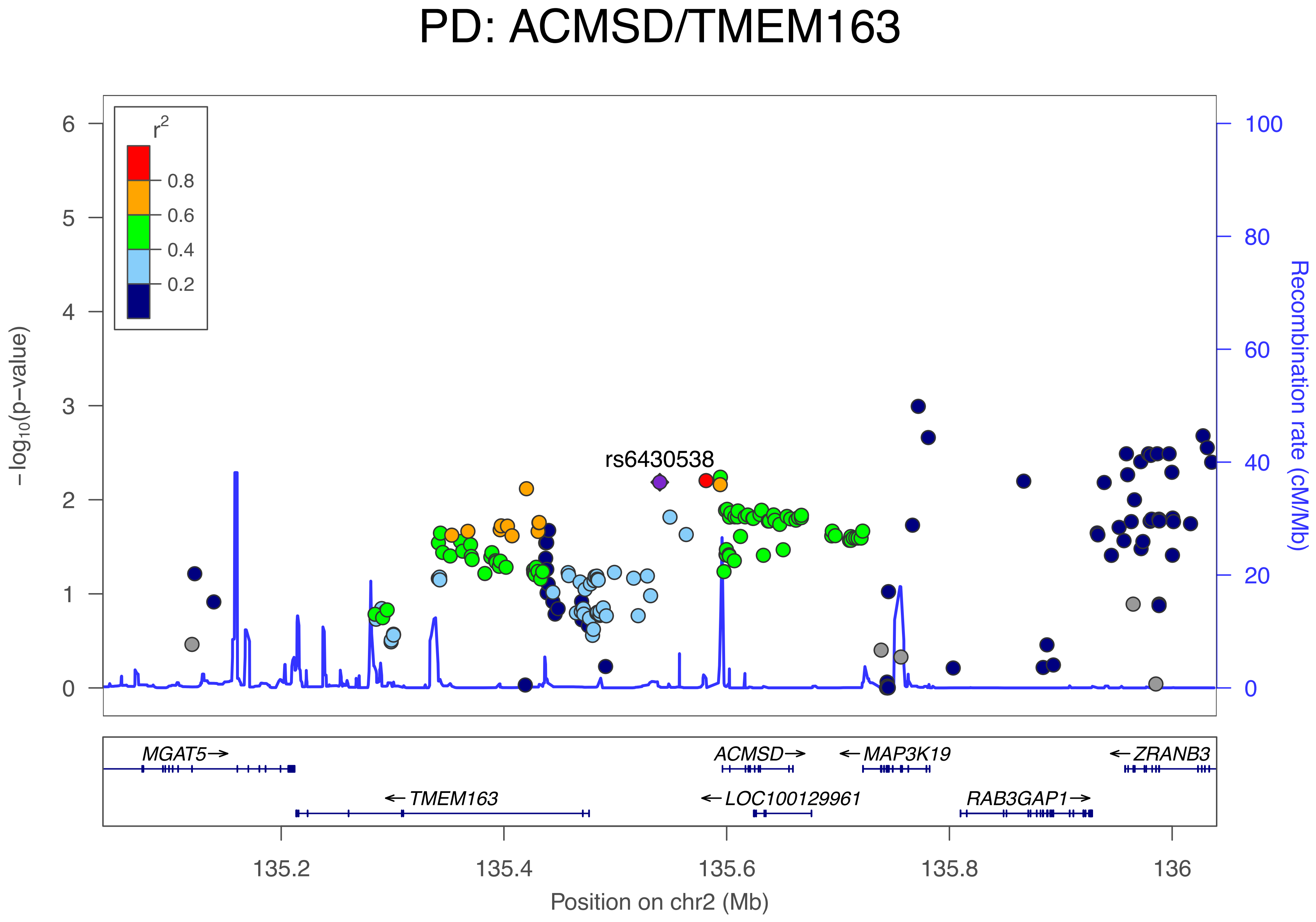

PD: ACMSD/TMEM163
r2
0.8
0.6
0.4
0.2
rs6430538
−log10(p−value)
Recombination rate (cM/Mb)
MGAT5→
←TMEM163
ACMSD→
←LOC100129961
←MAP3K19
RAB3GAP1→
←ZRANB3
135.2
135.4
135.6
135.8
136
Position on chr2 (Mb)

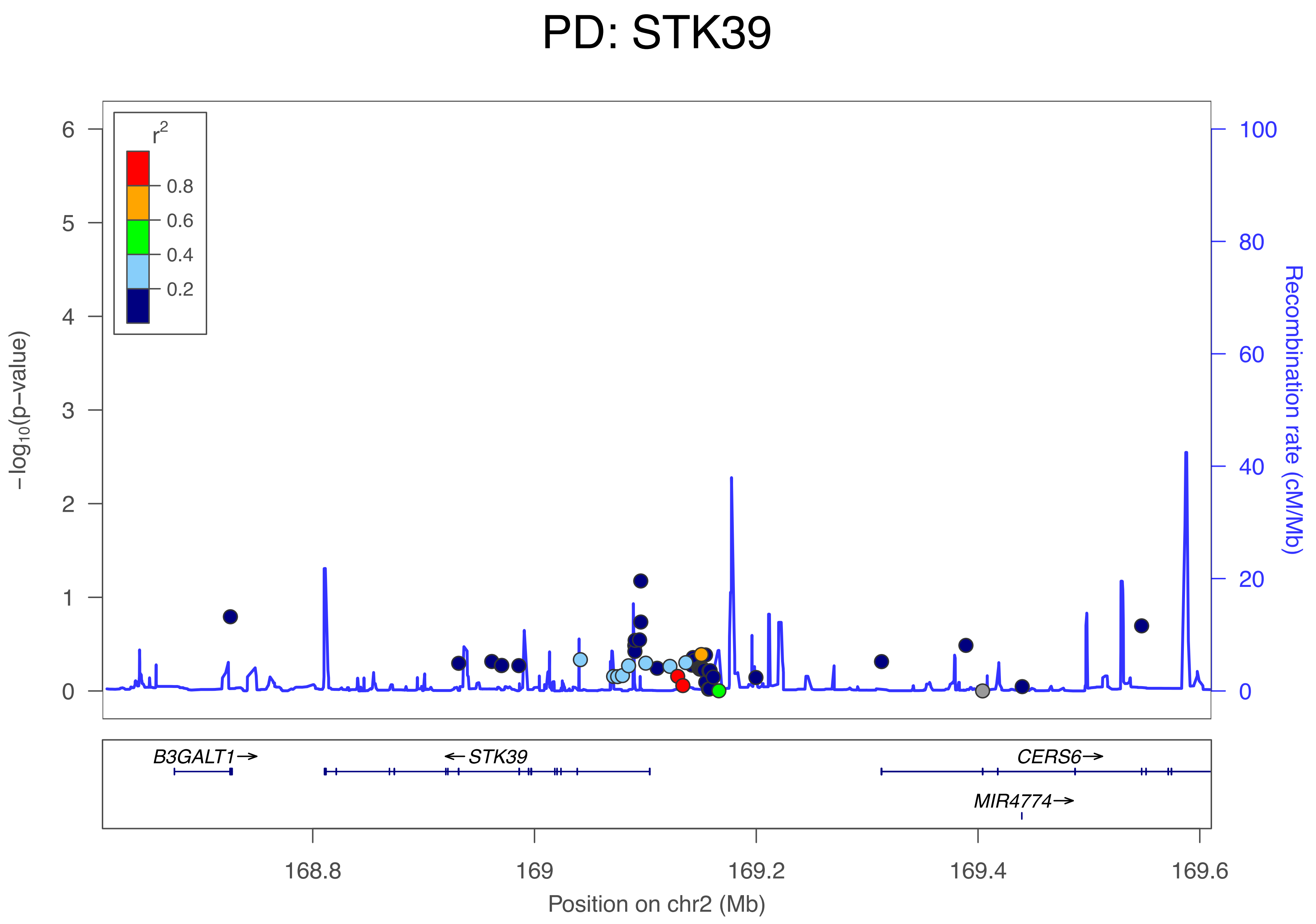
PD: STK39
r2
0.8
0.6
0.4
0.2
6
5
4
3
2
1
0
-log10(p-value)
100
80
60
40
20
0
Recombination rate (cM/Mb)
B3GALT1→
←STK39
CERS6→
MIR4774→
168.8
169
169.2
169.4
169.6
Position on chr2 (Mb)

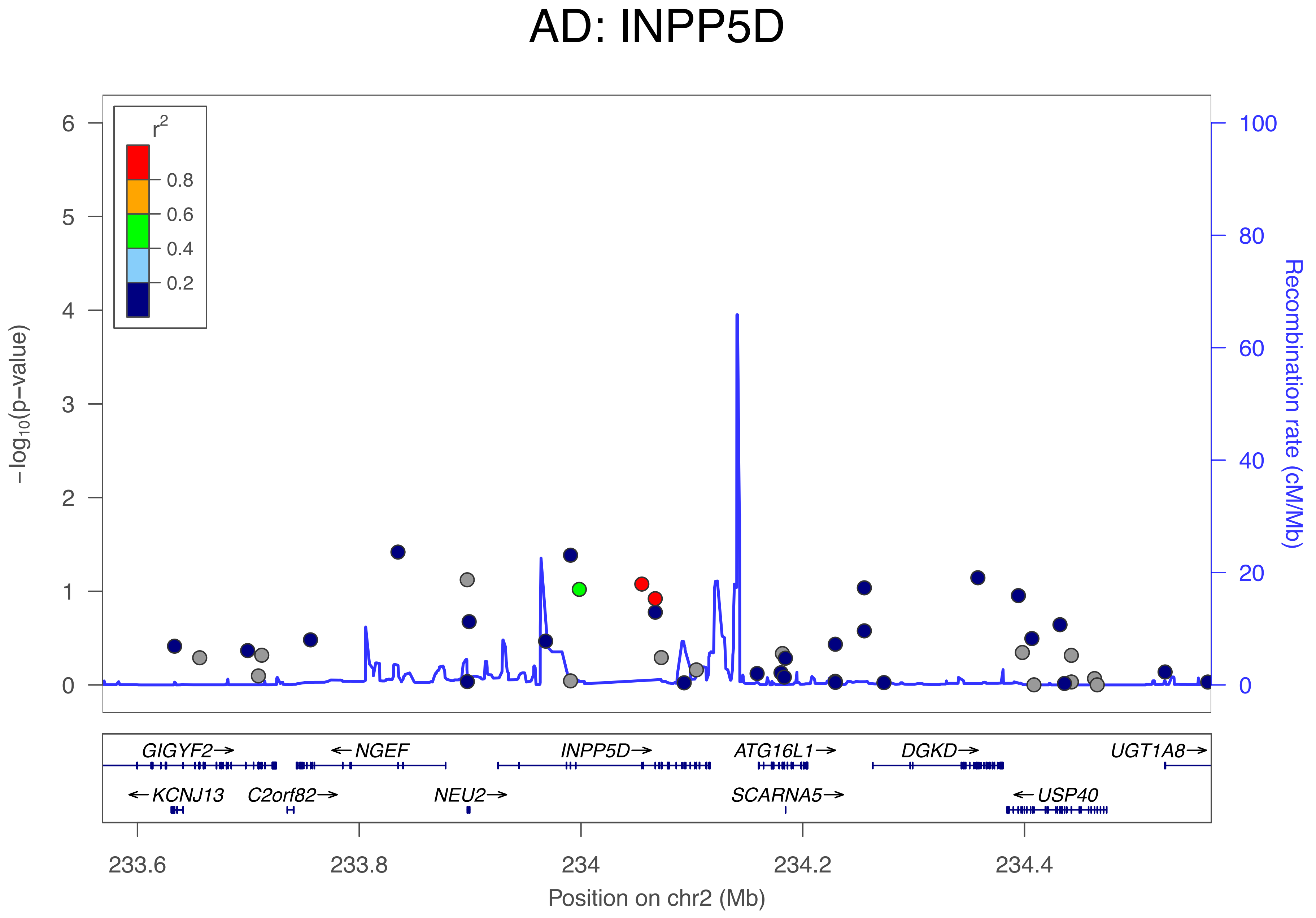
AD: INPP5D
r2
0.8
0.6
0.4
0.2
−log10(p−value)
Recombination rate (cM/Mb)
0
1
2
3
4
5
6
0
20
40
60
80
100
GIGYF2→
←NGEF
INPP5D→
ATG16L1→
DGKD→
UGT1A8→
←KCNJ13
C2orf82→
NEU2→
SCARNA5→
←USP40
233.6
233.8
234
234.2
234.4
Position on chr2 (Mb)

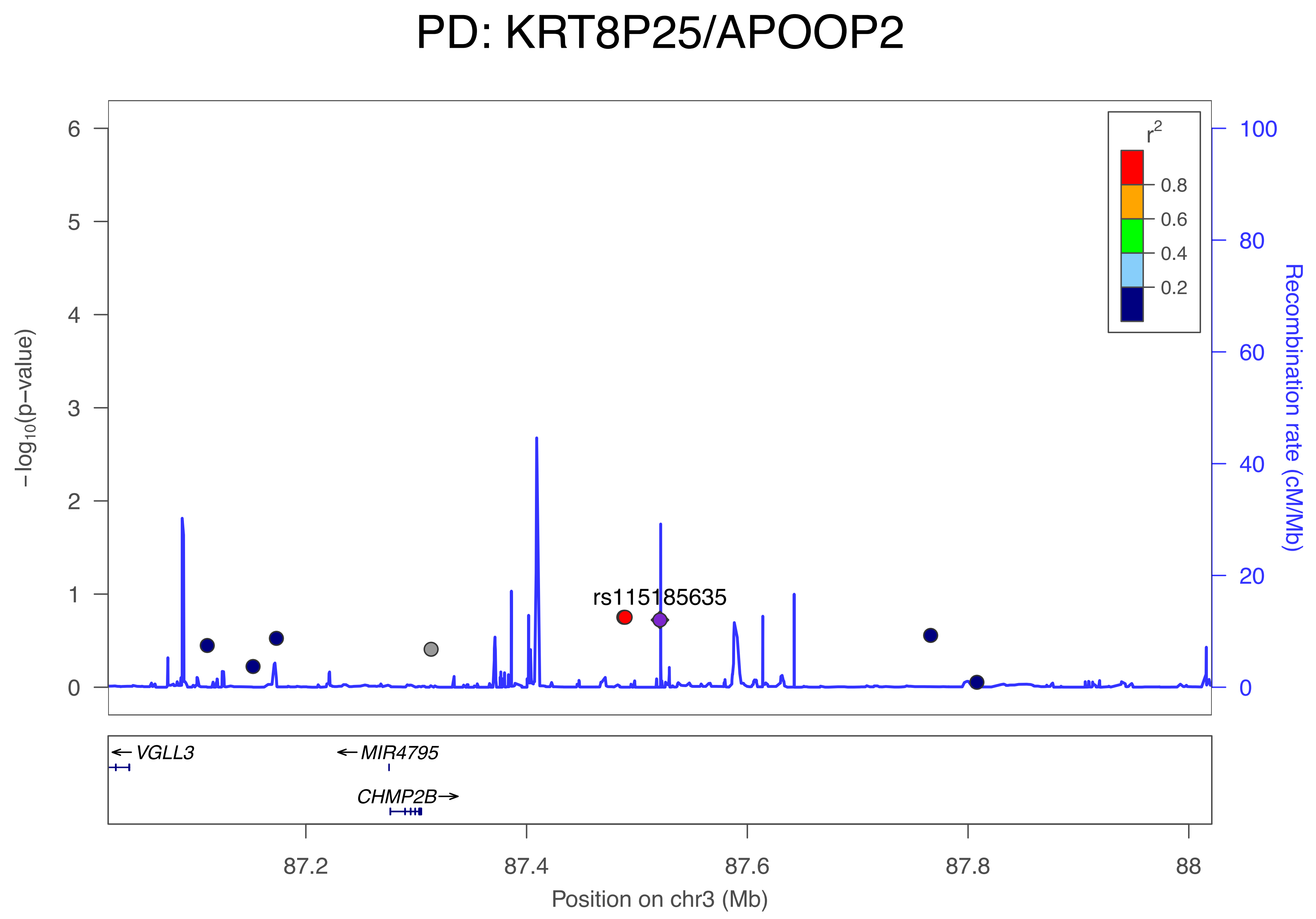

PD: KRT8P25/APOOP2
r2
0.8
0.6
0.4
0.2
rs115185635
−log10(p−value)
Recombination rate (cM/Mb)
VGLL3
MIR4795
CHMP2B
87.2
87.4
87.6
87.8
88
Position on chr3 (Mb)

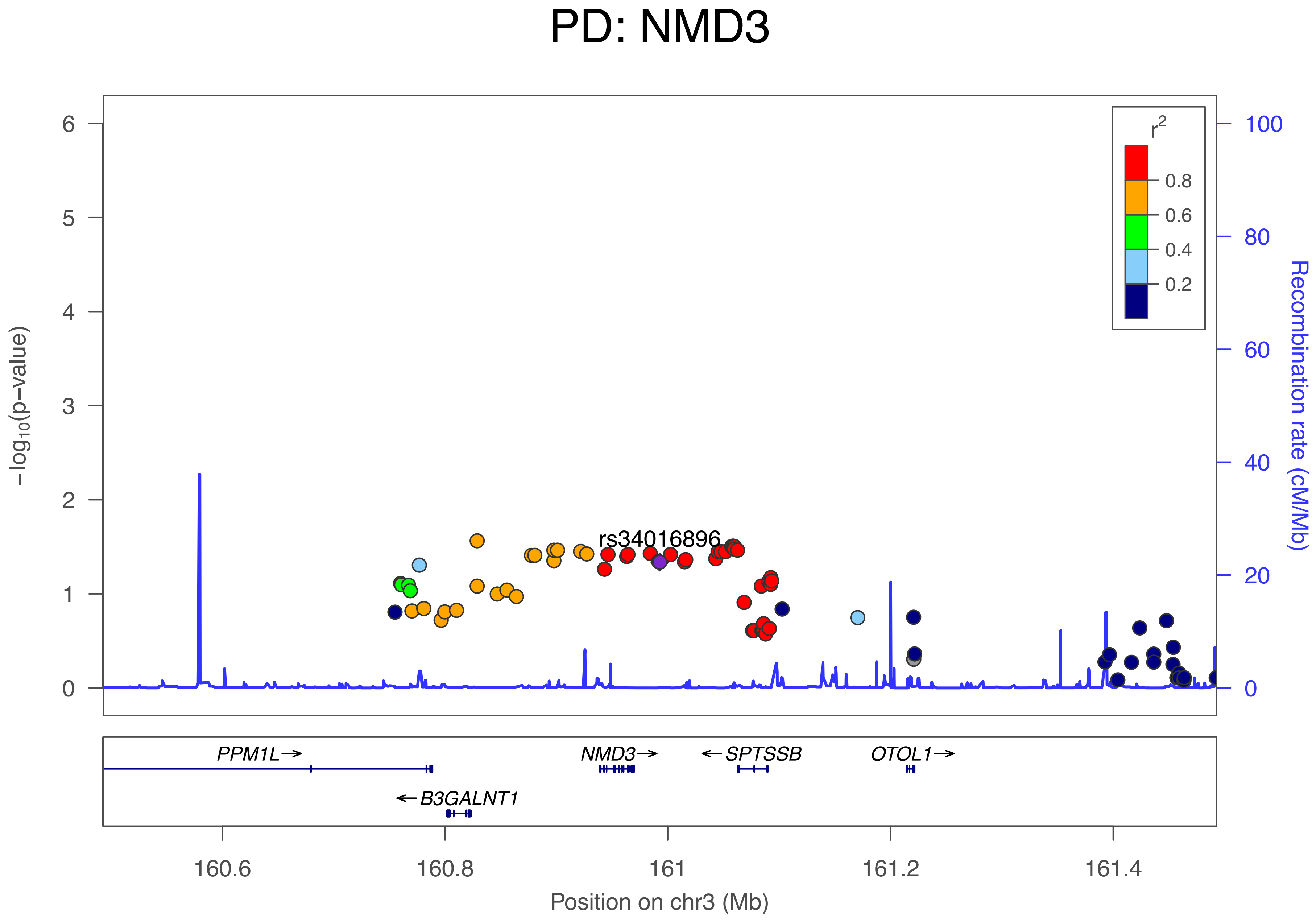
PD: NMD3
−log10(p−value)
Recombination rate (cM/Mb)
r2
0.8
0.6
0.4
0.2
rs34016896
PPM1L→
NMD3→
←SPTSSB
OTOL1→
←B3GALNT1
160.6
160.8
161
161.2
161.4
Position on chr3 (Mb)
0
1
2
3
4
5
6
0
20
40
60
80
100

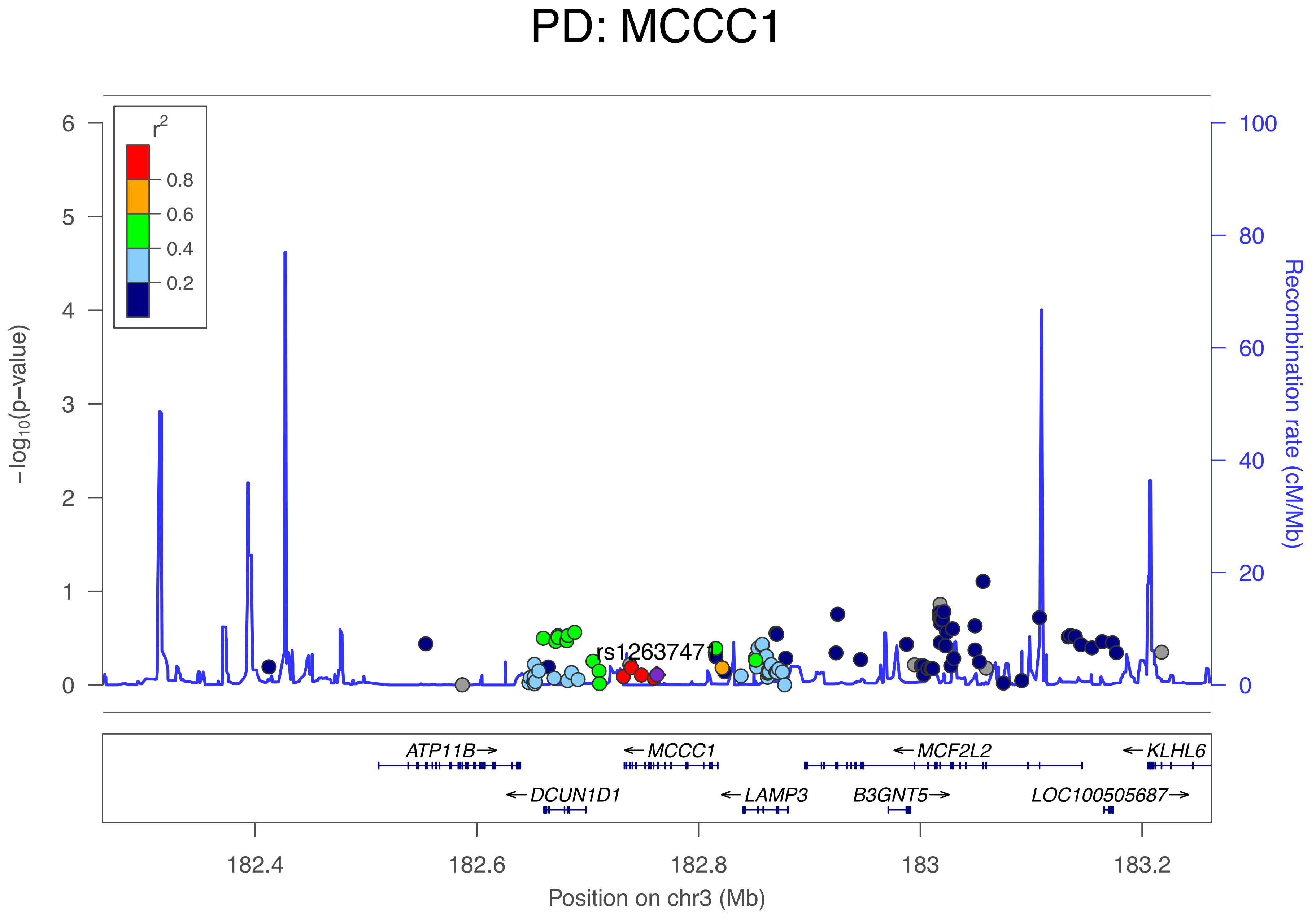

PD: MCCC1
r2
0.8
0.6
0.4
0.2
rs12637471
−log10(p−value)
Recombination rate (cM/Mb)
ATP11B→
←MCCC1
←MCF2L2
←KLHL6
←DCUN1D1
←LAMP3
B3GNT5→
LOC100505687→
182.4
182.6
182.8
183
183.2
Position on chr3 (Mb)

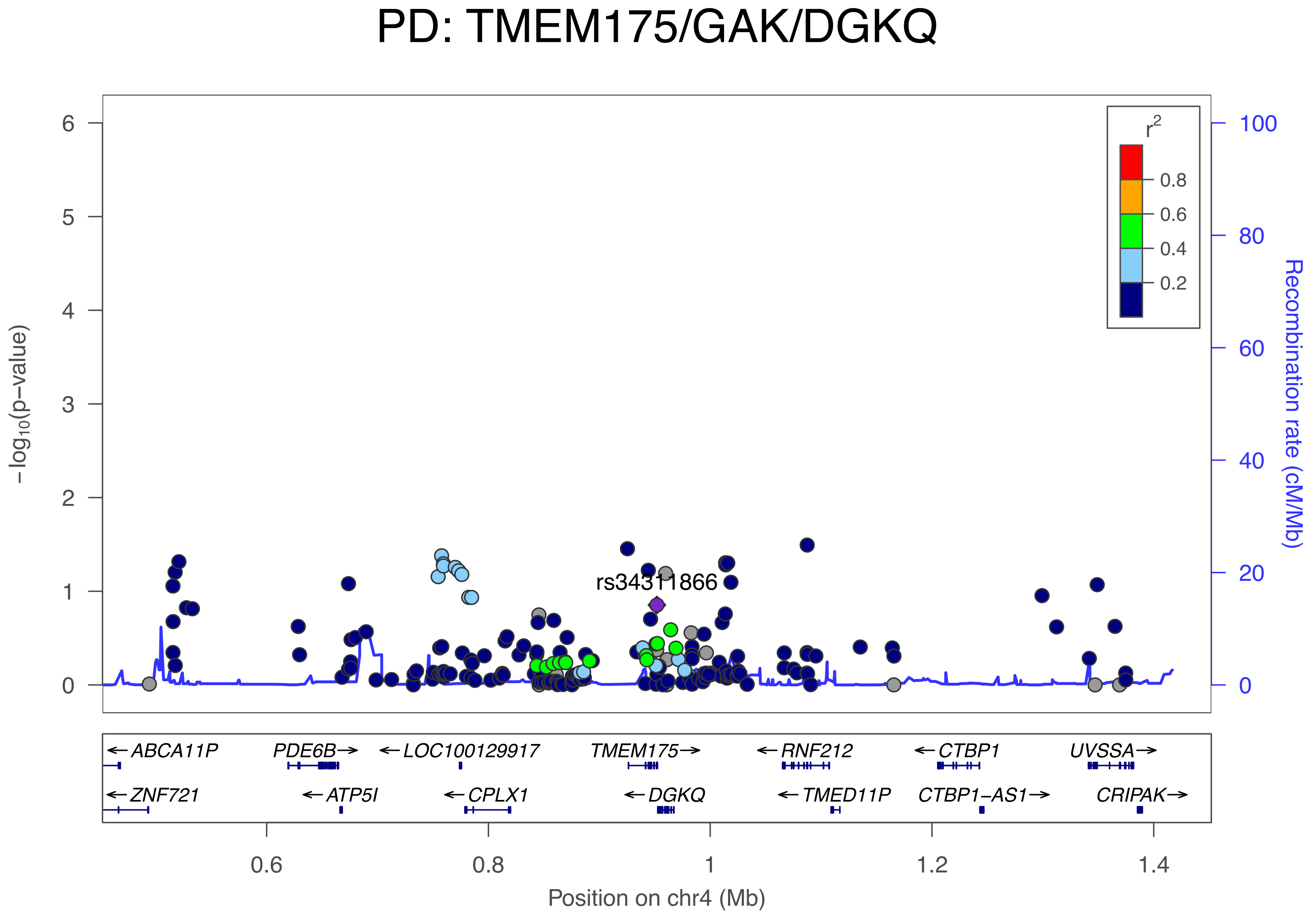

PD: TMEM175/GAK/DGKQ
−log10(p−value)
Recombination rate (cM/Mb)
r2
0.8
0.6
0.4
0.2
rs34311866
ABCA11P
ZNF721
PDE6B
ATP5I
LOC100129917
CPLX1
TMEM175
DGKQ
RNF212
TMED11P
CTBP1
CTBP1−AS1
UVSSA
CRIPAK
Position on chr4 (Mb)
0.6
0.8
1
1.2
1.4
0
1
2
3
4
5
6
20
40
60
80
100

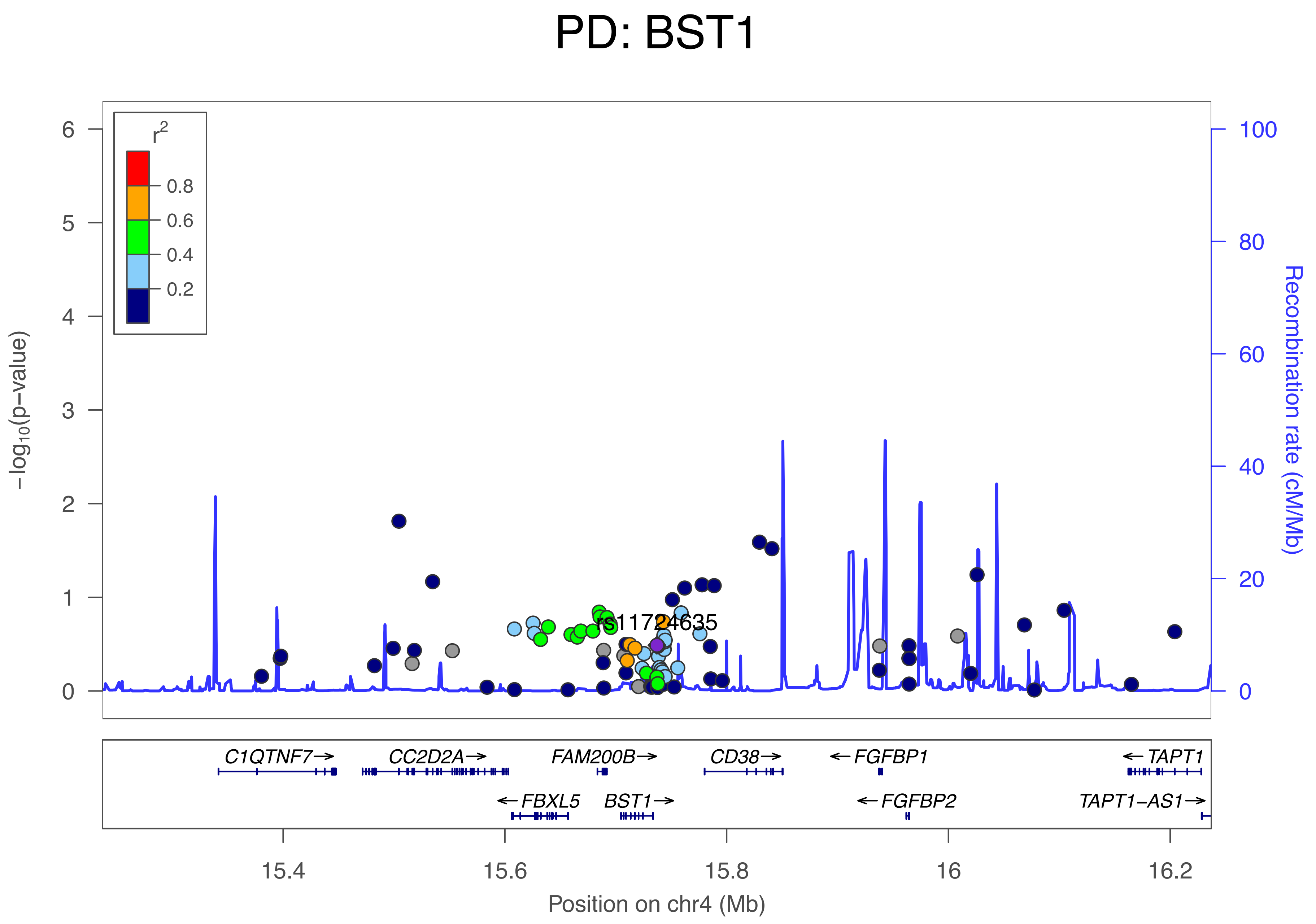

PD: BST1
r2
0.8
0.6
0.4
0.2
rs11724635
-log10(p-value)
Recombination rate (cM/Mb)
0
1
2
3
4
5
6
0
20
40
60
80
100
C1QTNF7→
CC2D2A→
FAM200B→
CD38→
←FGFBP1
←TAPT1
←FBXL5
BST1→
←FGFBP2
TAPT1-AS1→
15.4
15.6
15.8
16
16.2
Position on chr4 (Mb)

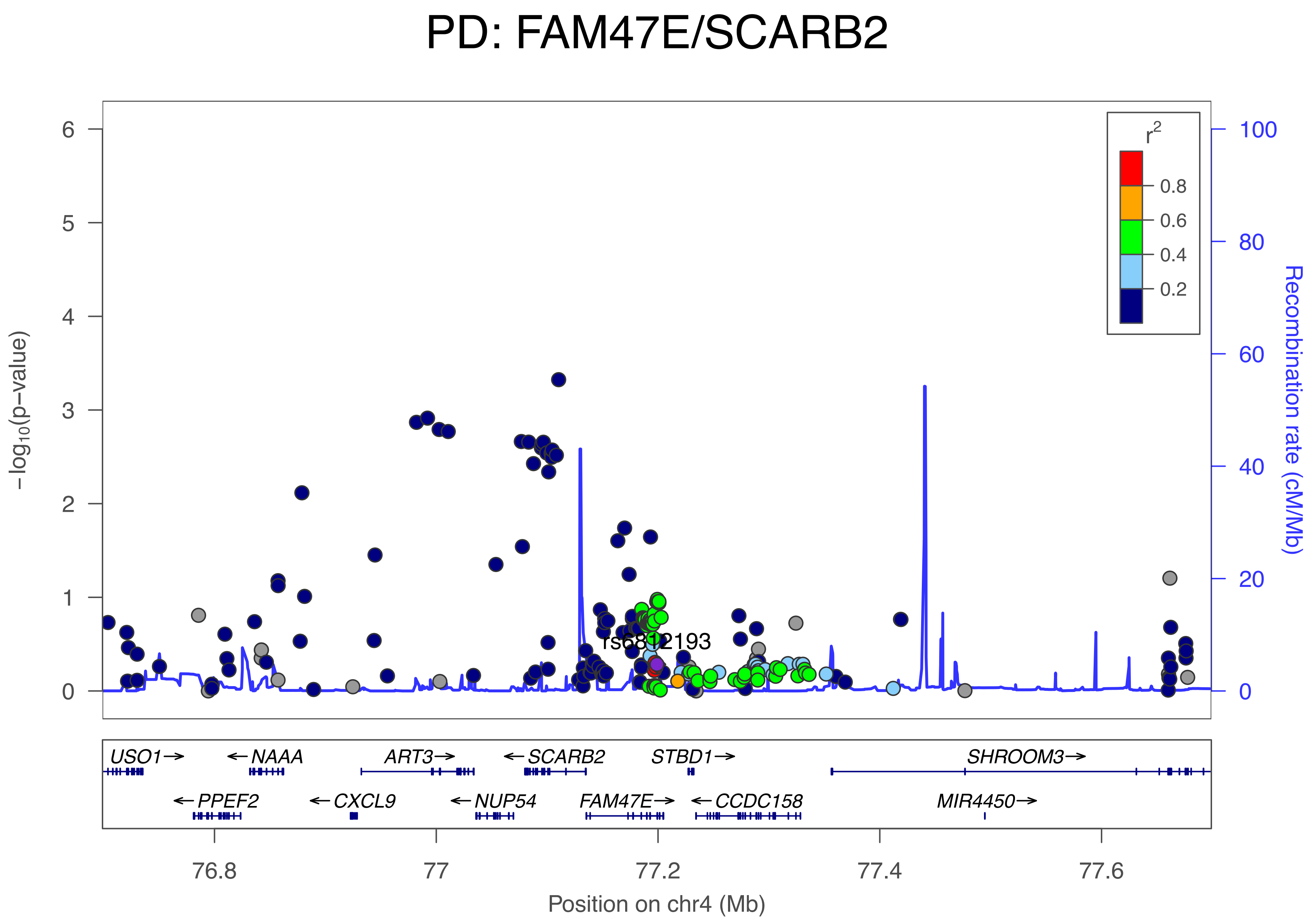
PD: FAM47E/SCARB2
-log10(p-value)
Recombination rate (cM/Mb)
r2
0.8
0.6
0.4
0.2
rs6812193
USO1→
←NAAA
ART3→
←SCARB2
STBD1→
SHROOM3→
←PPEF2
←CXCL9
←NUP54
FAM47E→
←CCDC158
MIR4450→
76.8
77
77.2
77.4
77.6
Position on chr4 (Mb)

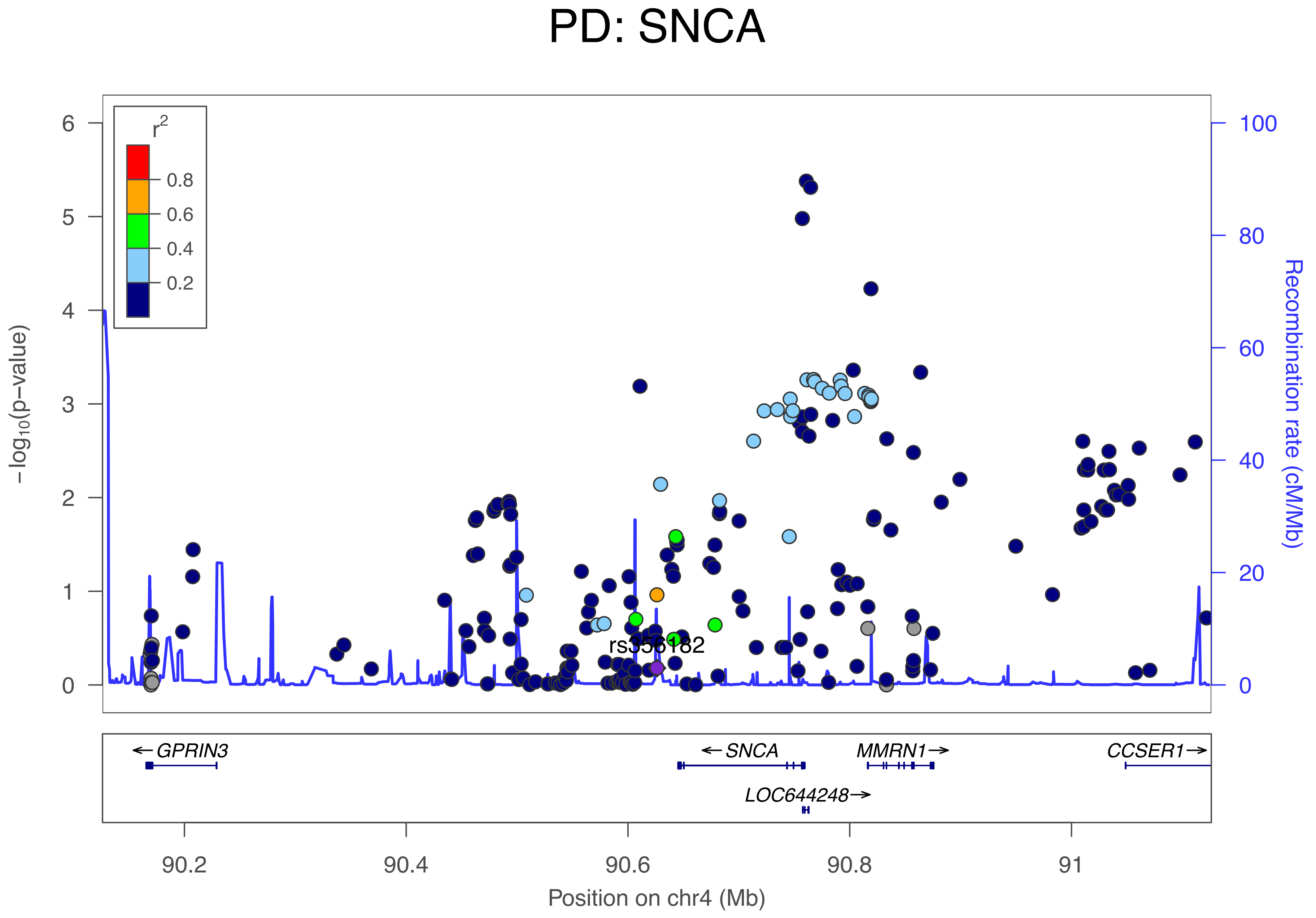
PD: SNCA
r2
0.8
0.6
0.4
0.2
−log10(p−value)
0
1
2
3
4
5
6
Recombination rate (cM/Mb)
0
20
40
60
80
100
rs356182
GPRIN3
SNCA
MMRN1
CCSER1
LOC644248
90.2
90.4
90.6
90.8
91
Position on chr4 (Mb)

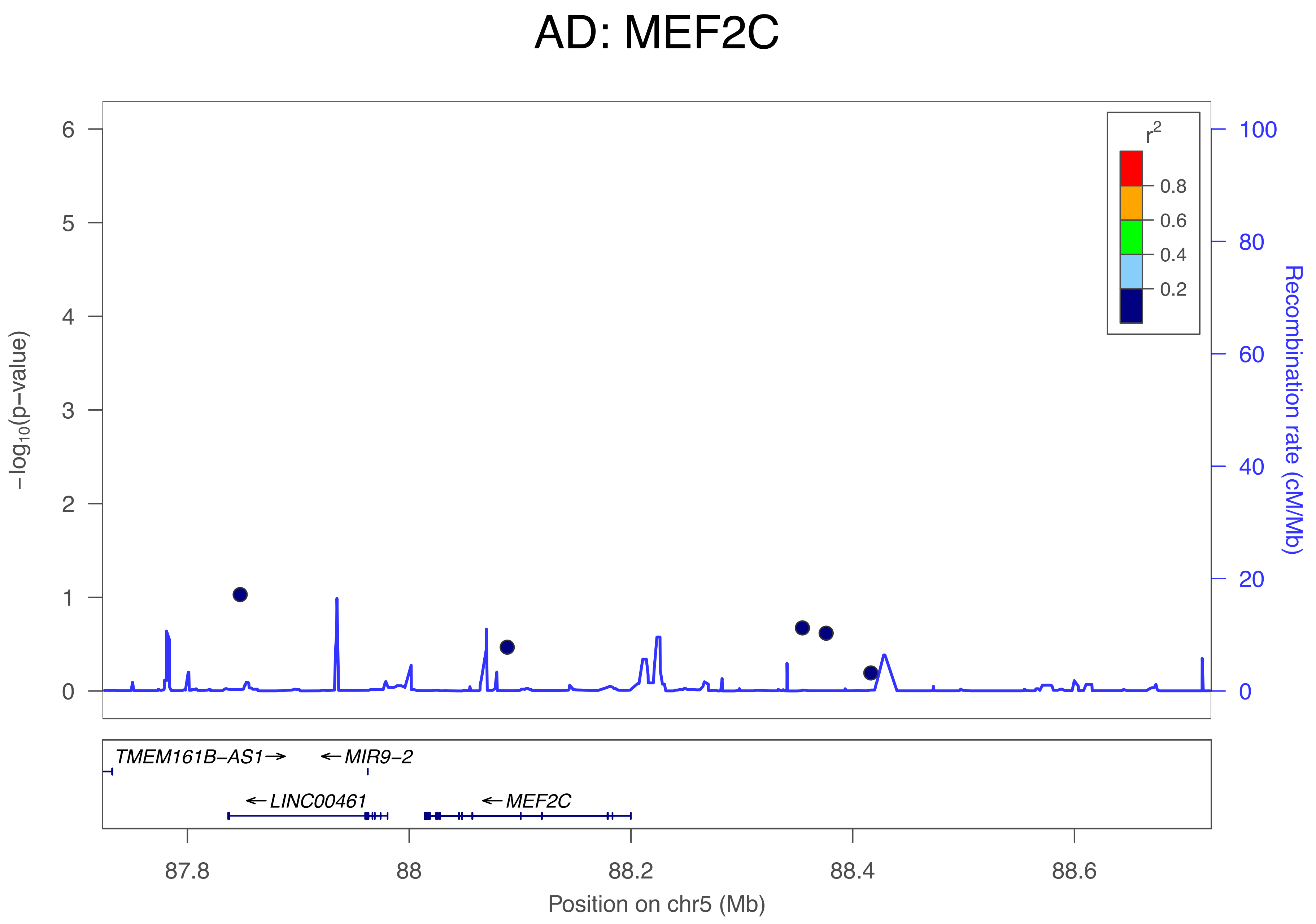
AD: MEF2C
-log10(p-value)
0
1
2
3
4
5
6
Recombination rate (cM/Mb)
0
20
40
60
80
100
r2
0.8
0.6
0.4
0.2
TMEM161B-AS1→
←MIR9-2
←LINC00461
←MEF2C
87.8
88
88.2
88.4
88.6
Position on chr5 (Mb)

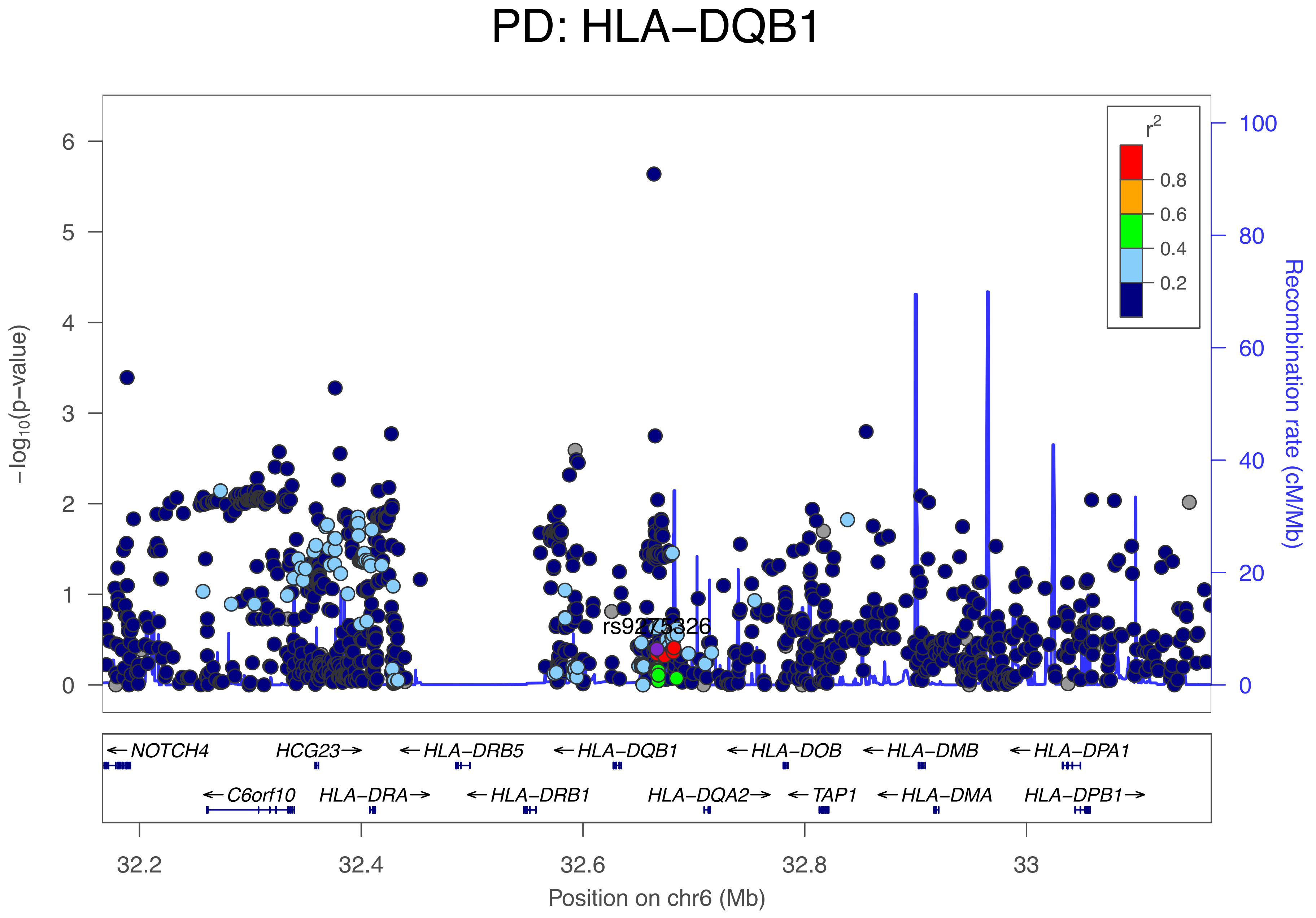

PD: HLA−DQB1
−log10(p−value)
Recombination rate (cM/Mb)
r2
0.8
0.6
0.4
0.2
rs9275326
←NOTCH4
HCG23→
←HLA−DRB5
←HLA−DQB1
←HLA−DOB
←HLA−DMB
←HLA−DPA1
←C6orf10
HLA−DRA→
←HLA−DRB1
HLA−DQA2→
←TAP1
←HLA−DMA
HLA−DPB1→
Position on chr6 (Mb)

AD: CD2AP

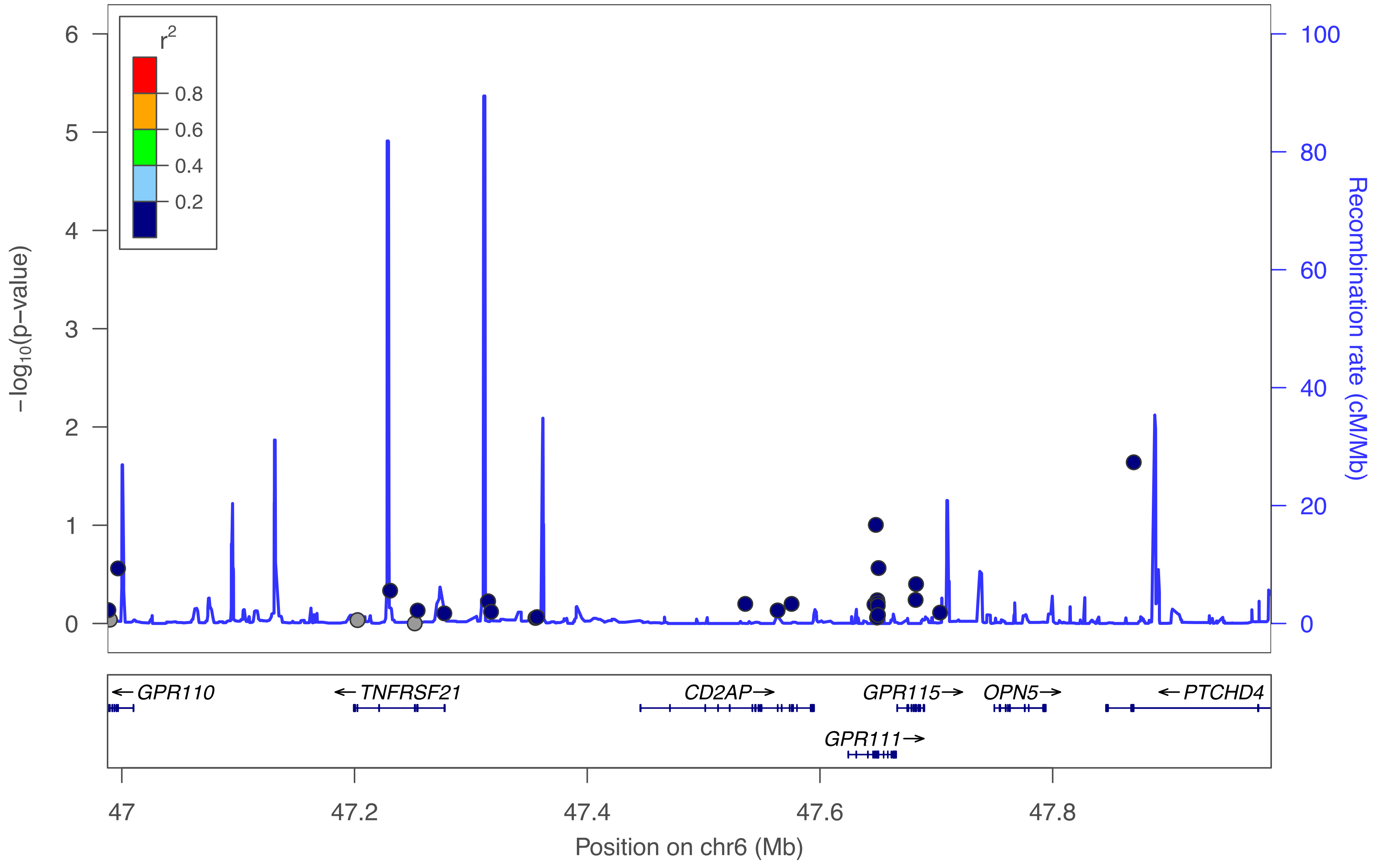

r²
0.8
0.6
0.4
0.2
6
5
4
3
2
1
0
−log10(p−value)
100
80
60
40
20
0
Recombination rate (cM/Mb)
← GPR110
← TNFRSF21
CD2AP →
GPR115 →
OPN5 →
← PTCHD4
GPR111 →
47
47.2
47.4
47.6
47.8
Position on chr6 (Mb)

PD: GPNMB

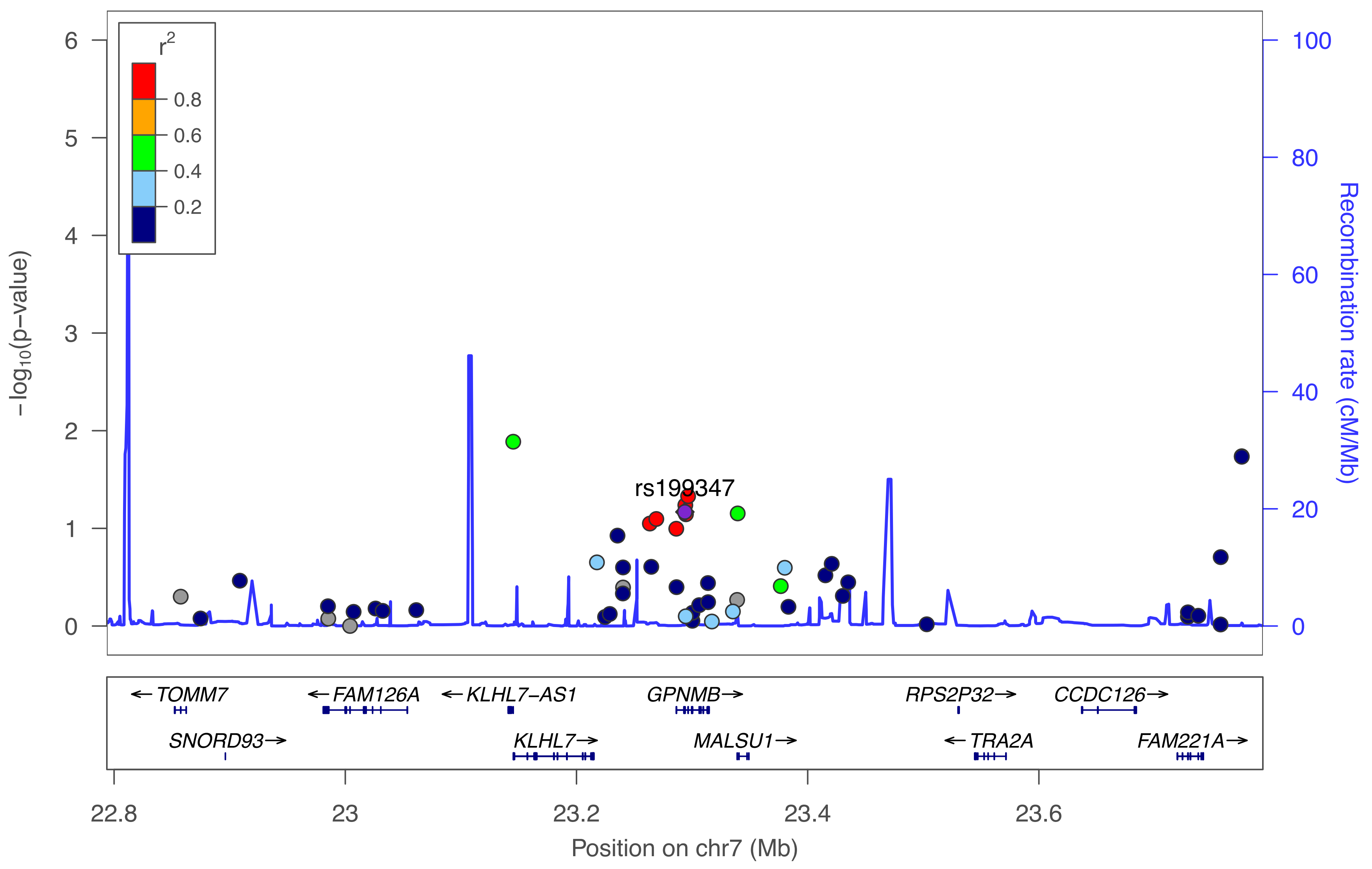

r2
0.8
0.6
0.4
0.2
rs199347
−log10(p−value)
Recombination rate (cM/Mb)
← TOMM7
← FAM126A
← KLHL7−AS1
GPNMB→
RPS2P32→
CCDC126→
SNORD93→
KLHL7→
MALSU1→
← TRA2A
FAM221A→
22.8
23
23.2
23.4
23.6
Position on chr7 (Mb)

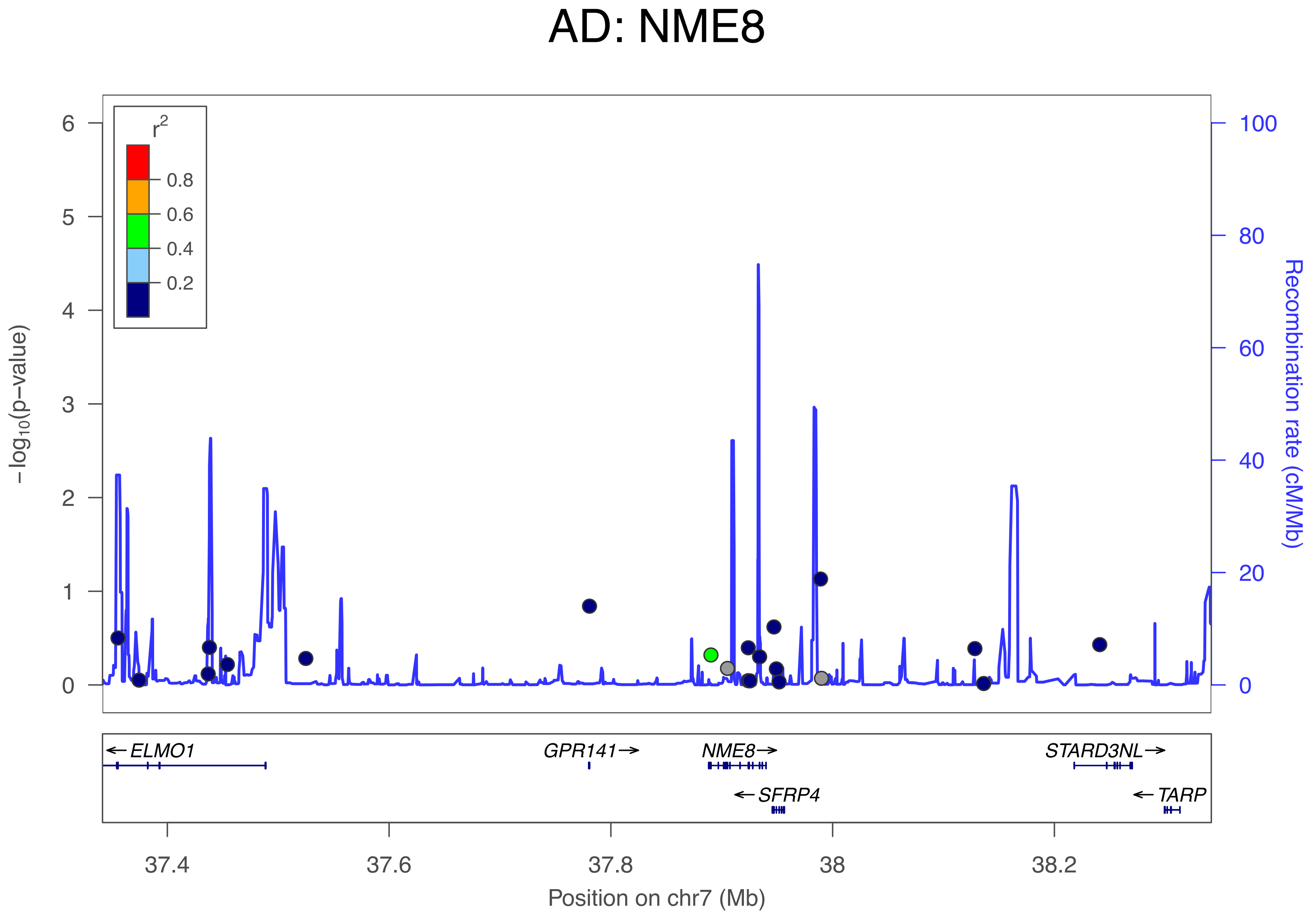
AD: NME8
r2
0.8
0.6
0.4
0.2
6
5
4
3
2
1
0
−log10(p−value)
100
80
60
40
20
0
Recombination rate (cM/Mb)
ELMO1
GPR141
NME8
SFRP4
STARD3NL
TARP
37.4
37.6
37.8
38
38.2
Position on chr7 (Mb)

AD: ZCWPW1

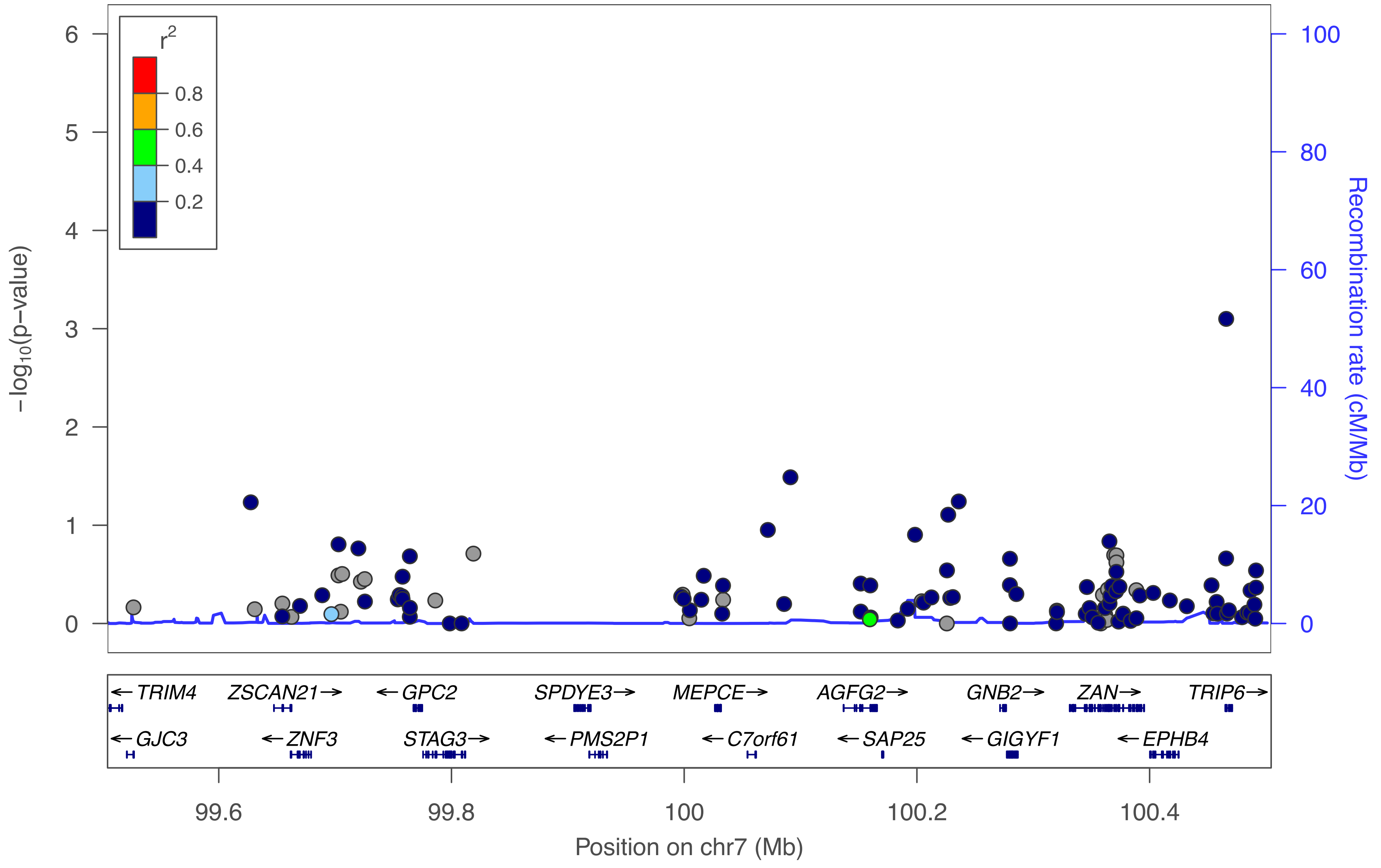
r2
0.8
0.6
0.4
0.2
6
5
4
3
2
1
0
−log10(p−value)
100
80
60
40
20
0
Recombination rate (cM/Mb)
← TRIM4
ZSCAN21→
← GPC2
SPDYE3→
MEPCE→
AGFG2→
GNB2→
ZAN→
TRIP6→
← GJC3
← ZNF3
STAG3→
← PMS2P1
← C7orf61
← SAP25
← GIGYF1
← EPHB4
99.6
99.8
100
100.2
100.4
Position on chr7 (Mb)

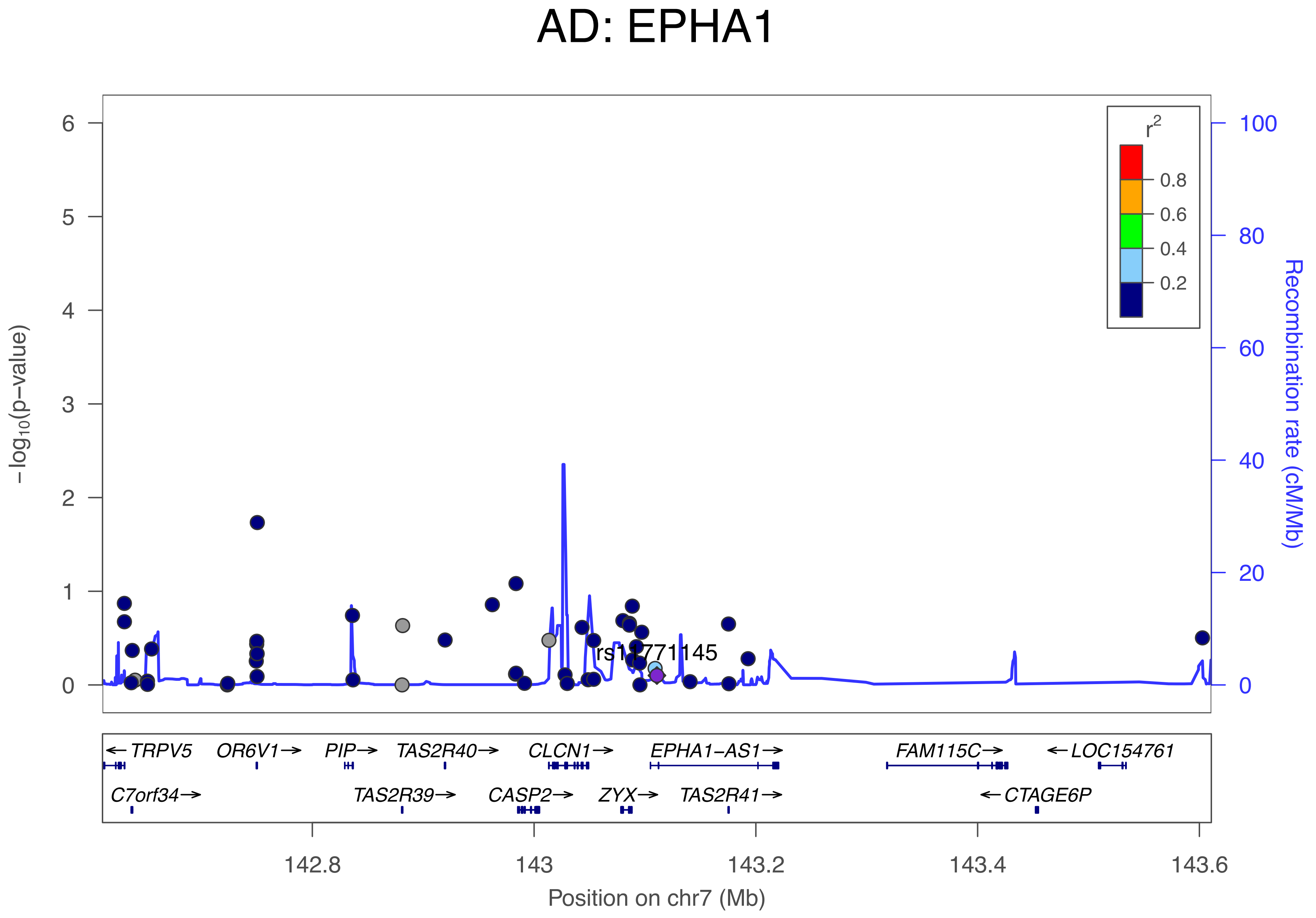

AD: EPHA1
−log10(p−value)
0
1
2
3
4
5
6
Recombination rate (cM/Mb)
0
20
40
60
80
100
r2
0.8
0.6
0.4
0.2
rs11771145
← TRPV5
OR6V1→
PIP→
TAS2R40→
CLCN1→
EPHA1−AS1→
FAM115C→
← LOC154761
C7orf34→
TAS2R39→
CASP2→
ZYX→
TAS2R41→
← CTAGE6P
142.8
143
143.2
143.4
143.6
Position on chr7 (Mb)

PD: FGF20

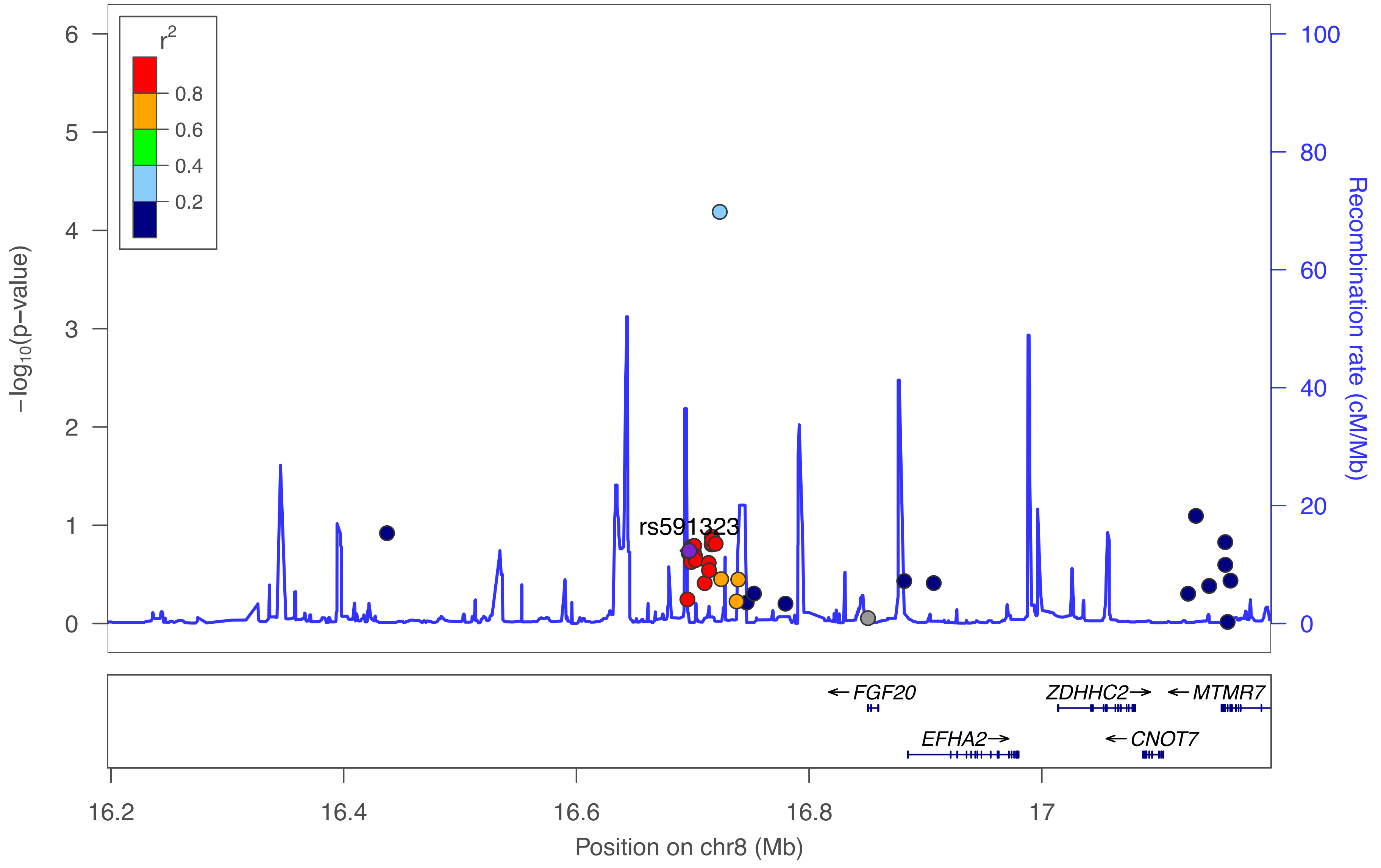

r2
0.8
0.6
0.4
0.2
rs591323
−log10(p−value)
Recombination rate (cM/Mb)
FGF20
EFHA2
ZDHHC2
CNOT7
MTMR7
16.2
16.4
16.6
16.8
17
Position on chr8 (Mb)

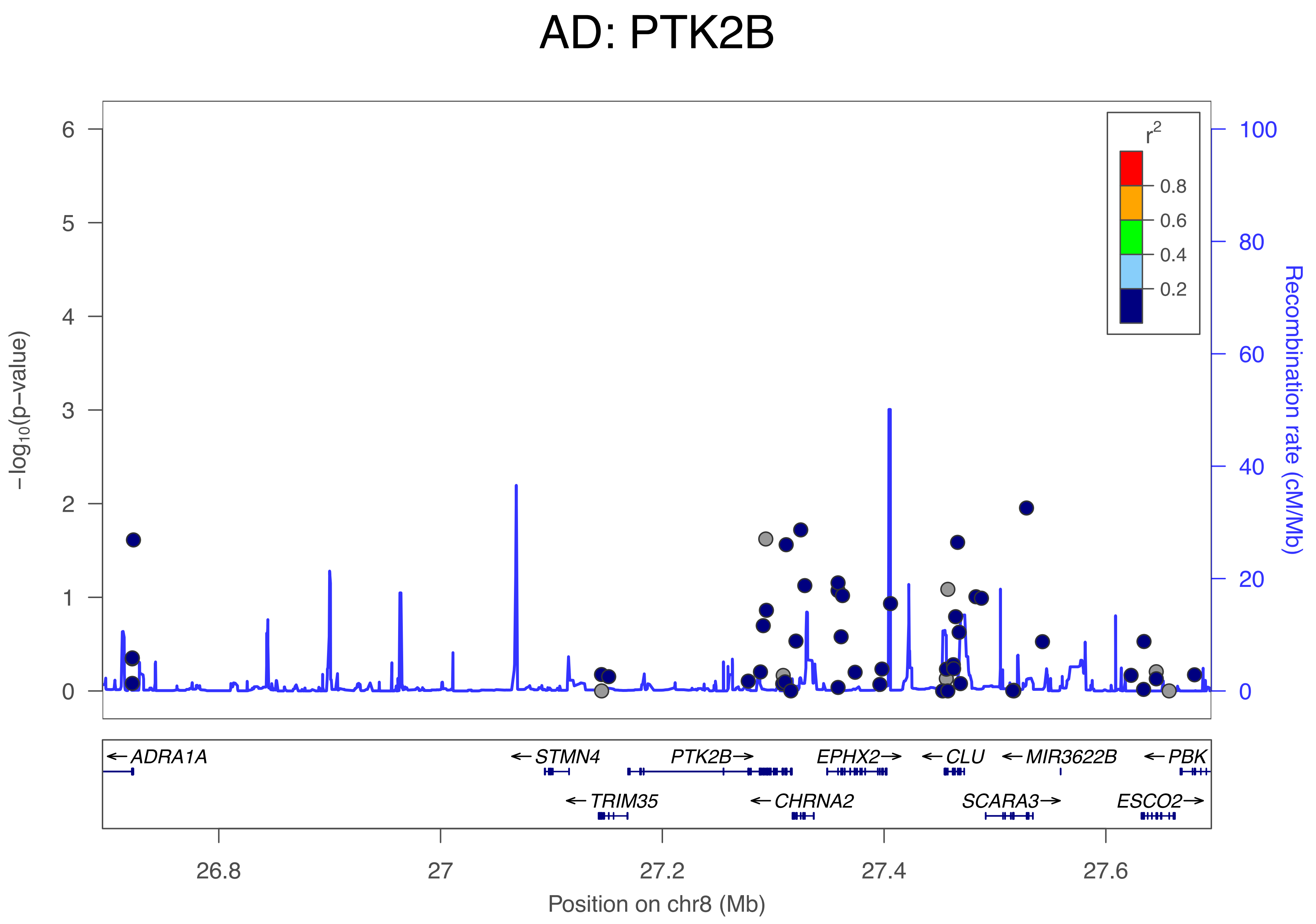

AD: PTK2B
−log10(p−value)
Recombination rate (cM/Mb)
r2
0.8
0.6
0.4
0.2
0
1
2
3
4
5
6
0
20
40
60
80
100
ADRA1A
STMN4
TRIM35
PTK2B
CHRNA2
EPHX2
CLU
SCARA3
MIR3622B
PBK
ESCO2
26.8
27
27.2
27.4
27.6
Position on chr8 (Mb)

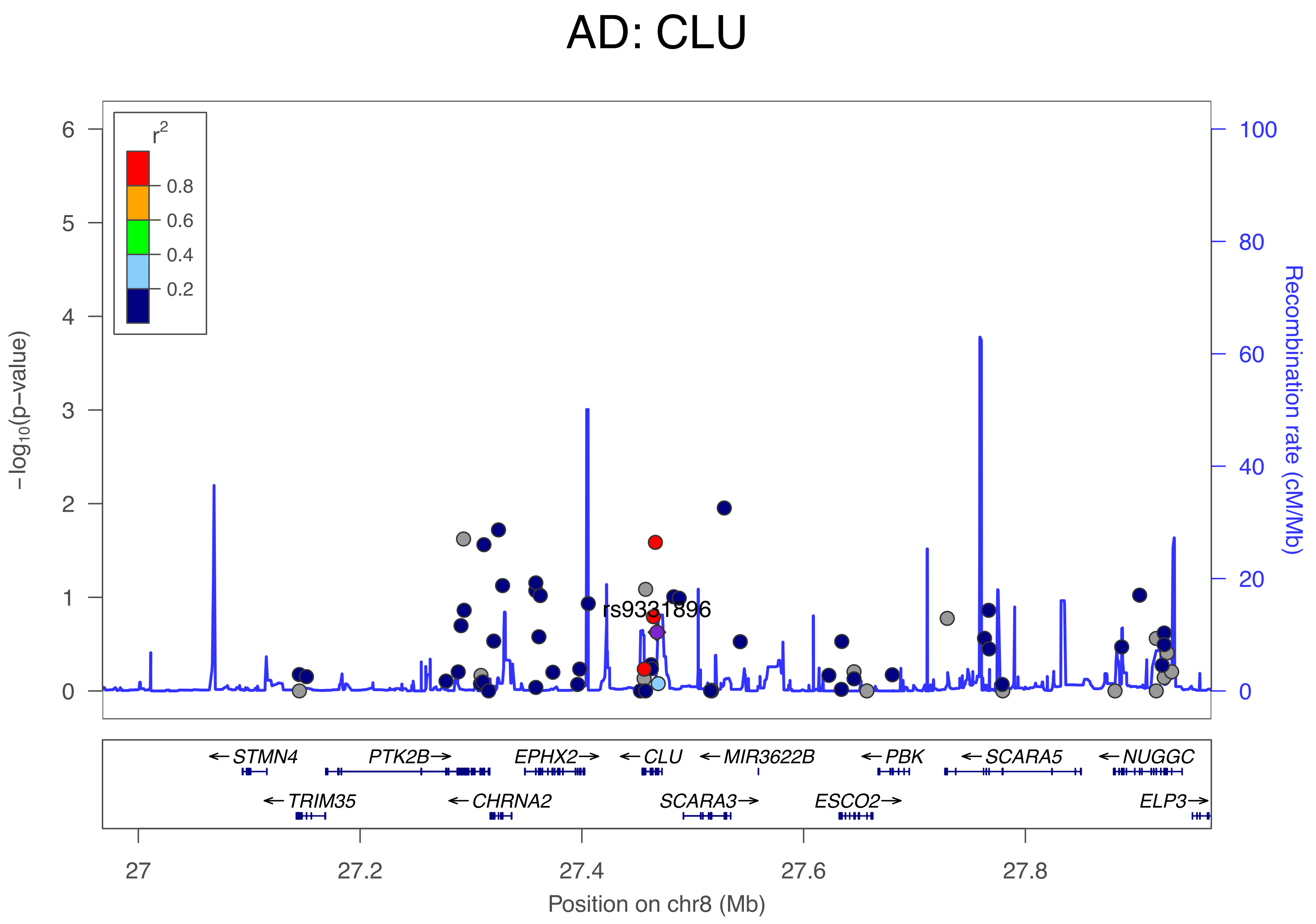

AD: CLU
r2
0.8
0.6
0.4
0.2
rs9331896
-log10(p-value)
Recombination rate (cM/Mb)
0
1
2
3
4
5
6
0
20
40
60
80
100
STMN4
PTK2B
EPHX2
CLU
MIR3622B
PBK
SCARA5
NUGGC
TRIM35
CHRNA2
SCARA3
ESCO2
ELP3
27
27.2
27.4
27.6
27.8
Position on chr8 (Mb)

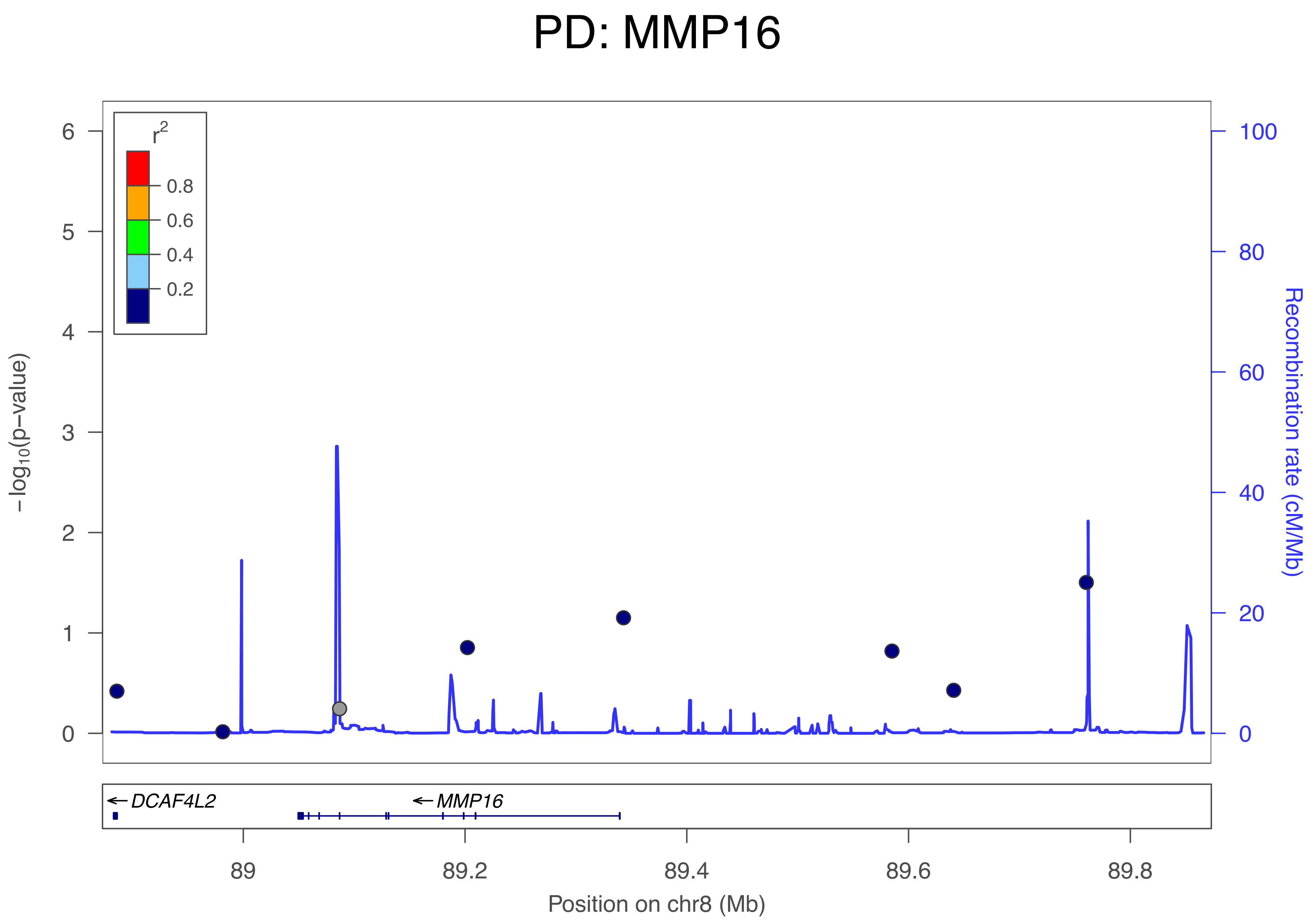

PD: MMP16
r²
0.8
0.6
0.4
0.2
−log10(p−value)
0
1
2
3
4
5
6
Recombination rate (cM/Mb)
0
20
40
60
80
100
DCAF4L2
MMP16
89
89.2
89.4
89.6
89.8
Position on chr8 (Mb)

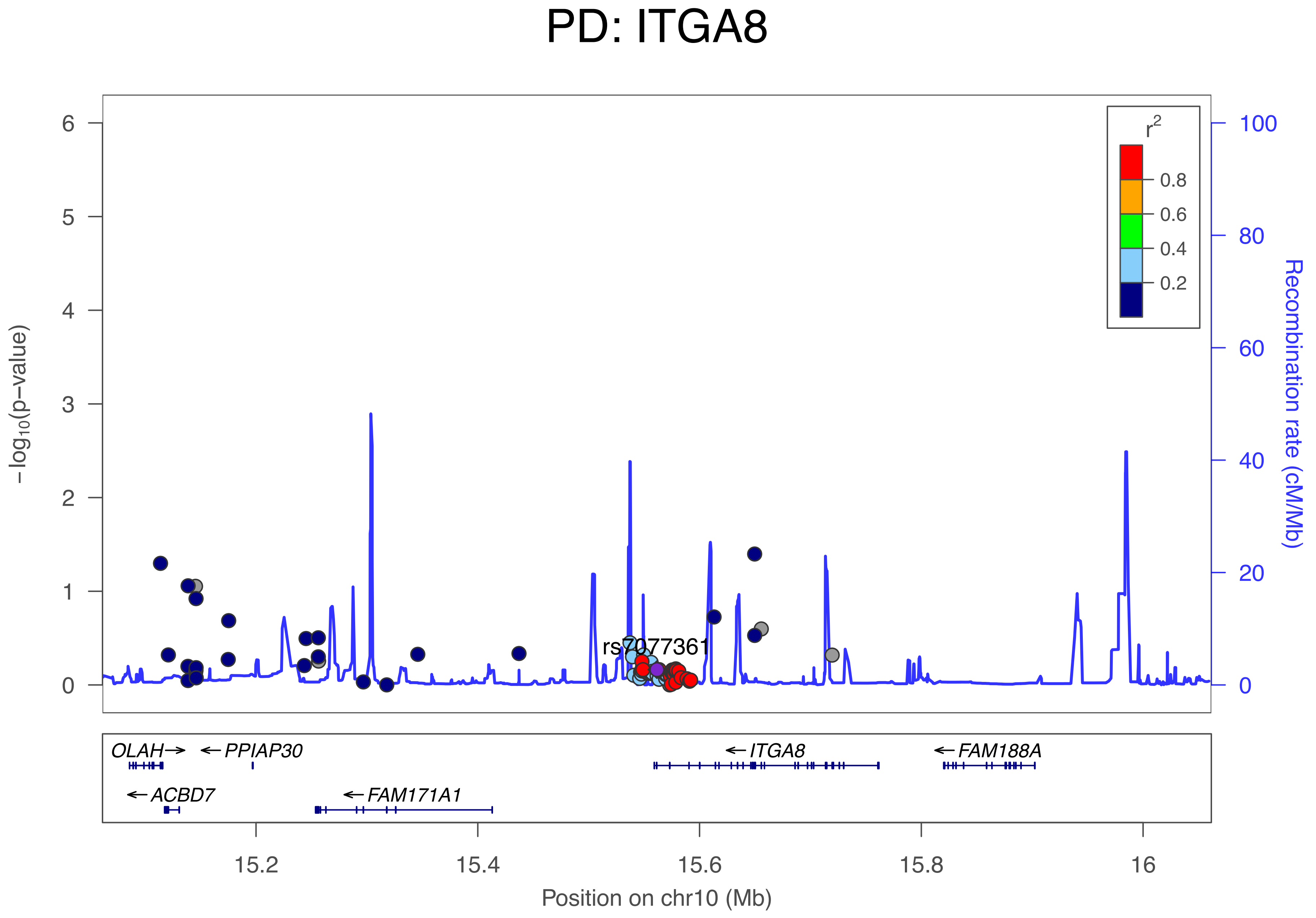

PD: ITGA8
−log10(p−value)
Recombination rate (cM/Mb)
r2
0.8
0.6
0.4
0.2
rs7077361
OLAH
PPIAP30
ITGA8
FAM188A
ACBD7
FAM171A1
15.2
15.4
15.6
15.8
16
Position on chr10 (Mb)

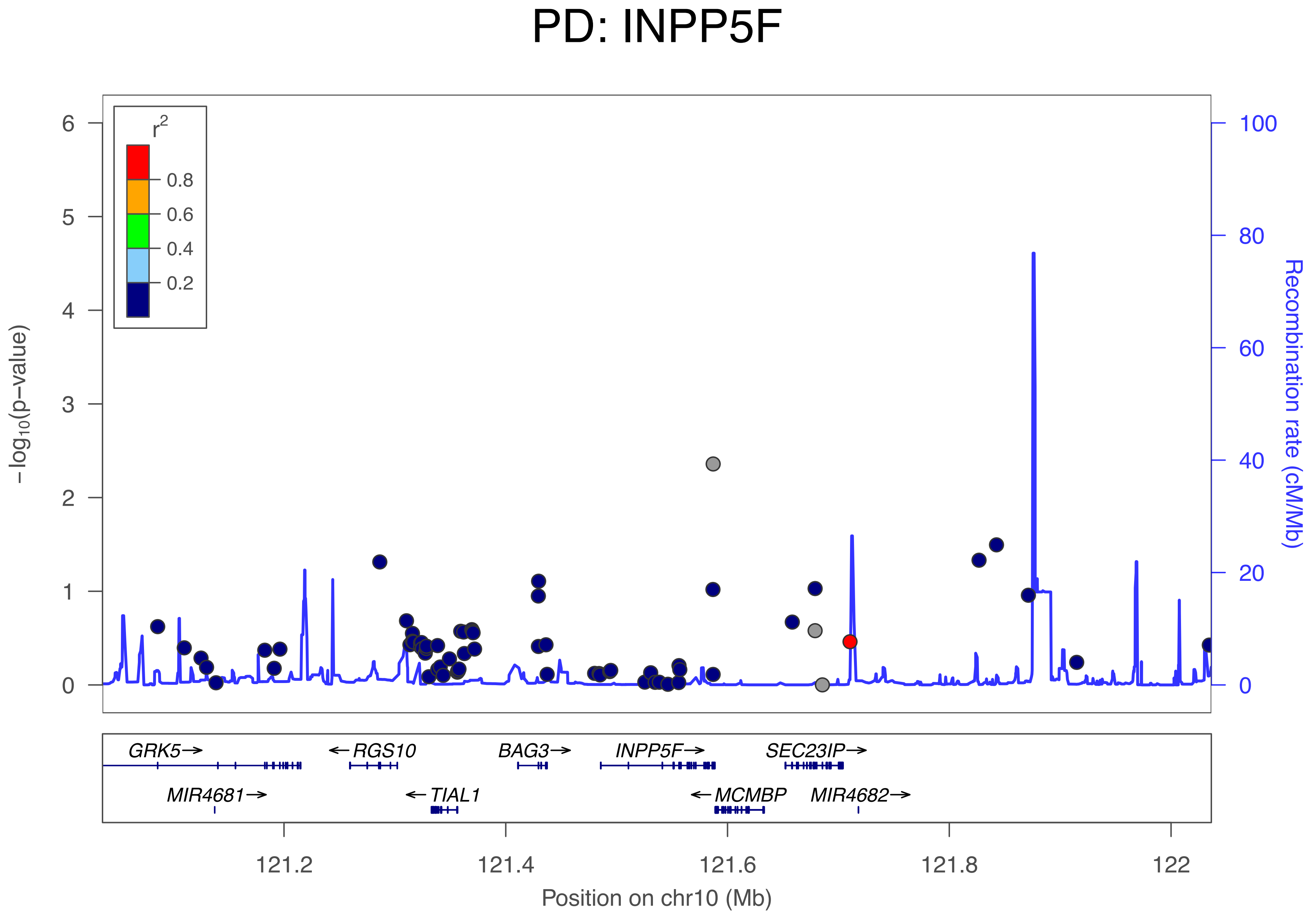

PD: INPP5F
r2
0.8
0.6
0.4
0.2
−log10(p−value)
Recombination rate (cM/Mb)
0
1
2
3
4
5
6
0
20
40
60
80
100
GRK5→
←RGS10
BAG3→
INPP5F→
SEC23IP→
MIR4681→
←TIAL1
←MCMBP
MIR4682→
121.2
121.4
121.6
121.8
122
Position on chr10 (Mb)

AD: CELF1

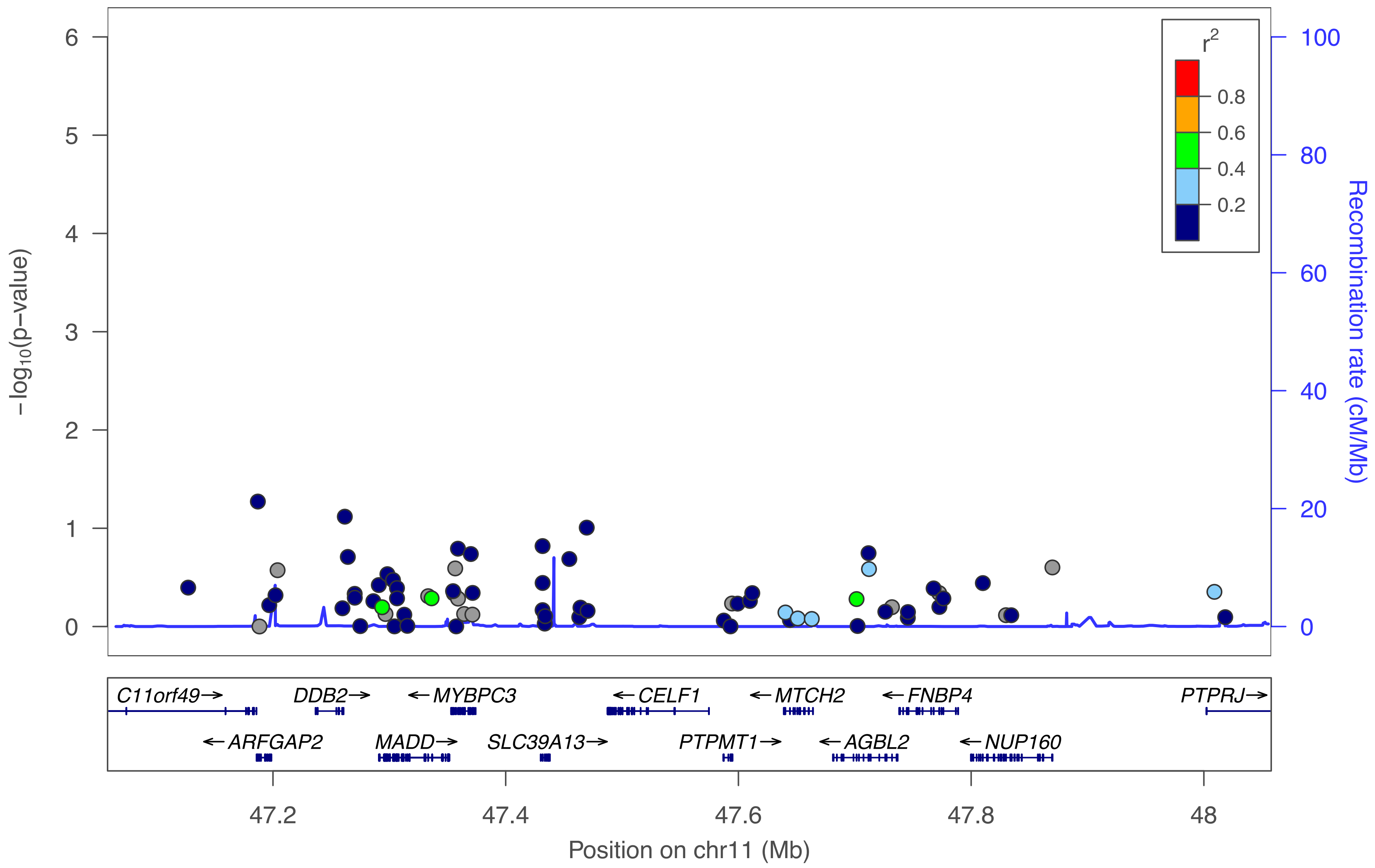

6
5
4
3
2
1
0
−log10(p−value)
100
80
60
40
20
0
Recombination rate (cM/Mb)
r2
0.8
0.6
0.4
0.2
C11orf49→
DDB2→
←MYBPC3
←CELF1
←MTCH2
←FNBP4
PTPRJ→
←ARFGAP2
MADD→
SLC39A13→
PTPMT1→
←AGBL2
←NUP160
47.2
47.4
47.6
47.8
48
Position on chr11 (Mb)

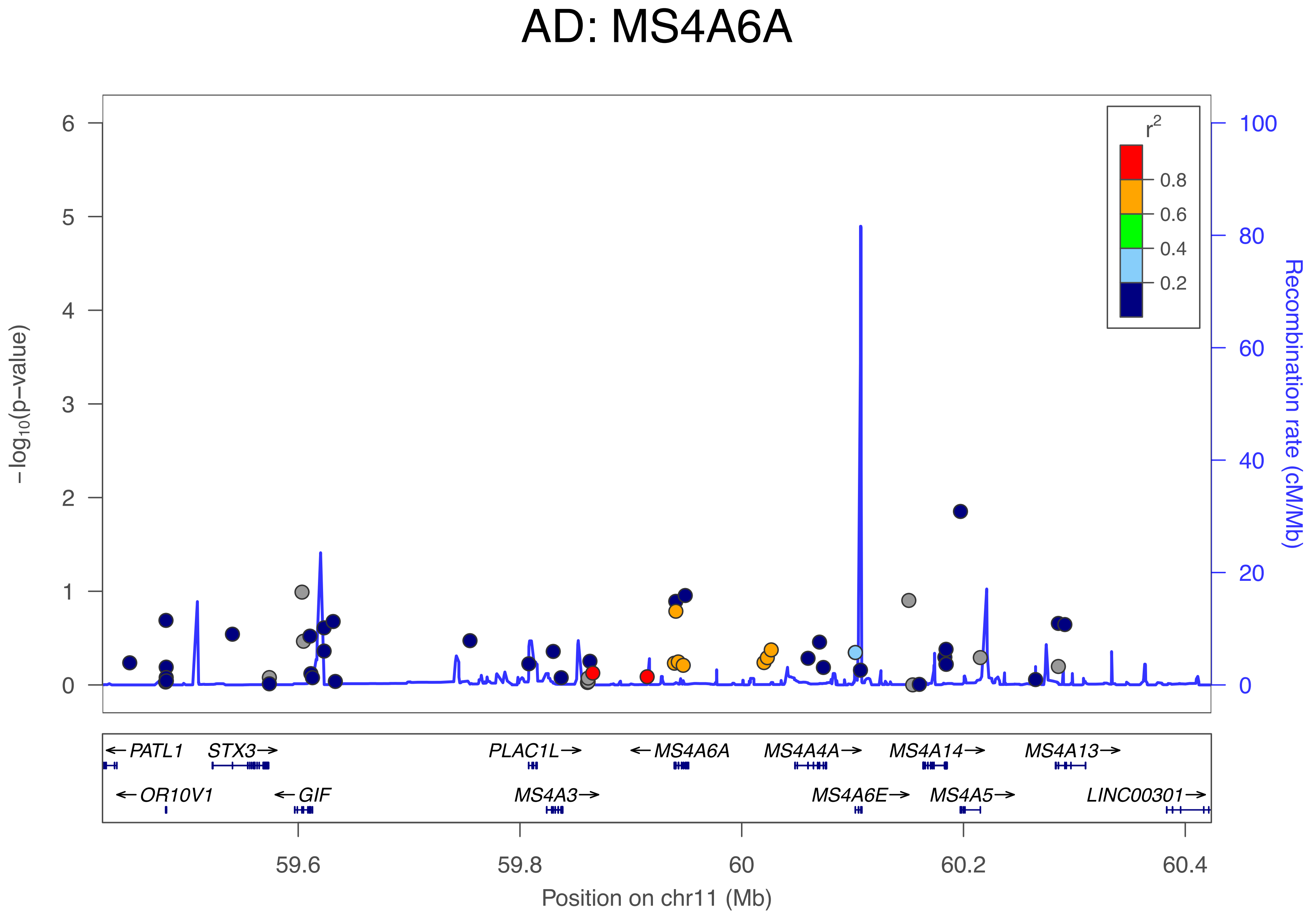

AD: MS4A6A
−log10(p−value)
0
1
2
3
4
5
6
r2
0.8
0.6
0.4
0.2
100
80
60
40
20
0
Recombination rate (cM/Mb)
← PATL1
STX3 →
PLAC1L →
← MS4A6A
MS4A4A →
MS4A14 →
MS4A13 →
← OR10V1
← GIF
MS4A3 →
MS4A6E →
MS4A5 →
LINC00301 →
59.6
59.8
60
60.2
60.4
Position on chr11 (Mb)

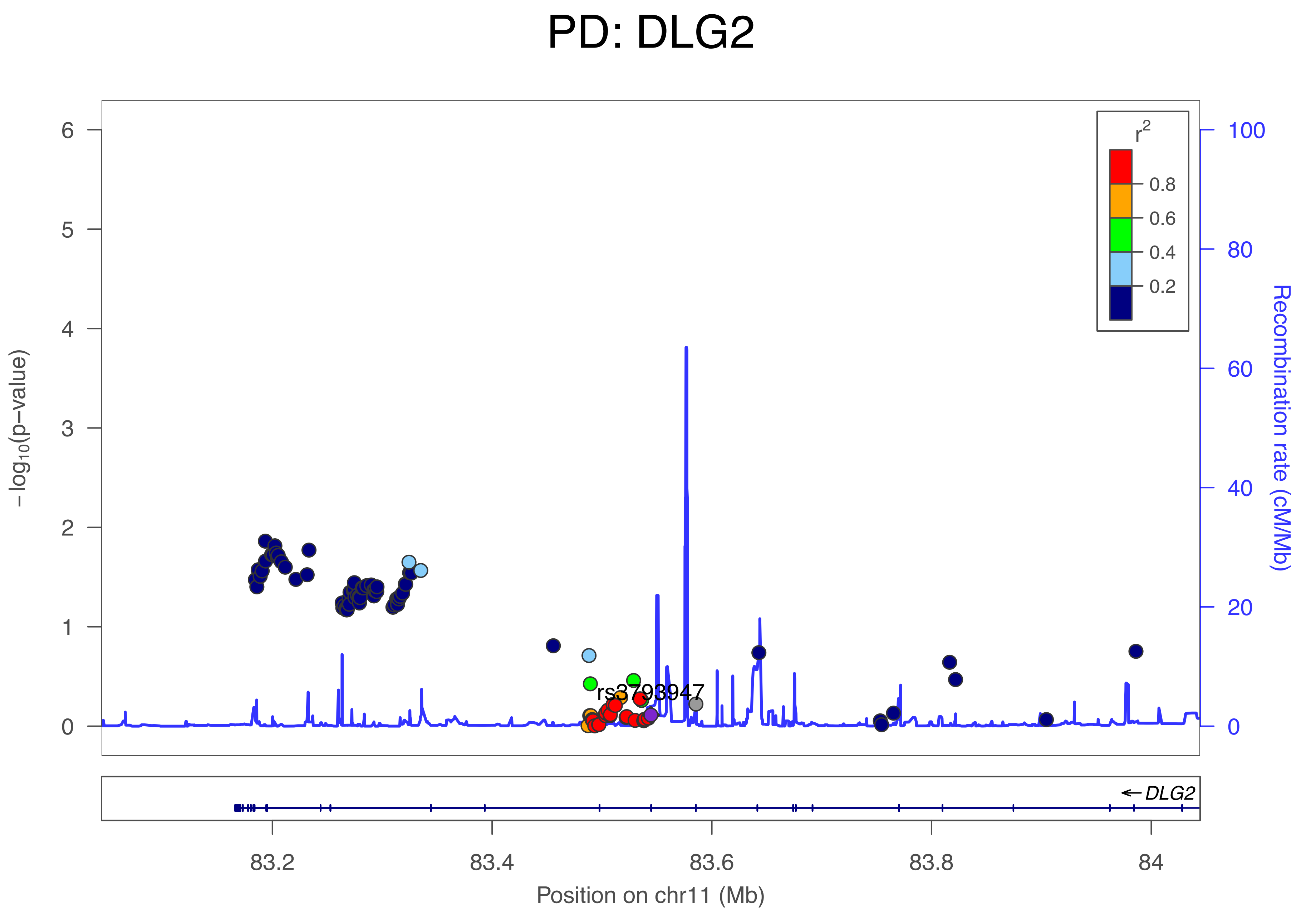

PD: DLG2
−log10(p−value)
Recombination rate (cM/Mb)
r2
0.8
0.6
0.4
0.2
rs3793947
DLG2
Position on chr11 (Mb)
83.2
83.4
83.6
83.8
84
0
1
2
3
4
5
6
0
20
40
60
80
100

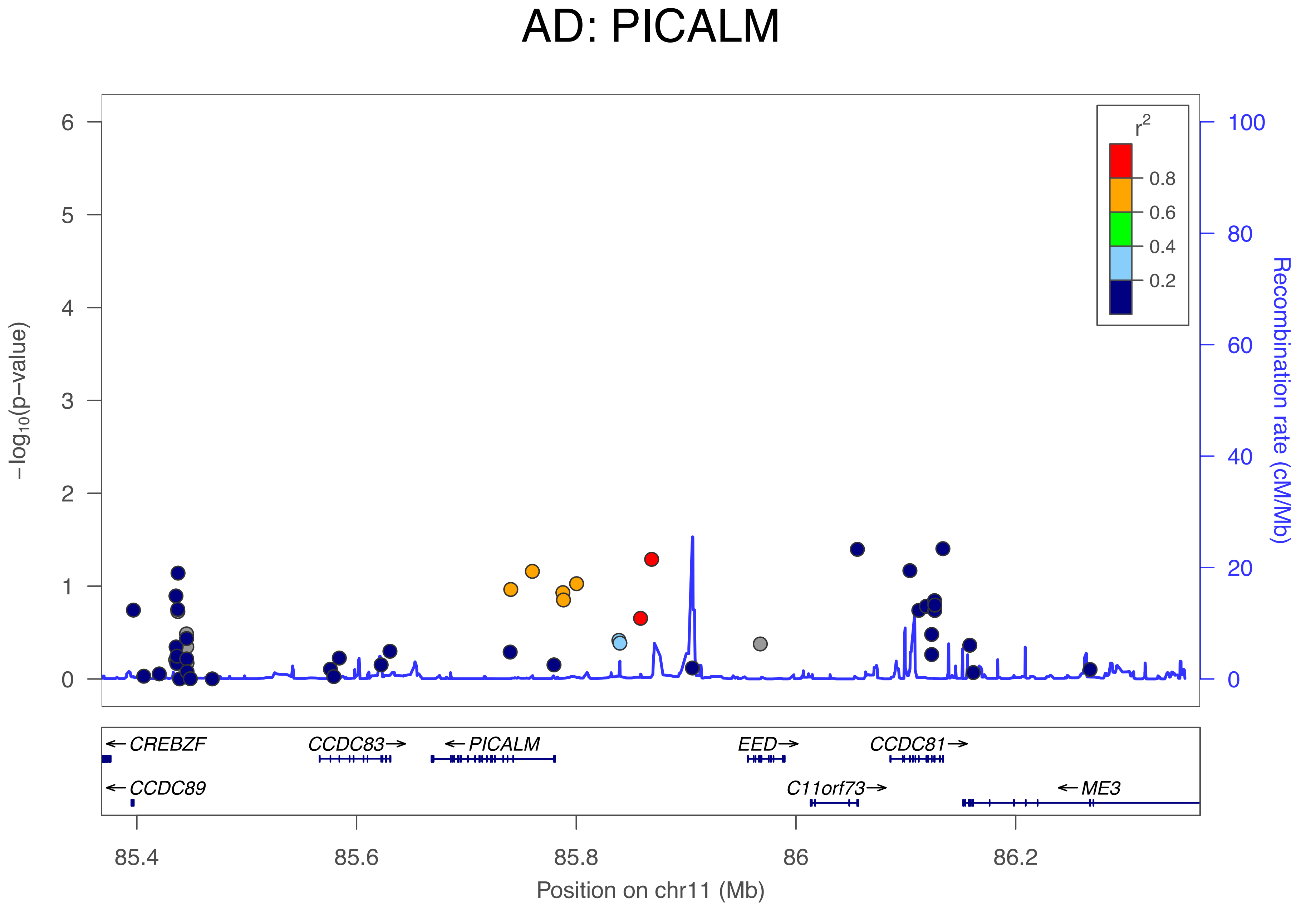

AD: PICALM
−log10(p−value)
Recombination rate (cM/Mb)
r2
0.8
0.6
0.4
0.2
0
1
2
3
4
5
6
0
20
40
60
80
100
← CREBZF
← CCDC89
CCDC83→
← PICALM
EED→
C11orf73→
CCDC81→
← ME3
85.4
85.6
85.8
86
86.2
Position on chr11 (Mb)

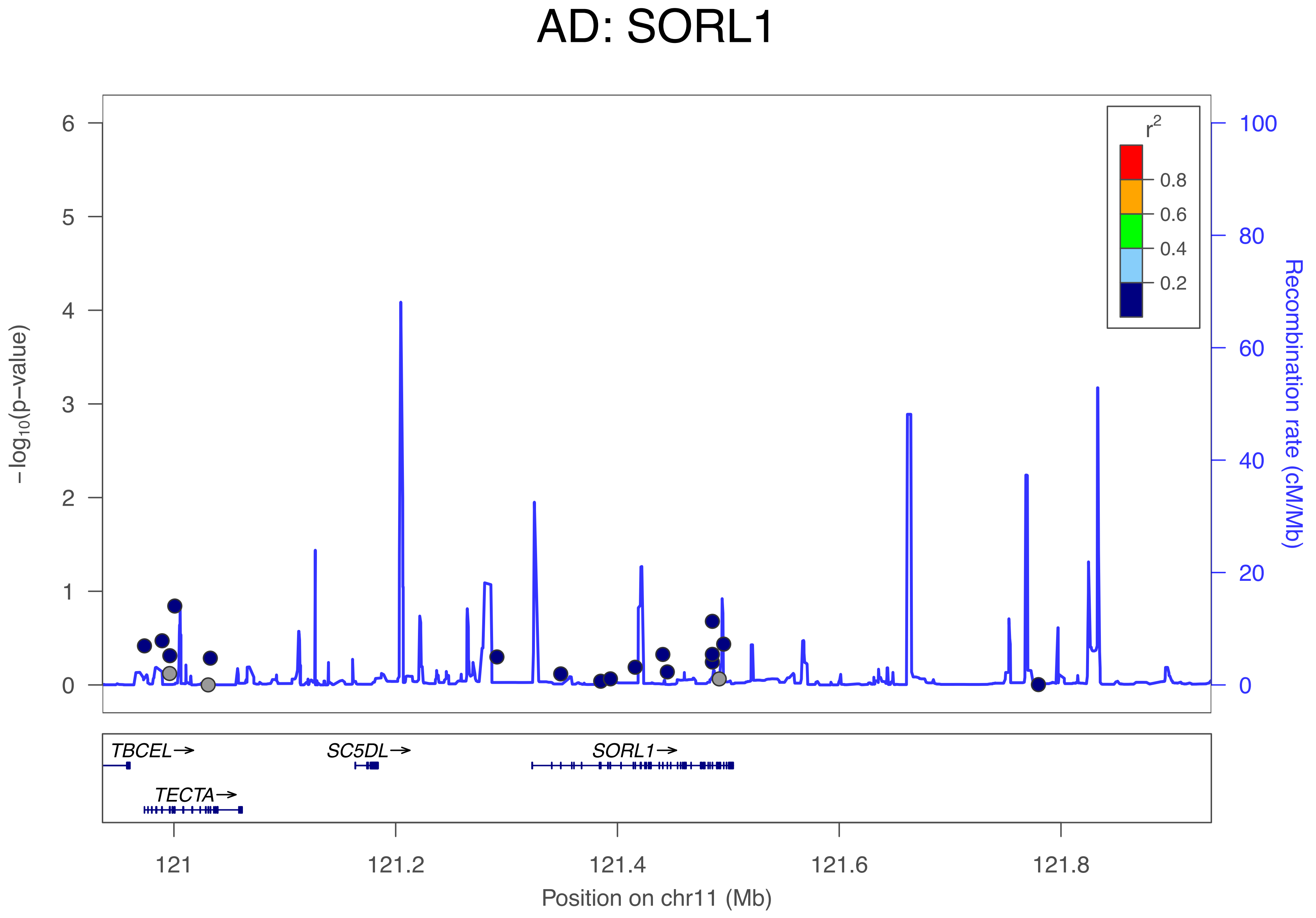

AD: SORL1
−log10(p−value)
Recombination rate (cM/Mb)
r2
0.8
0.6
0.4
0.2
TBCEL→
SC5DL→
SORL1→
TECTA→
121
121.2
121.4
121.6
121.8
Position on chr11 (Mb)

PD: MIR4697

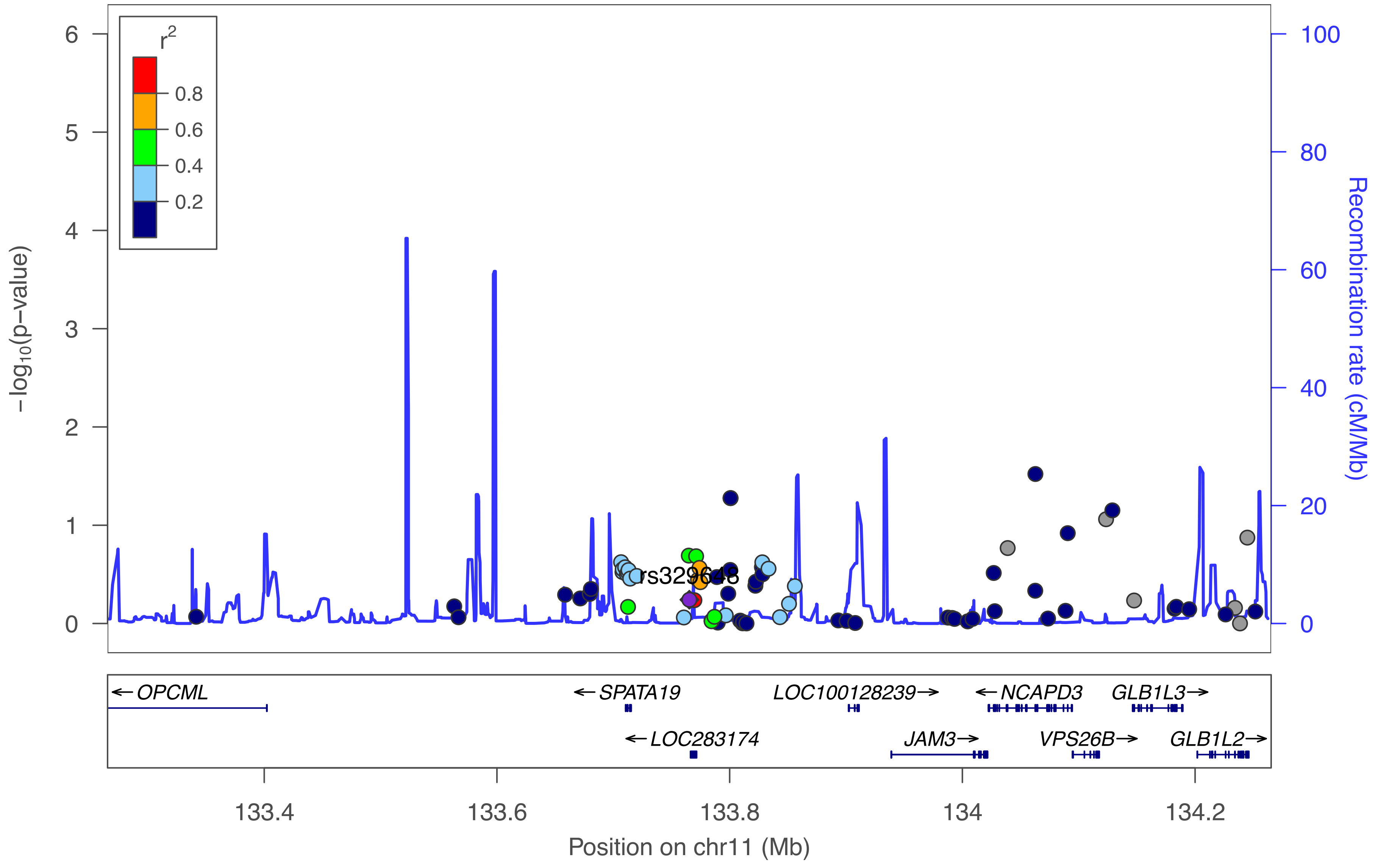

r²
0.8
0.6
0.4
0.2
−log10(p−value)
Recombination rate (cM/Mb)
rs329648
← OPCML
← SPATA19
← LOC283174
LOC100128239 →
JAM3 →
← NCAPD3
VPS26B →
GLB1L3 →
GLB1L2 →
133.4
133.6
133.8
134
134.2
Position on chr11 (Mb)

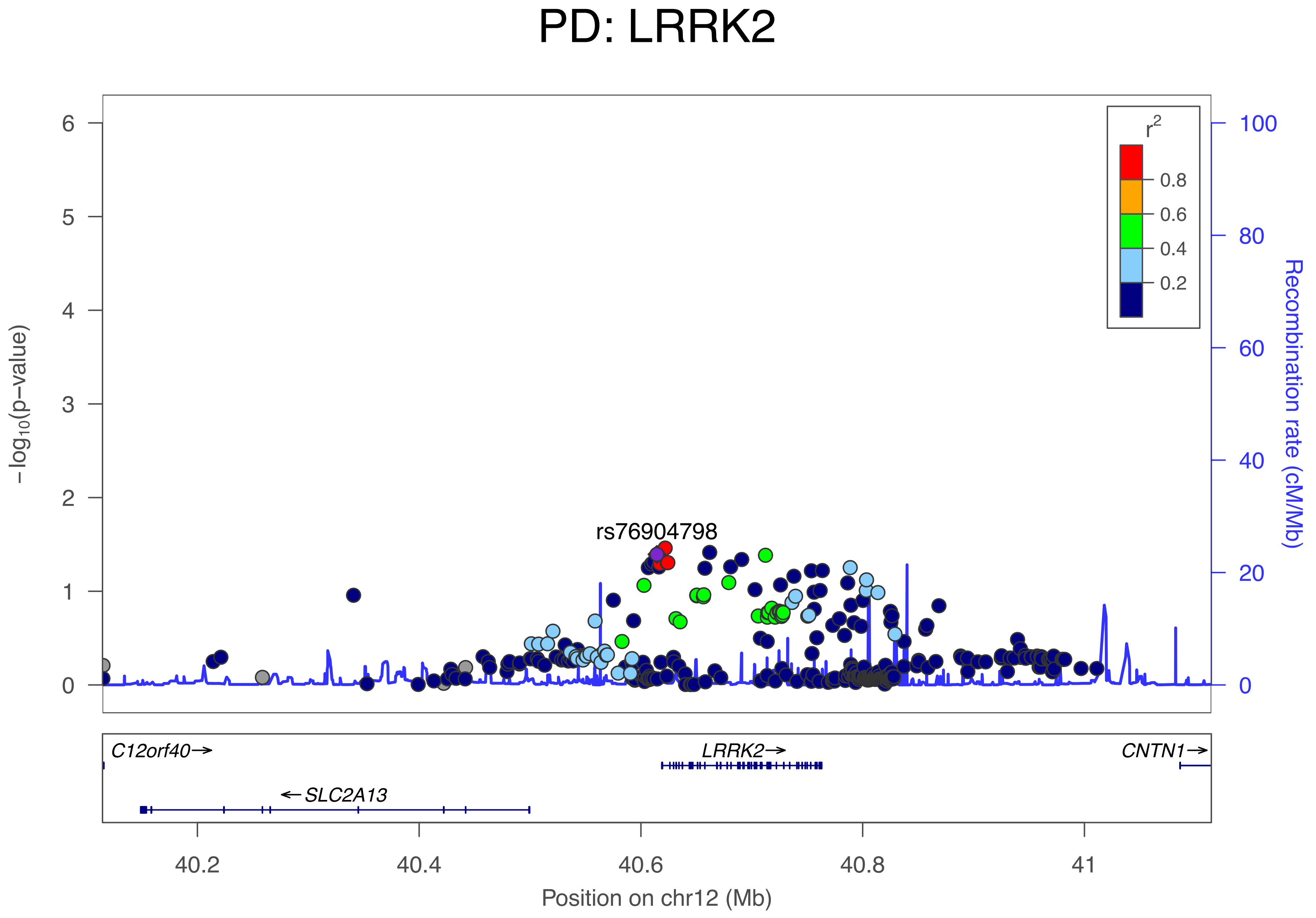

PD: LRRK2
−log10(p−value)
0
1
2
3
4
5
6
Recombination rate (cM/Mb)
0
20
40
60
80
100
r2
0.8
0.6
0.4
0.2
rs76904798
C12orf40→
←SLC2A13
LRRK2→
CNTN1→
40.2
40.4
40.6
40.8
41
Position on chr12 (Mb)

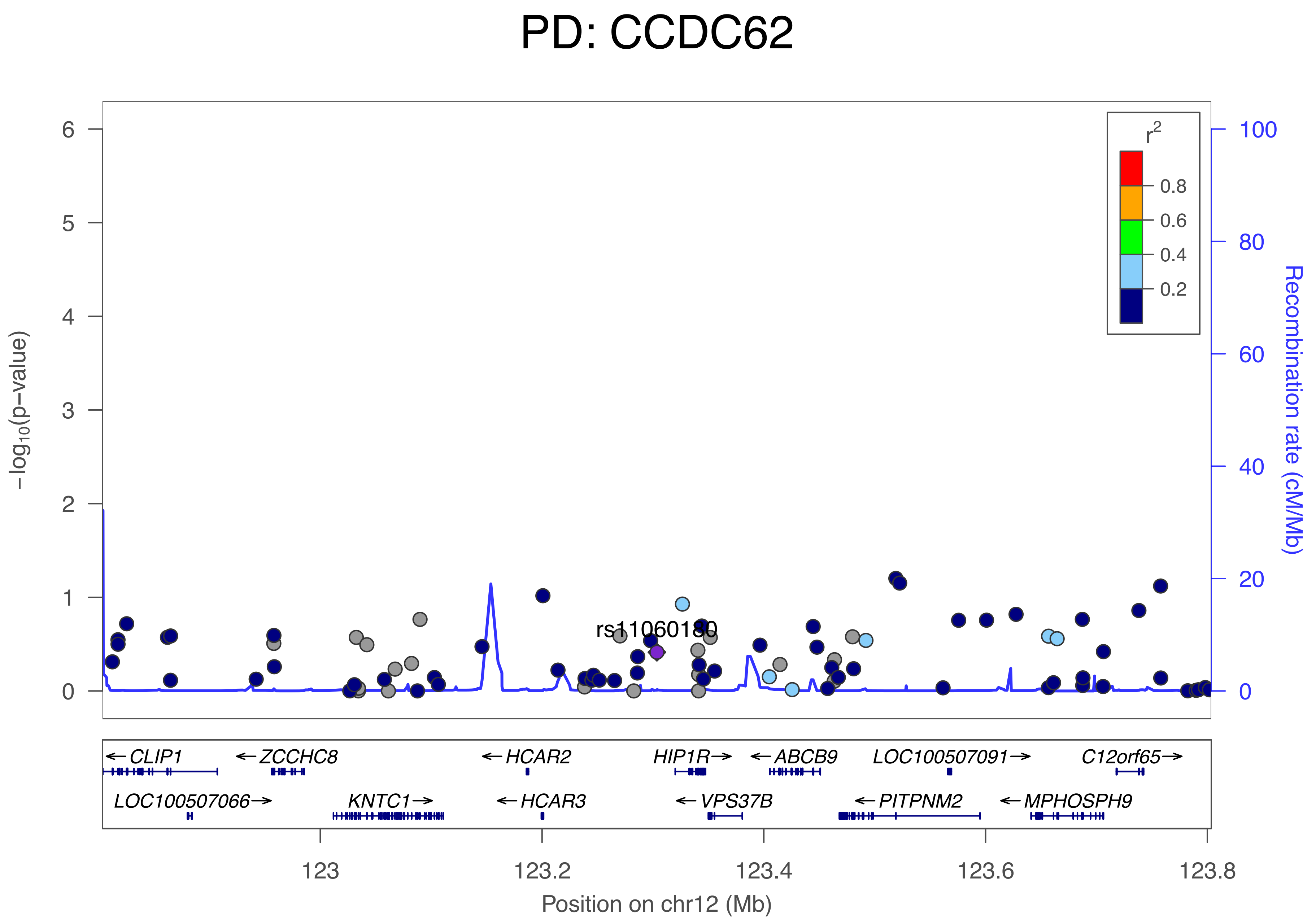

PD: CCDC62
−log10(p−value)
Recombination rate (cM/Mb)
r2
0.8
0.6
0.4
0.2
rs11060180
CLIP1
ZCCHC8
HCAR2
HIP1R
ABCB9
LOC100507091
C12orf65
LOC100507066
KNTC1
HCAR3
VPS37B
PITPNM2
MPHOSPH9
123
123.2
123.4
123.6
123.8
Position on chr12 (Mb)

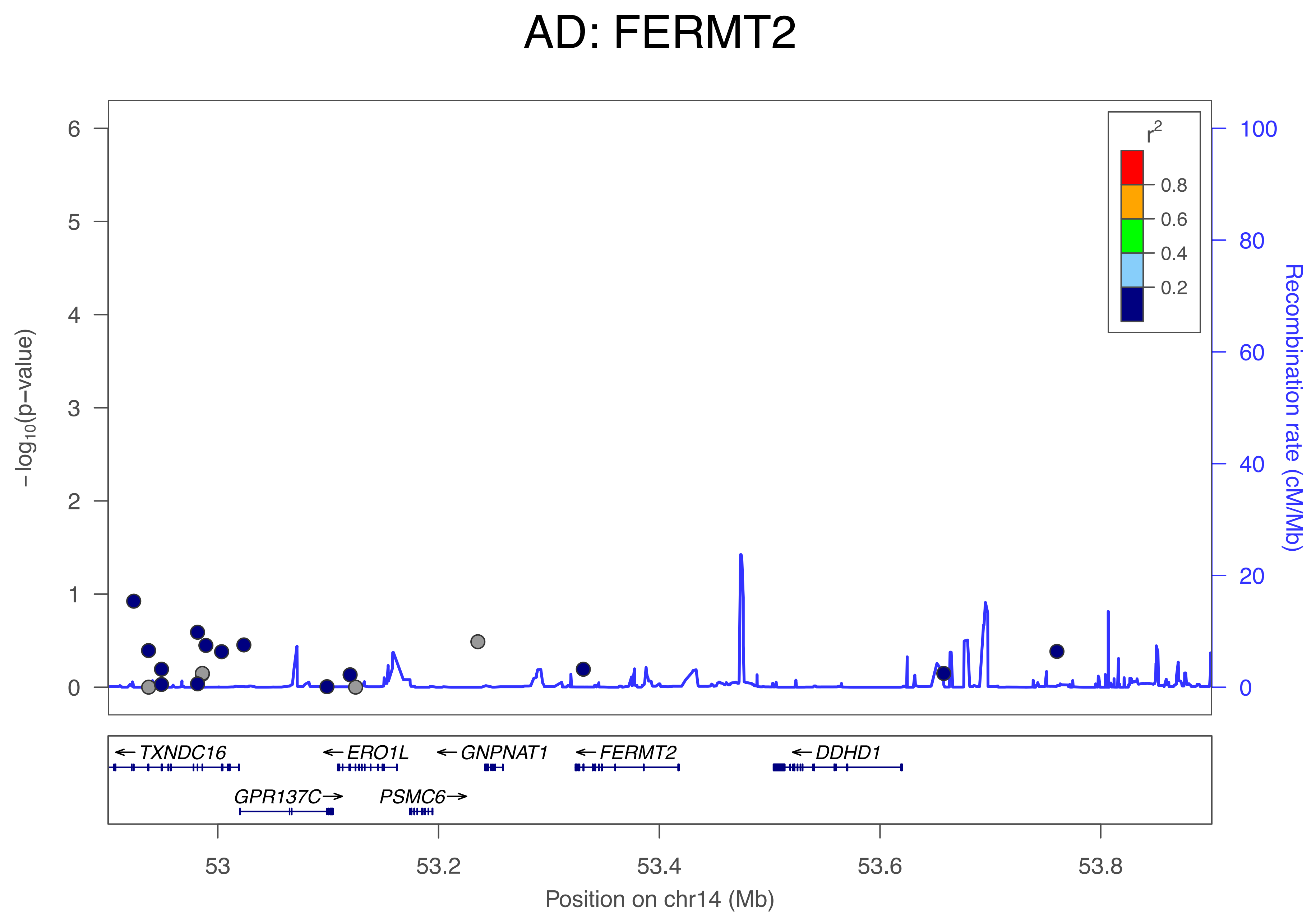
AD: FERMT2
−log10(p−value)
Recombination rate (cM/Mb)
r2
0.8
0.6
0.4
0.2
TXNDC16
ERO1L
GNPNAT1
FERMT2
DDHD1
GPR137C
PSMC6
Position on chr14 (Mb)
53
53.2
53.4
53.6
53.8

PD: GCH1

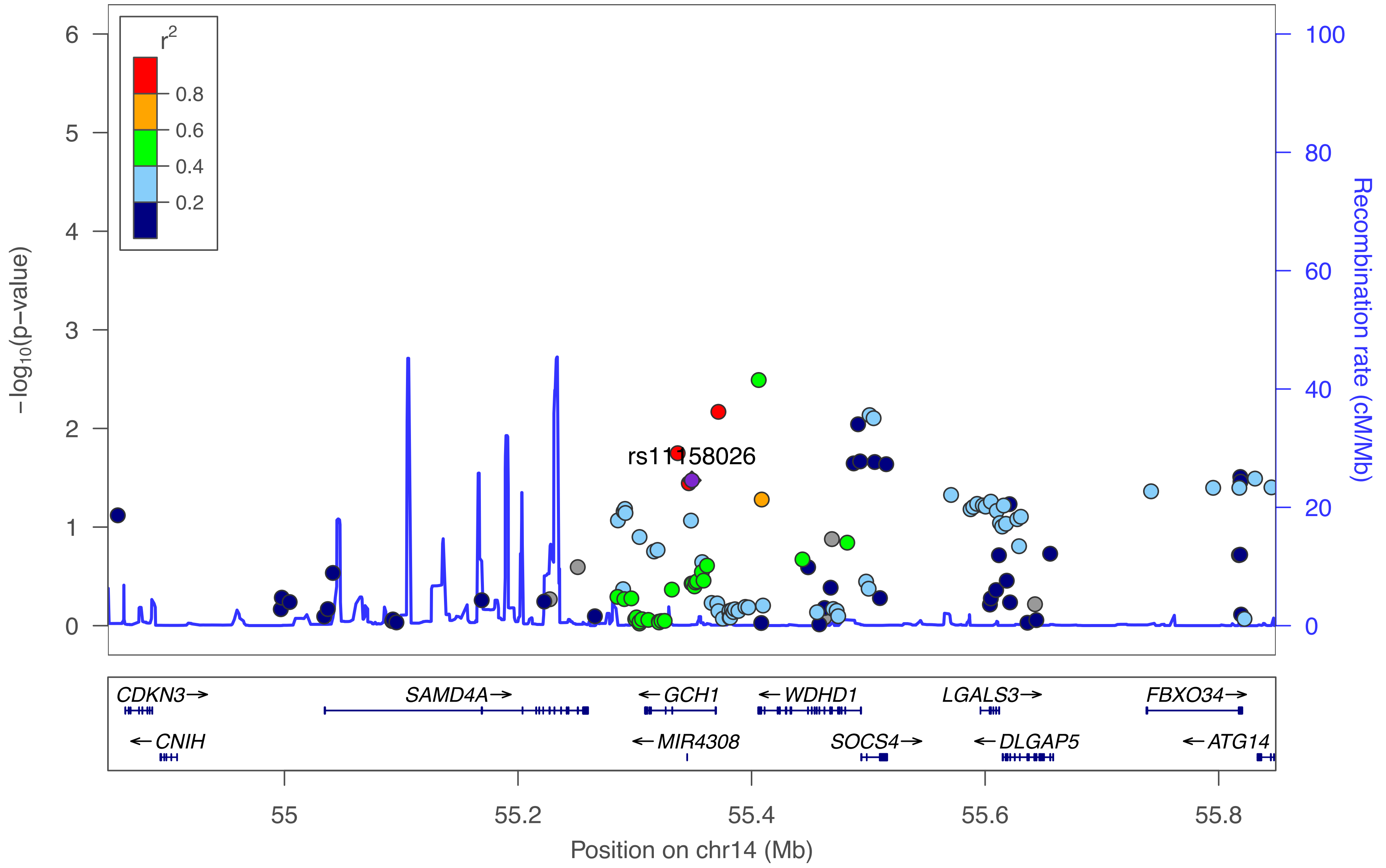

r2
0.8
0.6
0.4
0.2
rs11158026
6
5
4
3
2
1
0
-log10(p-value)
100
80
60
40
20
0
Recombination rate (cM/Mb)
CDKN3→
SAMD4A→
← GCH1
← WDHD1
LGALS3→
FBXO34→
← CNIH
← MIR4308
SOCS4→
← DLGAP5
← ATG14
55
55.2
55.4
55.6
55.8
Position on chr14 (Mb)

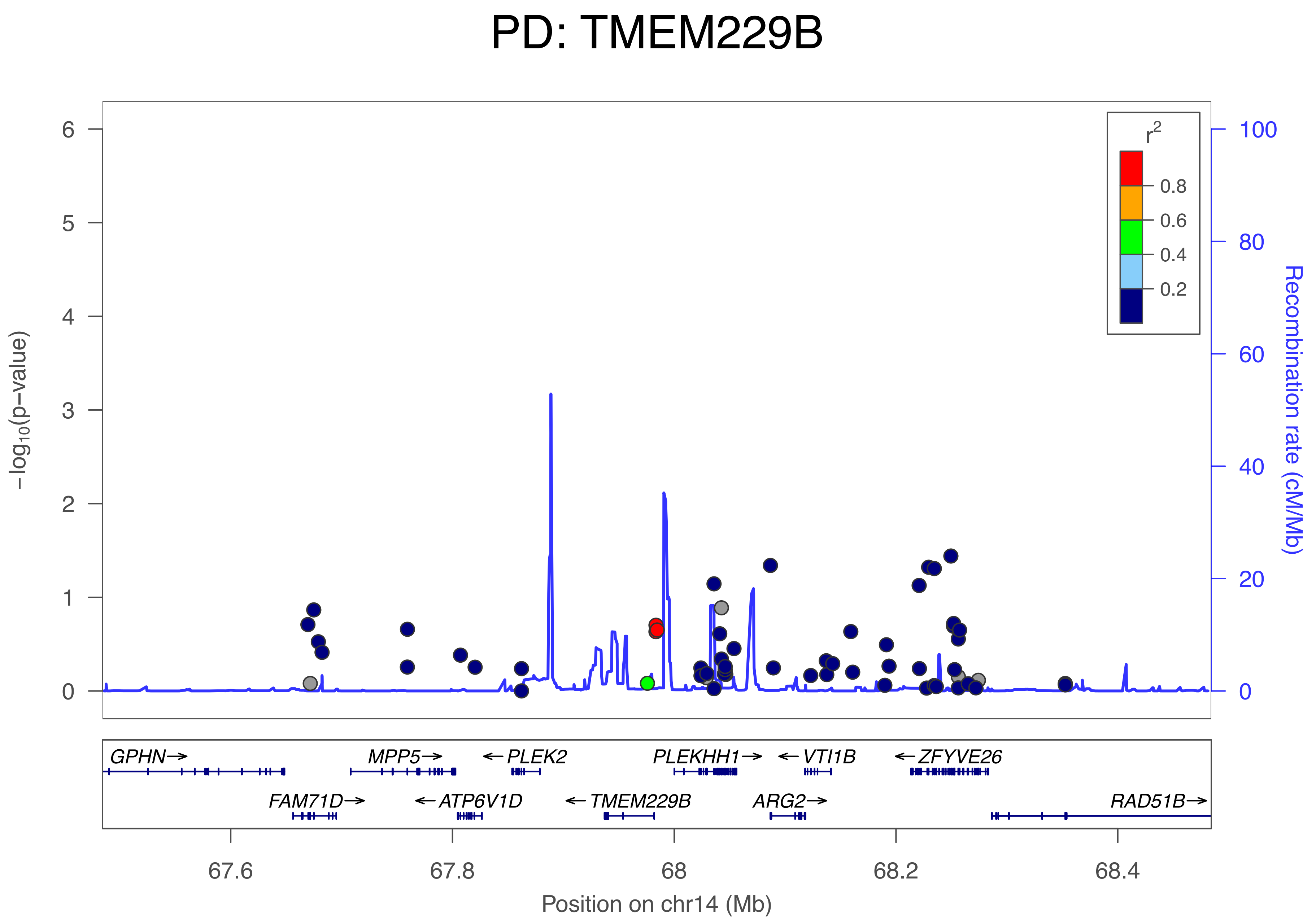
PD: TMEM229B
−log10(p−value)
Recombination rate (cM/Mb)
r2
0.8
0.6
0.4
0.2
GPHN→
MPP5→
←PLEK2
PLEKHH1→
←VTI1B
←ZFYVE26
FAM71D→
←ATP6V1D
←TMEM229B
ARG2→
RAD51B→
67.6
67.8
68
68.2
68.4
Position on chr14 (Mb)

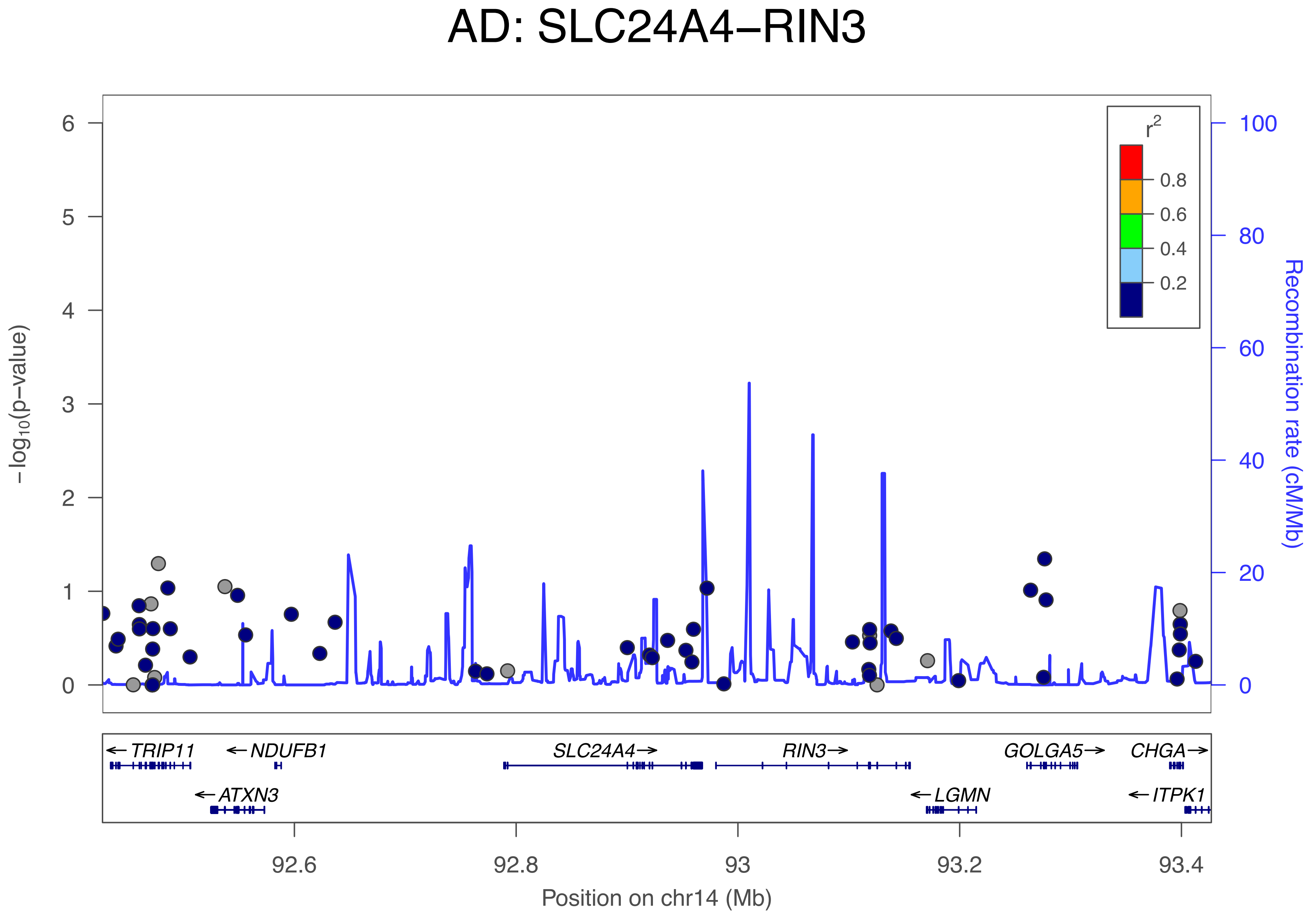

AD: SLC24A4−RIN3
−log10(p−value)
0
1
2
3
4
5
6
Recombination rate (cM/Mb)
0
20
40
60
80
100
r2
0.8
0.6
0.4
0.2
TRIP11
ATXN3
NDUFB1
SLC24A4
RIN3
LGMN
GOLGA5
CHGA
ITPK1
92.6
92.8
93
93.2
93.4
Position on chr14 (Mb)

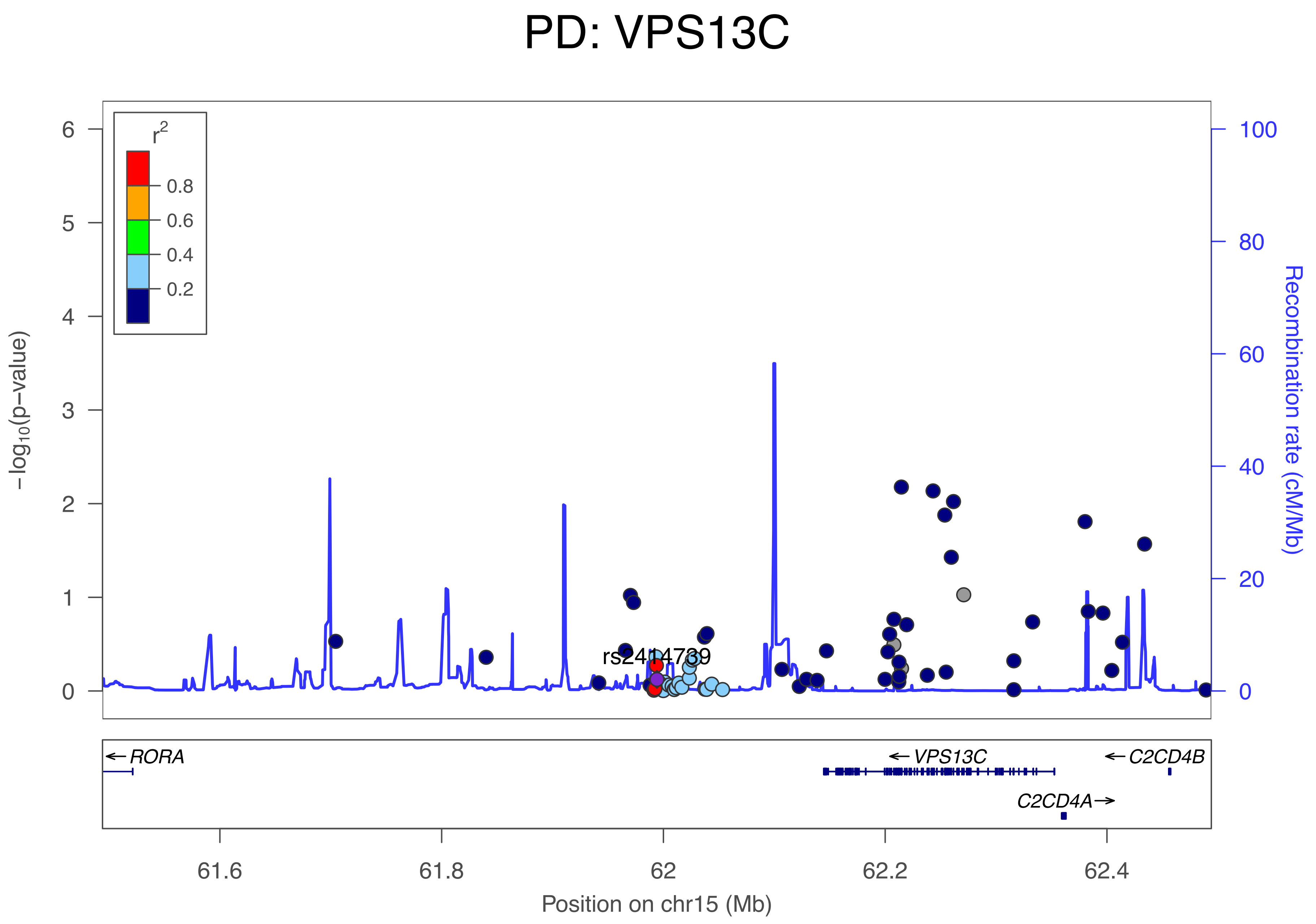

PD: VPS13C
r2
0.8
0.6
0.4
0.2
rs2414739
−log10(p−value)
Recombination rate (cM/Mb)
RORA
VPS13C
C2CD4B
C2CD4A
61.6
61.8
62
62.2
62.4
Position on chr15 (Mb)

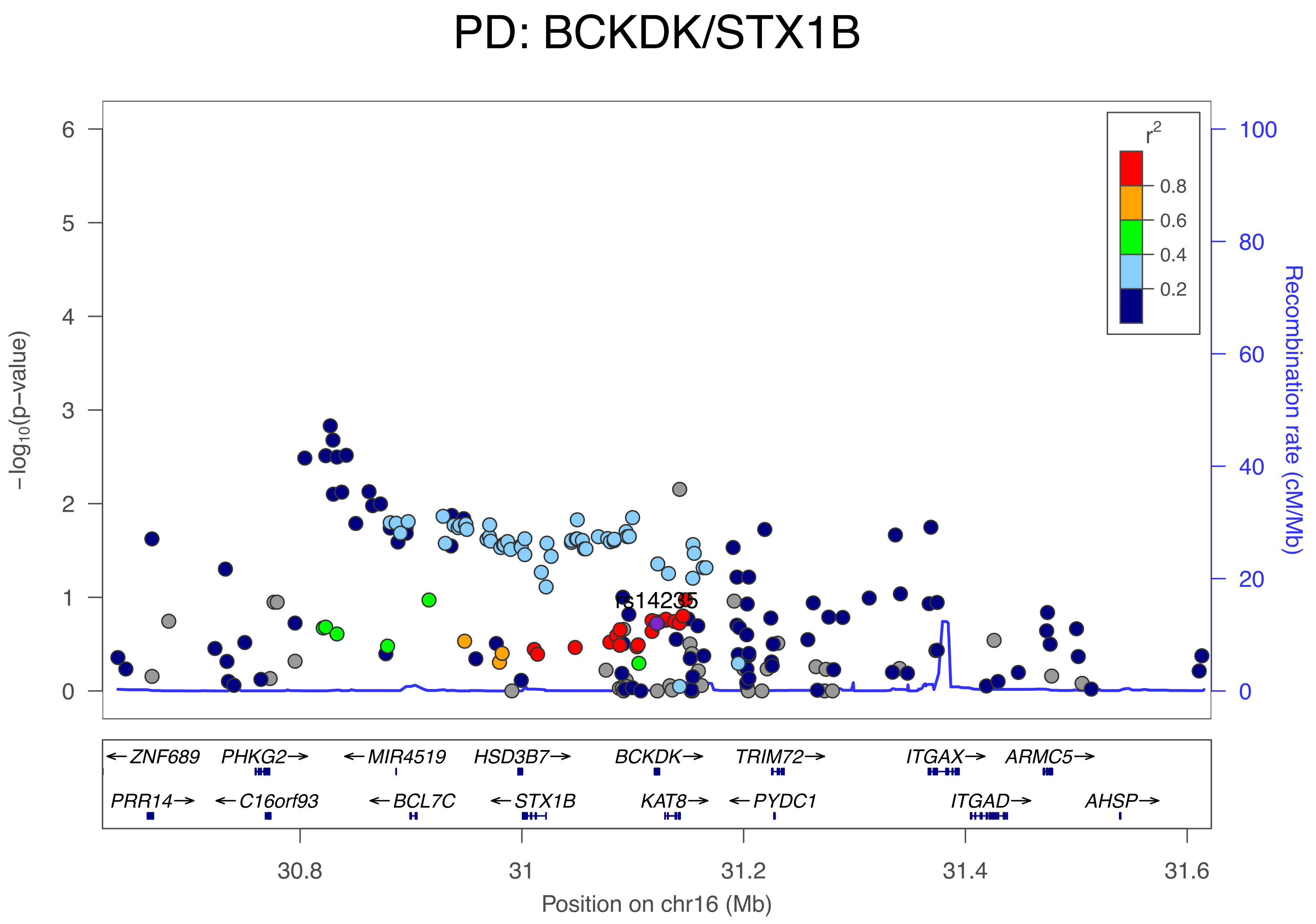

PD: BCKDK/STX1B
-log10(p-value)
Recombination rate (cM/Mb)
r2
0.8
0.6
0.4
0.2
rs14235
ZNF689
PHKG2
MIR4519
HSD3B7
BCKDK
TRIM72
ITGAX
ARMC5
PRR14
C16orf93
BCL7C
STX1B
KAT8
PYDC1
ITGAD
AHSP
30.8
31
31.2
31.4
31.6
Position on chr16 (Mb)

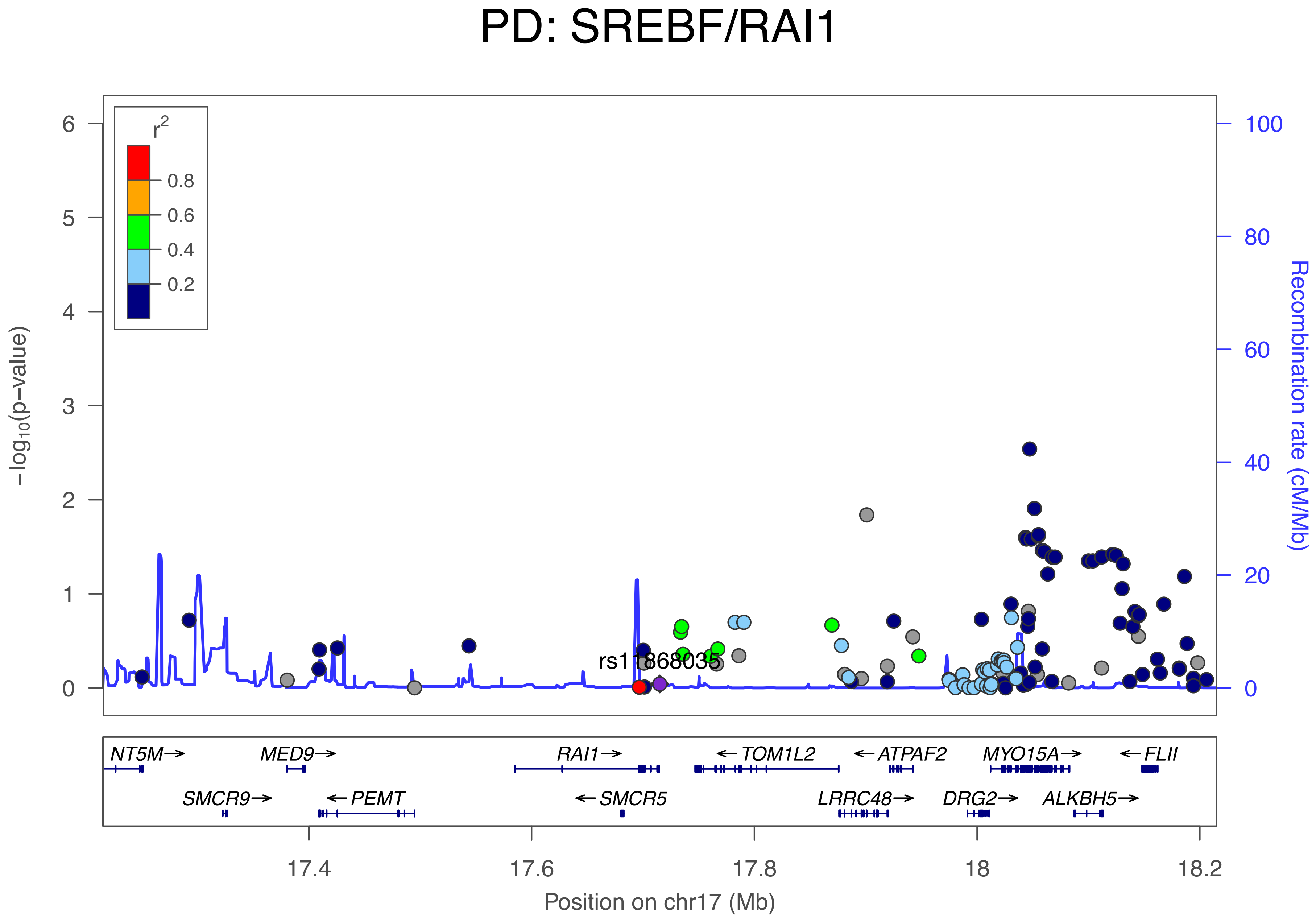

PD: SREBF/RAI1
r²
0.8
0.6
0.4
0.2
−log10(p−value)
Recombination rate (cM/Mb)
rs11868035
NT5M
MED9
RAI1
TOM1L2
ATPAF2
MYO15A
FLII
SMCR9
PEMT
SMCR5
LRRC48
DRG2
ALKBH5
17.4
17.6
17.8
18
18.2
Position on chr17 (Mb)

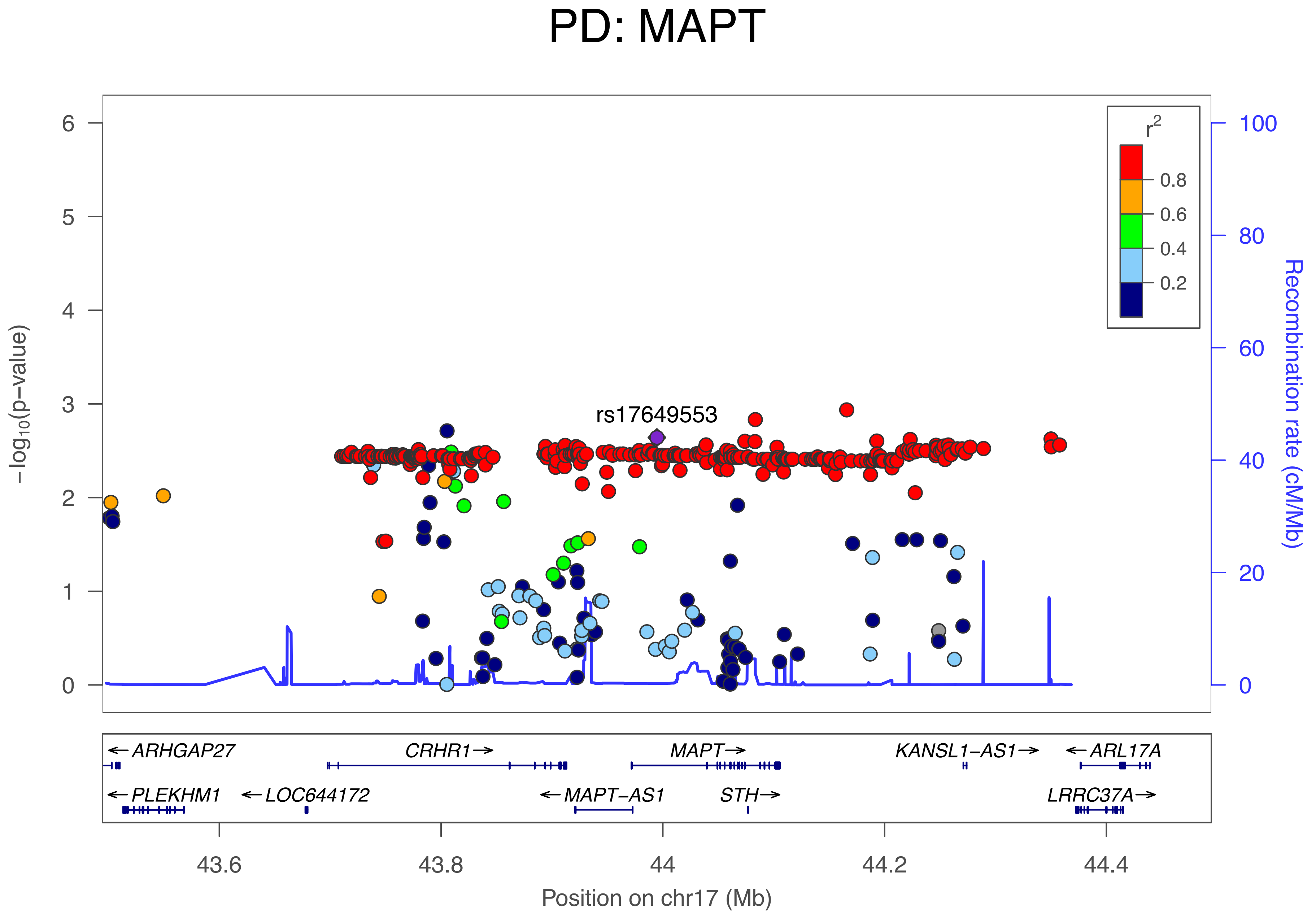
PD: MAPT
rs17649553
r2
0.8
0.6
0.4
0.2
−log10(p−value)
Recombination rate (cM/Mb)
0
1
2
3
4
5
6
0
20
40
60
80
100
←ARHGAP27
CRHR1→
MAPT→
KANSL1−AS1→
←ARL17A
←PLEKHM1
←LOC644172
←MAPT−AS1
STH→
LRRC37A→
43.6
43.8
44
44.2
44.4
Position on chr17 (Mb)

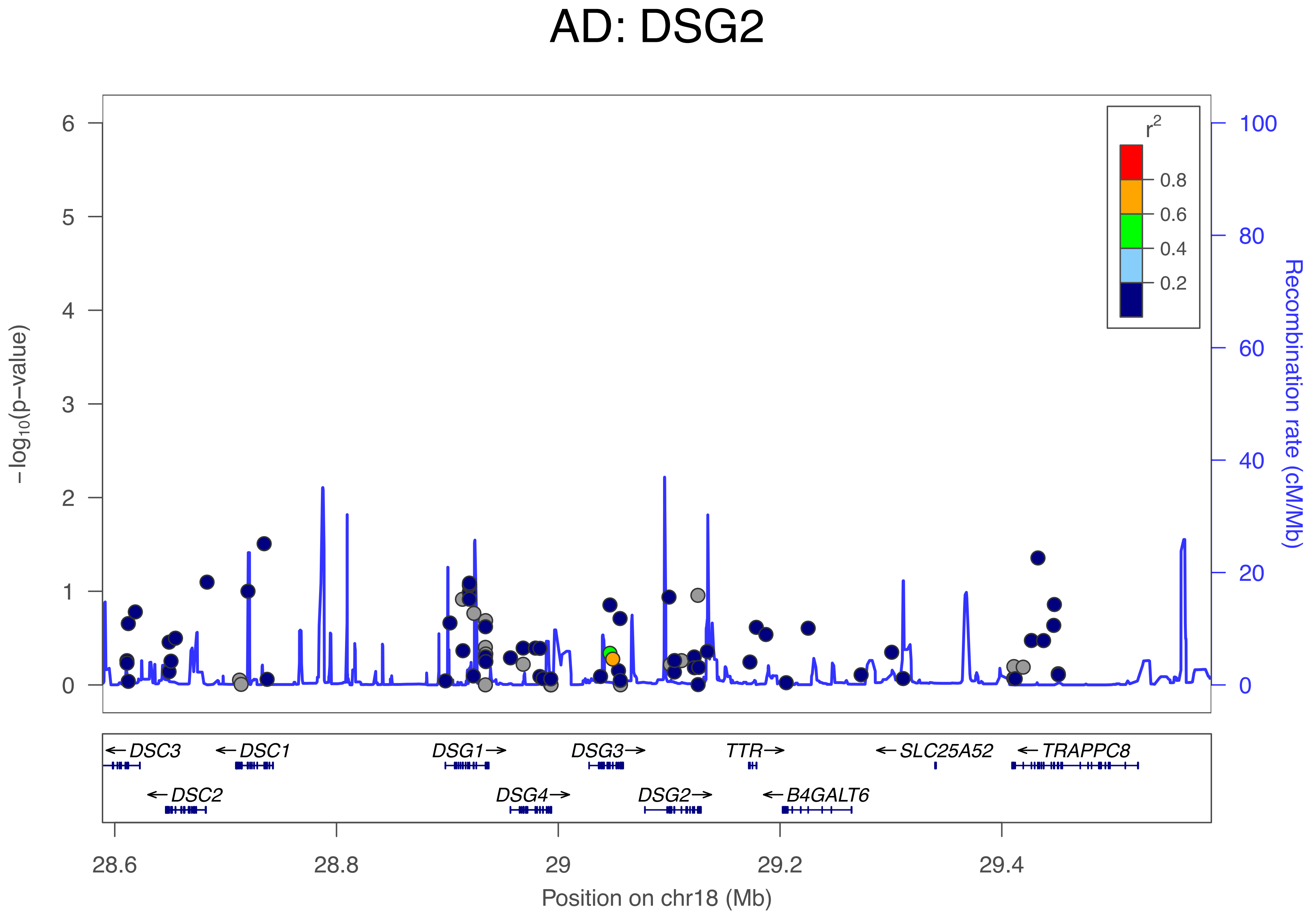

AD: DSG2
−log10(p−value)
0
1
2
3
4
5
6
r2
0.8
0.6
0.4
0.2
100
80
60
40
20
0
Recombination rate (cM/Mb)
DSC3
DSC1
DSG1
DSG3
TTR
SLC25A52
TRAPPC8
DSC2
DSG4
DSG2
B4GALT6
28.6
28.8
29
29.2
29.4
Position on chr18 (Mb)

PD: RIT2

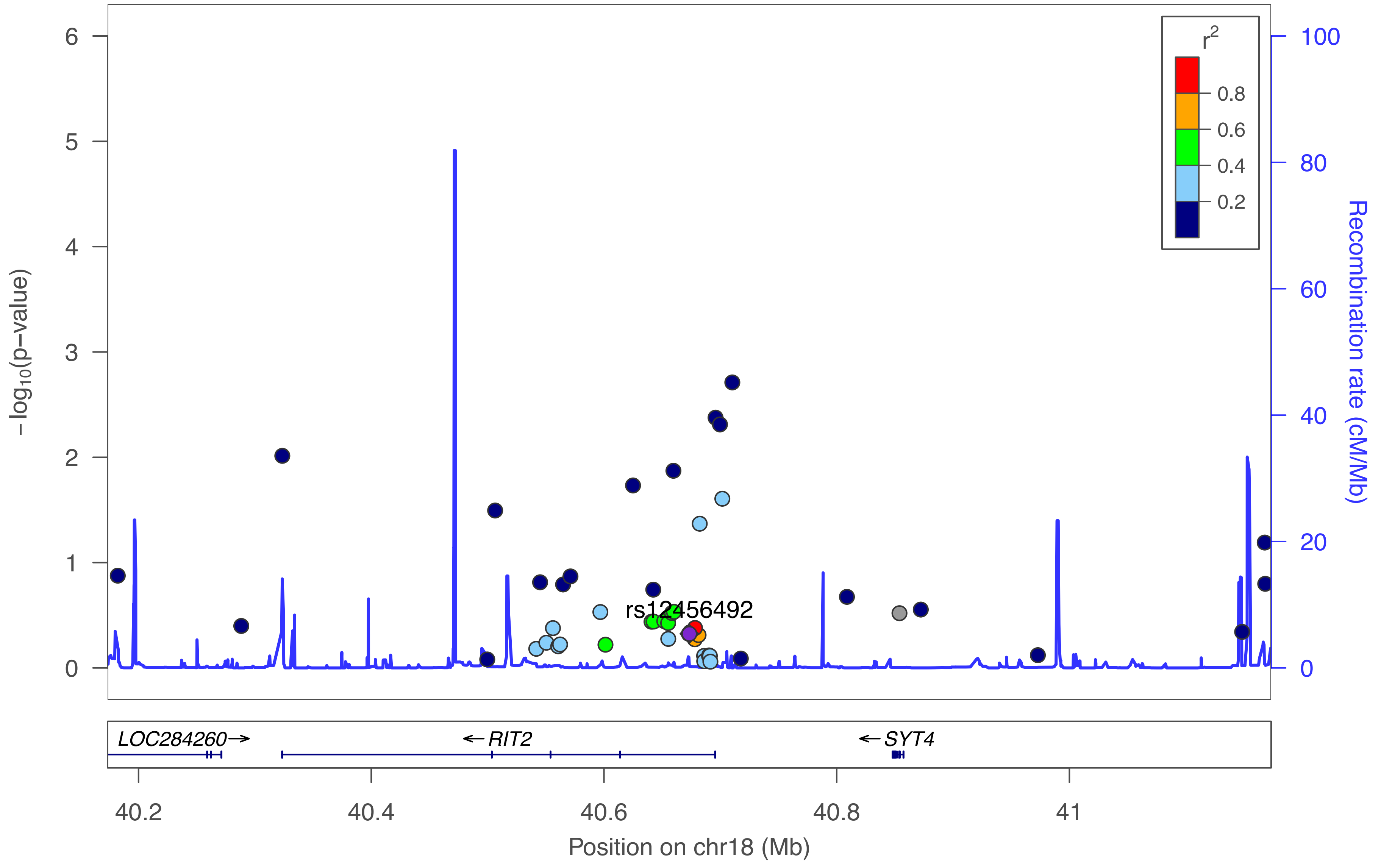

rs12456492
r2
0.8
0.6
0.4
0.2
6
5
4
3
2
1
0
−log10(p−value)
100
80
60
40
20
0
Recombination rate (cM/Mb)
LOC284260→
← RIT2
← SYT4
40.2
40.4
40.6
40.8
41
Position on chr18 (Mb)

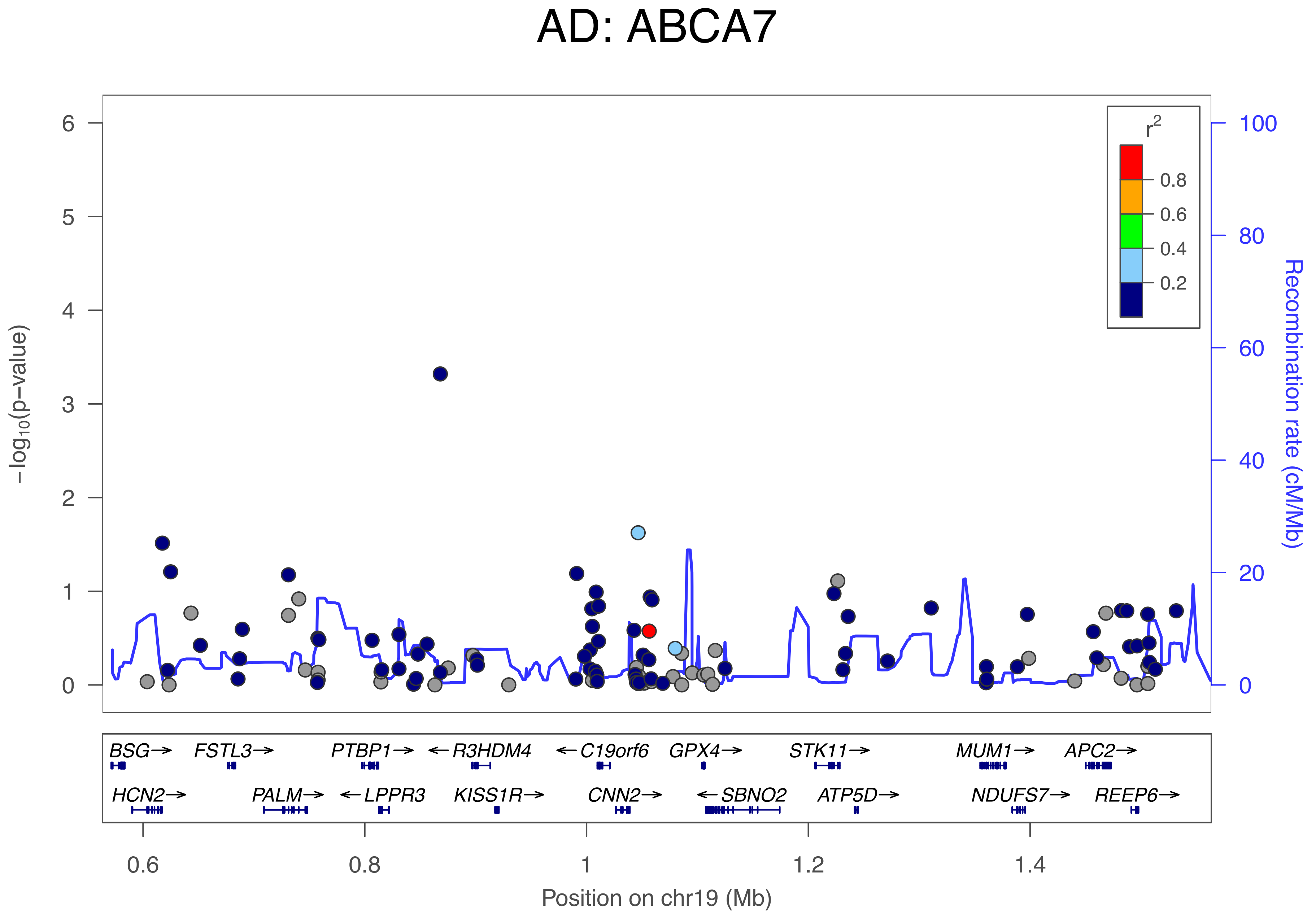
AD: ABCA7
r^2
0.8
0.6
0.4
0.2
−log10(p−value)
Recombination rate (cM/Mb)
0 1 2 3 4 5 6
0 20 40 60 80 100
BSG→ FSTL3→ PTBP1→ ←R3HDM4 ←C19orf6 GPX4→ STK11→ MUM1→ APC2→
HCN2→ PALM→ ←LPPR3 KISS1R→ CNN2→ ←SBNO2 ATP5D→ NDUFS7→ REEP6→
0.6 0.8 1 1.2 1.4
Position on chr19 (Mb)

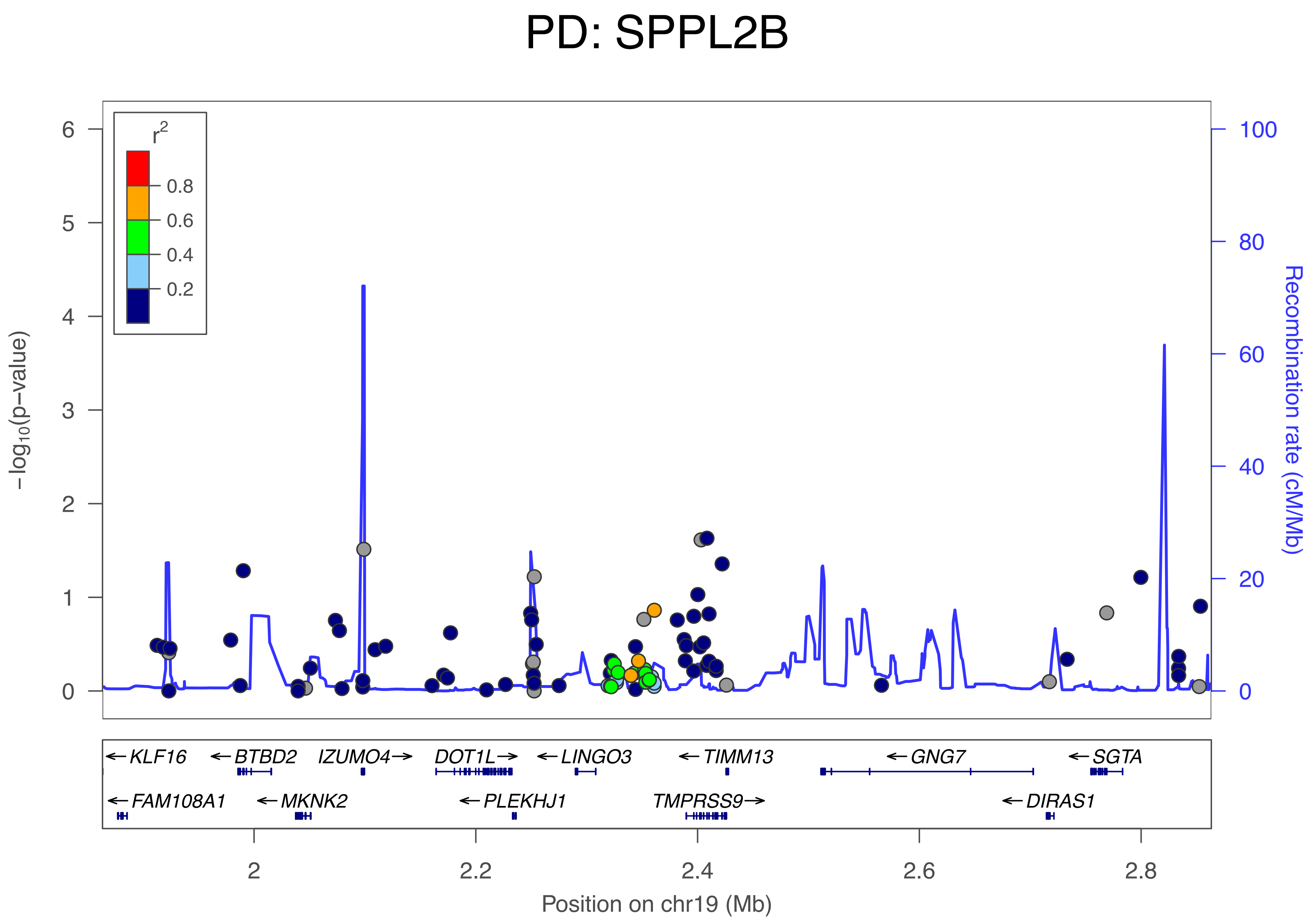

PD: SPPL2B
r2
0.8
0.6
0.4
0.2
−log10(p−value)
Recombination rate (cM/Mb)
KLF16
BTBD2
IZUMO4
DOT1L
LINGO3
TIMM13
GNG7
SGTA
FAM108A1
MKNK2
PLEKHJ1
TMPRSS9
DIRAS1
Position on chr19 (Mb)

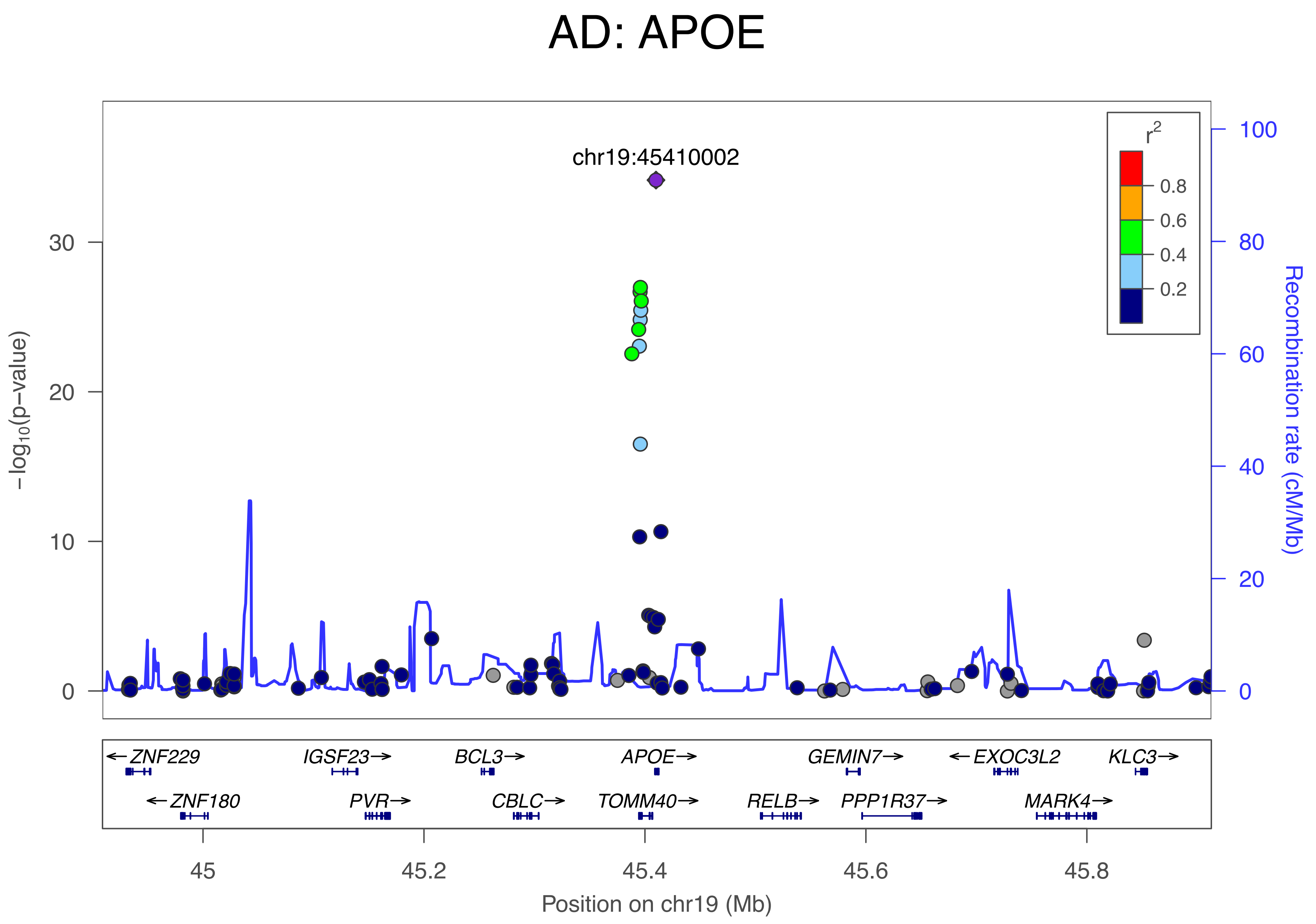
AD: APOE
chr19:45410002
r²
0.8
0.6
0.4
0.2
−log10(p−value)
0
10
20
30
Recombination rate (cM/Mb)
0
20
40
60
80
100
ZNF229
ZNF180
IGSF23
PVR
BCL3
CBLC
APOE
TOMM40
RELB
GEMIN7
PPP1R37
EXOC3L2
MARK4
KLC3
45
45.2
45.4
45.6
45.8
Position on chr19 (Mb)

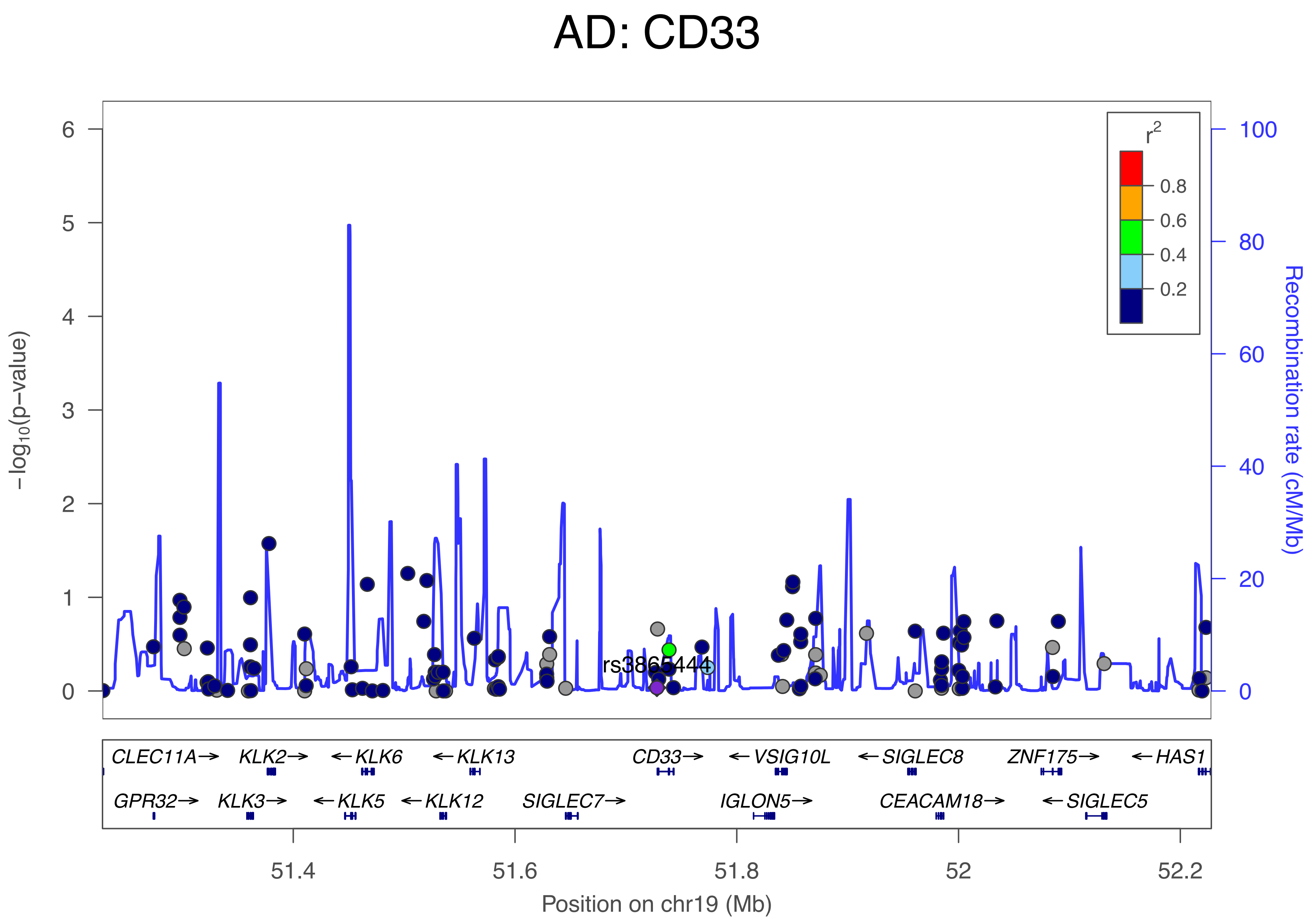

AD: CD33
−log10(p−value)
0
1
2
3
4
5
6
Recombination rate (cM/Mb)
0
20
40
60
80
100
r2
0.8
0.6
0.4
0.2
rs3865444
CLEC11A→
KLK2→
←KLK6
←KLK13
CD33→
←VSIG10L
←SIGLEC8
ZNF175→
←HAS1
GPR32→
KLK3→
←KLK5
←KLK12
SIGLEC7→
IGLON5→
CEACAM18→
←SIGLEC5
51.4
51.6
51.8
52
52.2
Position on chr19 (Mb)

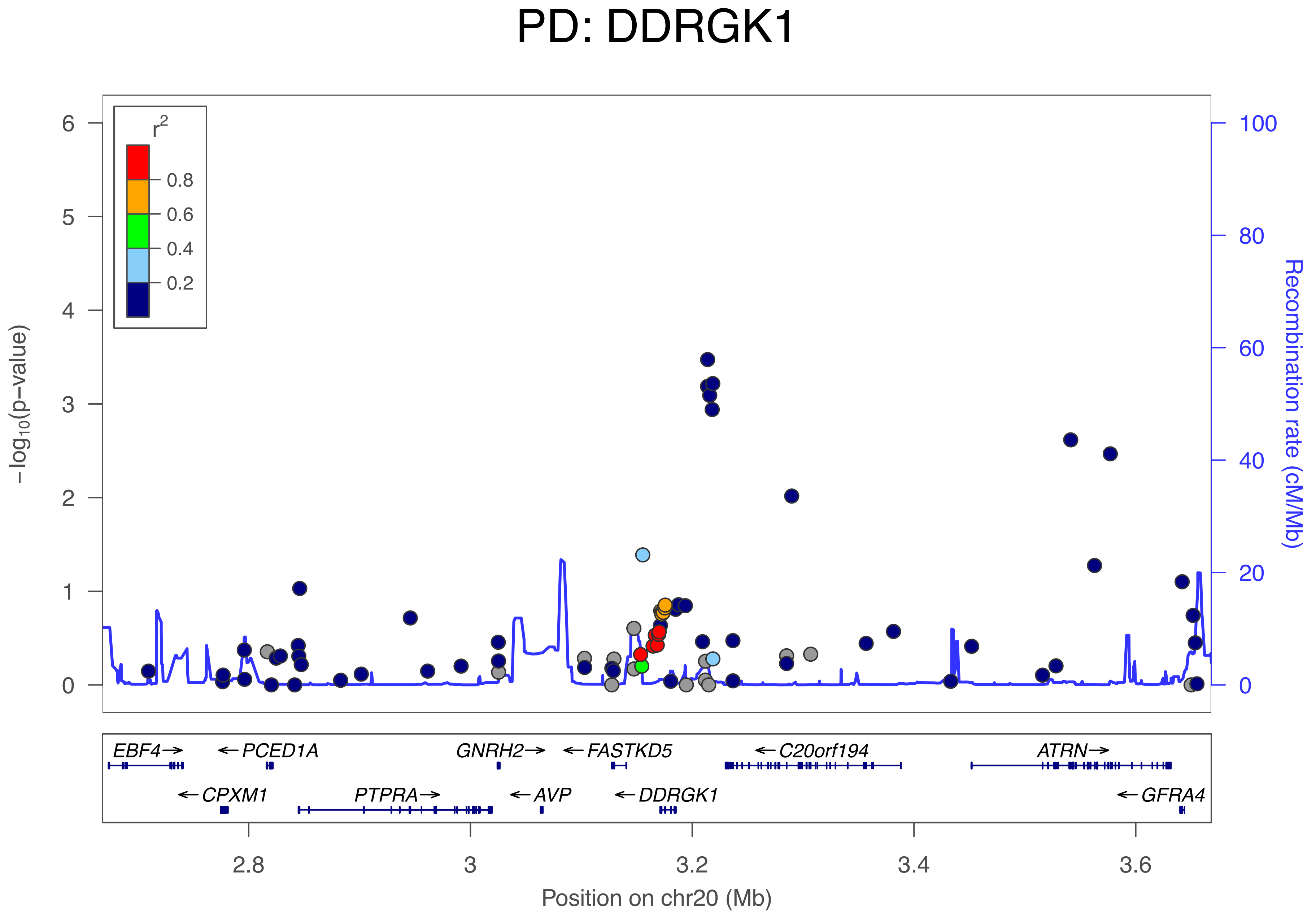
PD: DDRGK1
r2
0.8
0.6
0.4
0.2
6
5
4
3
2
1
0
−log10(p−value)
100
80
60
40
20
0
Recombination rate (cM/Mb)
EBF4→
←PCED1A
GNRH2→
←FASTKD5
←C20orf194
ATRN→
←CPXM1
PTPRA→
←AVP
←DDRGK1
←GFRA4
2.8
3
3.2
3.4
3.6
Position on chr20 (Mb)

AD: CASS4

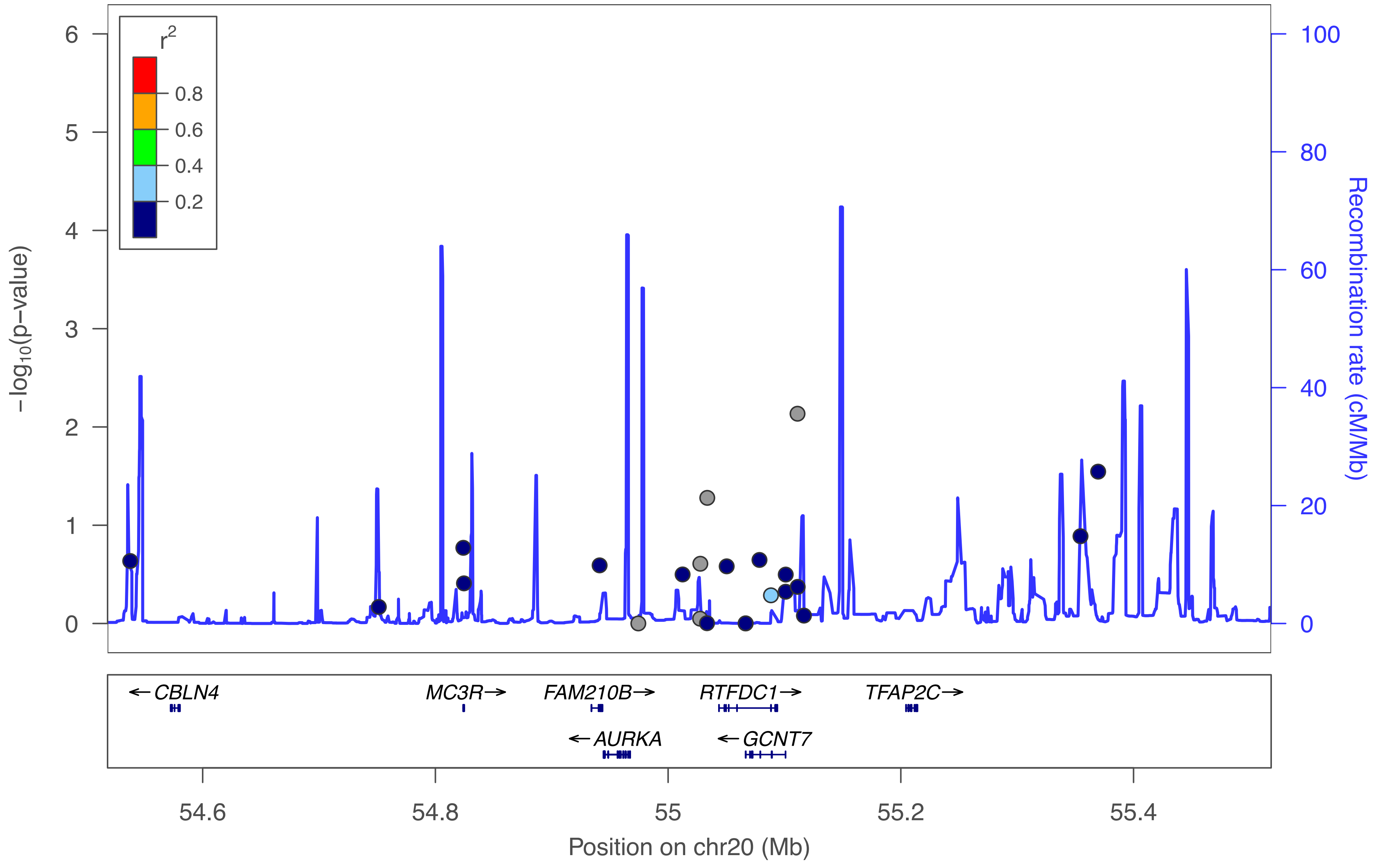

r2
0.8
0.6
0.4
0.2
-log10(p-value)
Recombination rate (cM/Mb)
6
5
4
3
2
1
0
100
80
60
40
20
0
CBLN4
MC3R
FAM210B
RTFDC1
TFAP2C
AURKA
GCNT7
54.6
54.8
55
55.2
55.4
Position on chr20 (Mb)

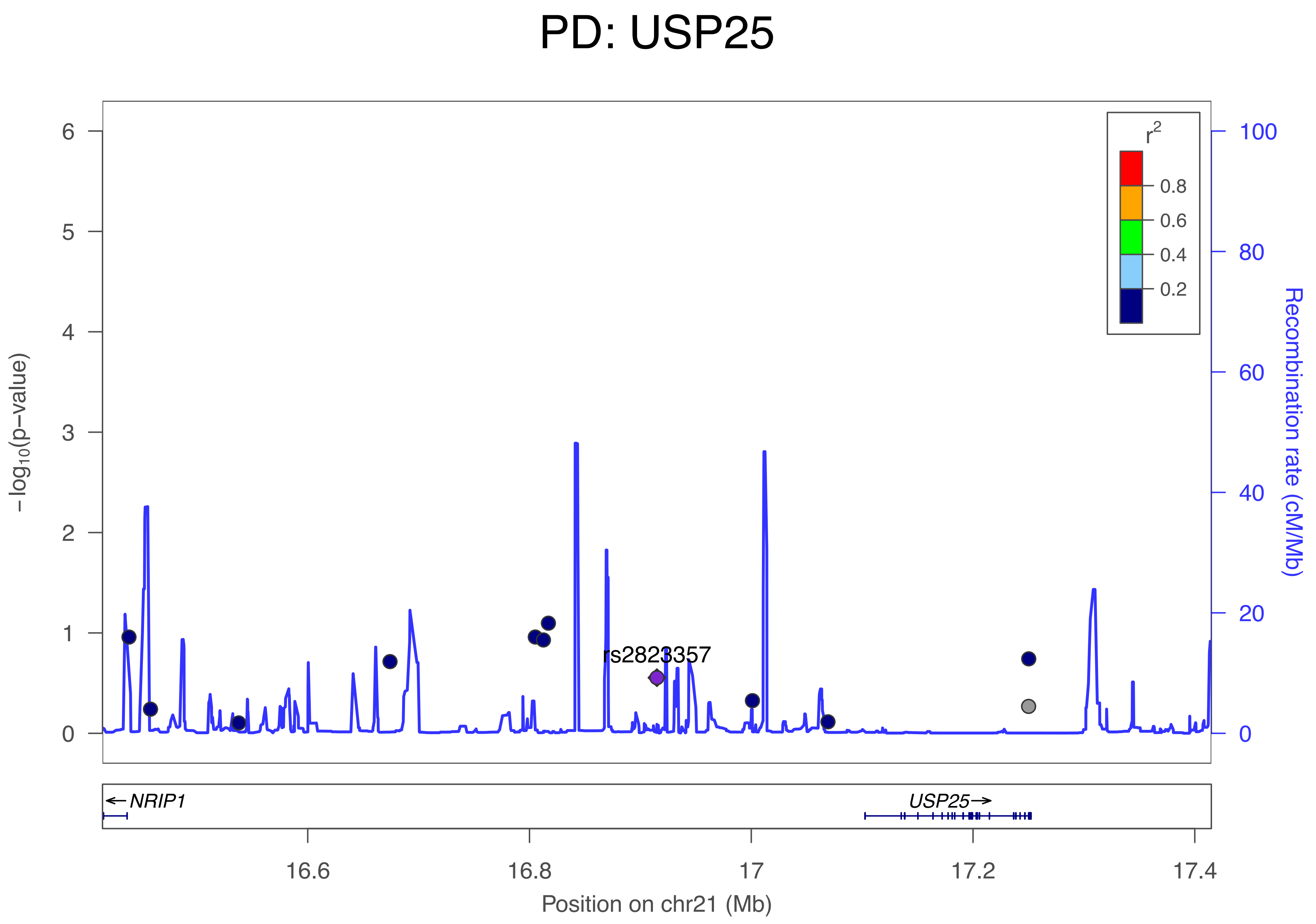

PD: USP25
r²
0.8
0.6
0.4
0.2
rs2823357
-log10(p-value)
Recombination rate (cM/Mb)
NRIP1
USP25
Position on chr21 (Mb)
