# Supplementary material for: Genetic analysis implicates APOE, SNCA and suggests lysosomal dysfunction in the etiology of dementia with Lewy bodies
Source: Hum Mol Genet. 2014 Jun 27;23(23):6139–46. doi: 10.1093/hmg/ddu334 (PMC4222357; doi:10.1093/hmg/ddu334)
Supplement: Supplementary Data [file supp_ddu334_ddu334supp_table1.docx]

Supplementary Table 1: Coverage of the previously associated genomic regions with PD or AD in this study.

| Study/Named Region | Chr | Start | End | Reported top hit | N Markers NeuroX | Best R2 on NeuroX |
| --- | --- | --- | --- | --- | --- | --- |
| PD_MegaMeta:GBA/SYT11 | 1 | 154635036 | 155635036 | rs35749011 | 90 | 0.903 |
| PD_MegaMeta:RAB7L1/NUCKS1 | 1 | 205223572 | 206223572 | rs823118 | 106 | 1 |
| AD_IGAP2013:CR1 | 1 | 207192049 | 208192049 | rs6656401 | 55 | 0.406 |
| PD_MegaMeta:SIPA1L2 | 1 | 232164611 | 233164611 | rs10797576 | 47 | 1 |
| AD_IGAP2013:BIN1 | 2 | 127392810 | 128392810 | rs6733839 | 29 | 0.326 |
| PD_MegaMeta:ACMSD/TMEM163 | 2 | 135039967 | 136039967 | rs6430538 | 260 | 1 |
| PD_MegaMeta:STK39 | 2 | 168610394 | 169610394 | rs1474055 | 48 | 0.925 |
| AD_IGAP2013:INPP5D | 2 | 233568476 | 234568476 | rs35349669 | 42 | 0.906 |
| PD_MegaMeta:KRT8P25/APOOP2 | 3 | 87020857 | 88020857 | rs115185635 | 9 | 1 |
| PD_MegaMeta:NMD3 | 3 | 160492864 | 161492864 | rs34016896 | 77 | 1 |
| PD_MegaMeta:MCCC1 | 3 | 182262437 | 183262437 | rs12637471 | 114 | 1 |
| PD_MegaMeta:TMEM175/GAK/DGKQ | 4 | 451947 | 1451947 | rs34311866 | 189 | 1 |
| PD_MegaMeta:BST1 | 4 | 15237101 | 16237101 | rs11724635 | 91 | 1 |
| PD_MegaMeta:FAM47E/SCARB2 | 4 | 76698986 | 77698986 | rs6812193 | 212 | 1 |
| PD_MegaMeta:SNCA | 4 | 90126111 | 91126111 | rs356182 | 226 | 1 |
| AD_IGAP2013:MEF2C | 5 | 87723420 | 88723420 | rs190982 | 6 | 0.07 |
| AD_IGAP2013:HLA-DRB5–HLA-DRB1 | 6 | 32078530 | 33078530 | rs9271192 | 1226 | 1 |
| PD_MegaMeta:HLA-DQB1 | 6 | 32166660 | 33166660 | rs9275326 | 1208 | 1 |
| AD_IGAP2013:CD2AP | 6 | 46987762 | 47987762 | rs10948363 | 31 | 0.076 |
| PD_MegaMeta:GPNMB | 7 | 22793746 | 23793746 | rs199347 | 52 | 1 |
| AD_IGAP2013:NME8 | 7 | 37341534 | 38341534 | rs2718058 | 21 | 0.238 |
| AD_IGAP2013:ZCWPW1 | 7 | 99504446 | 100504446 | rs1476679 | 115 | 0.375 |
| AD_IGAP2013:EPHA1 | 7 | 142610762 | 143610762 | rs11771145 | 49 | 1 |
| PD_MegaMeta:FGF20 | 8 | 16197091 | 17197091 | rs591323 | 40 | 1 |
| AD_IGAP2013:PTK2B | 8 | 26695121 | 27695121 | rs28834970 | 52 | 0.161 |
| AD_IGAP2013:CLU | 8 | 26967686 | 27967686 | rs9331896 | 65 | 1 |
| PD_MegaMeta:MMP16 | 8 | 88873041 | 89873041 | rs60298754 | 8 | 0.123 |
| PD_MegaMeta:ITGA8 | 10 | 15061543 | 16061543 | rs7077361 | 96 | 1 |
| PD_MegaMeta:INPP5F | 10 | 121036327 | 122036327 | rs117896735 | 69 | 0.844 |
| AD_IGAP2013:CELF1 | 11 | 47057871 | 48057871 | rs10838725 | 75 | 0.405 |
| AD_IGAP2013:MS4A6A | 11 | 59423508 | 60423508 | rs983392 | 55 | 0.978 |
| PD_MegaMeta:DLG2 | 11 | 83044472 | 84044472 | rs3793947 | 101 | 1 |
| AD_IGAP2013:PICALM | 11 | 85367875 | 86367875 | rs10792832 | 48 | 0.977 |
| AD_IGAP2013:SORL1 | 11 | 120935587 | 121935587 | rs11218343 | 21 | 0.03 |
| PD_MegaMeta:MIR4697 | 11 | 133265367 | 134265367 | rs329648 | 78 | 1 |
| PD_MegaMeta:LRRK2 | 12 | 40114434 | 41114434 | rs76904798 | 368 | 1 |
| PD_MegaMeta:CCDC62 | 12 | 122803586 | 123803586 | rs11060180 | 85 | 1 |
| AD_IGAP2013:FERMT2 | 14 | 52900629 | 53900629 | rs17125944 | 19 | 0.686 |
| PD_MegaMeta:GCH1 | 14 | 54848869 | 55848869 | rs11158026 | 125 | 1 |
| PD_MegaMeta:TMEM229B | 14 | 67484370 | 68484370 | rs1555399 | 57 | 1 |
| AD_IGAP2013:SLC24A4-RIN3 | 14 | 92426952 | 93426952 | rs10498633 | 59 | 0.091 |
| PD_MegaMeta:VPS13C | 15 | 61494134 | 62494134 | rs2414739 | 65 | 1 |
| PD_MegaMeta:BCKDK/STX1B | 16 | 30621793 | 31621793 | rs14235 | 210 | 1 |
| PD_MegaMeta:SREBF/RAI1 | 17 | 17215101 | 18215101 | rs11868035 | 114 | 1 |
| PD_MegaMeta:MAPT | 17 | 43494648 | 44494648 | rs17649553 | 411 | 1 |
| AD_IGAP2013:DSG2 | 18 | 28588958 | 29588958 | rs8093731 | 80 | 0.164 |
| PD_MegaMeta:RIT2 | 18 | 40173380 | 41173380 | rs12456492 | 51 | 1 |
| AD_IGAP2013:ABCA7 | 19 | 563443 | 1563443 | rs4147929 | 107 | 0.974 |
| PD_MegaMeta:SPPL2B | 19 | 1863319 | 2863319 | rs62120679 | 90 | 0.718 |
| AD_IGAP2013:APOE | 19 | 44409039 | 47412650 | NA | 247 | NA |
| AD_IGAP2013:CD33 | 19 | 51227962 | 52227962 | rs3865444 | 107 | 1 |
| PD_MegaMeta:DDRGK1 | 20 | 2668166 | 3668166 | rs8118008 | 78 | 0.928 |
| AD_IGAP2013:CASS4 | 20 | 54518260 | 55518260 | rs7274581 | 21 | 0.127 |
| PD_MegaMeta:USP25 | 21 | 16414905 | 17414905 | rs2823357 | 13 | 1 |

Study/Named Region: GWAS study that identified the specific locus and mane attributed to the locus; Chr: Chromosome; N Markers NeuroX: number of markers present in the NeuroX chip; Best R2 on NeuroX: best LD value for the markers present on the NeuroX chip.

Supplementary Table 2: Previously associated genomic regions with PD or AD and their results in our cohort of pathologically proven DLB samples.

| Study/Named Region | Reported top hit | Reported p-value | Reported OR | Best DLB hit | P-value | OR [95% CI] |
| --- | --- | --- | --- | --- | --- | --- |
| PD_MegaMeta:GBA/SYT11 | rs35749011 | 1.37x-29 | 1.824 | exm105846 | 0.003459 | 1.605 [1.169-2.203] |
| PD_MegaMeta:RAB7L1/NUCKS1 | rs823118 | 1.66x-16 | 1.122 | exm142459 | 0.01229 | 0.7013 [0.5312-0.9258] |
| AD_IGAP2013:CR1 | rs6656401 | 5.7x-24 | 1.18 | exm-rs17045328_ver2 | 0.01615 | 1.584 [1.089-2.303] |
| PD_MegaMeta:SIPA1L2 | rs10797576 | 4.87x-10 | 1.131 | exm159162 | 0.01471 | 0.28 [0.1007-0.7786] |
| AD_IGAP2013:BIN1 | rs6733839 | 6.9x-44 | 1.22 | exm225387 | 0.02029 | 1.154 [1.023-1.302] |
| PD_MegaMeta:ACMSD/TMEM163 | rs6430538 | 9.13x-20 | 0.875 | NeuroX_rs77922444 | 0.001016 | 0.6746 [0.5334-0.8531] |
| PD_MegaMeta:STK39 | rs1474055 | 1.15x-20 | 1.214 | NeuroX_rs5007396 | 0.06687 | 0.89 [0.7857-1.008] |
| AD_IGAP2013:INPP5D | rs35349669 | 3.2x-8 | 1.08 | exm276137 | 0.03814 | 1.187 [1.009-1.397] |
| PD_MegaMeta:KRT8P25/APOOP2 | rs115185635 | 0.02241 | 1.142 | NeuroX_rs62267706 | 0.1772 | 0.7898 [0.5606-1.113] |
| PD_MegaMeta:NMD3 | rs34016896 | 1.08x-05 | 1.067 | NeuroX_rs11922959 | 0.0273 | 1.161 [1.017-1.325] |
| PD_MegaMeta:MCCC1 | rs12637471 | 2.14x-21 | 0.842 | exm367051 | 0.07848 | 0.7497 [0.5439-1.033] |
| PD_MegaMeta:TMEM175/GAK/DGKQ | rs34311866 | 1.02x-43 | 0.786 | exm380436 | 0.03211 | 0.8325 [0.704-0.9845] |
| PD_MegaMeta:BST1 | rs11724635 | 9.44x-18 | 1.126 | exm390468 | 0.01538 | 2.941 [1.229-7.037] |
| PD_MegaMeta:FAM47E/SCARB2 | rs6812193 | 2.95x-11 | 0.907 | NeuroX_rs6825004 | 0.0004744 | 0.7825 [0.682-0.8979] |
| PD_MegaMeta:SNCA | rs356182 | 4.16x-73 | 0.76 | NeuroX_rs894280 | 0.000004184 | 0.7492 [0.6625-0.8472] |
| AD_IGAP2013:MEF2C | rs190982 | 3.2x-8 | 0.93 | exm-rs17421627 | 0.09348 | 0.8123 [0.6371-1.036] |
| AD_IGAP2013:HLA-DRB5–HLA-DRB1 | rs9271192 | 2.9x-12 | 1.11 | NeuroX_rs34531599 | 0.000002298 | 1.773 [1.398-2.248] |
| PD_MegaMeta:HLA-DQB1 | rs9275326 | 1.19x-12 | 0.826 | NeuroX_rs34531599 | 0.000002298 | 1.773 [1.398-2.248] |
| AD_IGAP2013:CD2AP | rs10948363 | 5.2x-11 | 1.1 | exm553859 | 0.02289 | 0.4003 [0.182-0.8808] |
| PD_MegaMeta:GPNMB | rs199347 | 1.18x-12 | 1.11 | NeuroX_rs10256996 | 0.01296 | 0.8559 [0.757-0.9677] |
| AD_IGAP2013:NME8 | rs2718058 | 4.8x-9 | 0.93 | NeuroX_rs16879765 | 0.07396 | 1.192 [0.9832-1.444] |
| AD_IGAP2013:ZCWPW1 | rs1476679 | 5.6x-10 | 0.91 | exm642810 | 0.0007947 | 2.293 [1.412-3.723] |
| AD_IGAP2013:EPHA1 | rs11771145 | 1.1x-13 | 0.9 | exm665677 | 0.01846 | 6.399 [1.366-29.97] |
| PD_MegaMeta:FGF20 | rs591323 | 6.68x-08 | 0.916 | NeuroX_rs34008571 | 0.00006474 | 1.284 [1.136-1.451] |
| AD_IGAP2013:PTK2B | rs28834970 | 7.4x-14 | 1.1 | exm692181 | 0.01112 | 0.7167 [0.5542-0.9269] |
| AD_IGAP2013:CLU | rs9331896 | 2.8x-25 | 0.86 | exm692181 | 0.01112 | 0.7167 [0.5542-0.9269] |
| PD_MegaMeta:MMP16 | rs60298754 | 0.181 | 1.078 | exm-rs7004633 | 0.0314 | 0.8353 [0.709-0.9841] |
| PD_MegaMeta:ITGA8 | rs7077361 | 4.16x-05 | 1.092 | exm811504 | 0.04 | 0.7945 [0.6378-0.9895] |
| PD_MegaMeta:INPP5F | rs117896735 | 4.34x-13 | 1.624 | exm860137 | 0.004374 | 2.442 [1.321-4.512] |
| AD_IGAP2013:CELF1 | rs10838725 | 1.1x-8 | 1.08 | exm904319 | 0.05348 | 0.3116 [0.09541-1.018] |
| AD_IGAP2013:MS4A6A | rs983392 | 6.1x-16 | 0.9 | exm914525 | 0.01405 | 0.697 [0.5225-0.9297] |
| PD_MegaMeta:DLG2 | rs3793947 | 3.96x-07 | 0.929 | NeuroX_rs1469610 | 0.01376 | 1.165 [1.032-1.315] |
| AD_IGAP2013:PICALM | rs10792832 | 9.3x-26 | 0.87 | exm946210 | 0.03952 | 2.992 [1.054-8.492] |
| AD_IGAP2013:SORL1 | rs11218343 | 9.7x-15 | 0.77 | exm963990 | 0.1437 | 0.8955 [0.7724-1.038] |
| PD_MegaMeta:MIR4697 | rs329648 | 9.83x-12 | 1.105 | exm971731 | 0.0301 | 0.7234 [0.5398-0.9693] |
| PD_MegaMeta:LRRK2 | rs76904798 | 5.24x-14 | 1.155 | NeuroX_rs11175645 | 0.0346 | 0.808 [0.663-0.9847] |
| PD_MegaMeta:CCDC62 | rs11060180 | 6.02x-12 | 1.105 | exm1047614 | 0.06279 | 1.416 [0.9816-2.041] |
| AD_IGAP2013:FERMT2 | rs17125944 | 7.9x-9 | 1.14 | exm1101999 | 0.1191 | 0.7065 [0.4564-1.094] |
| PD_MegaMeta:GCH1 | rs11158026 | 5.85x-11 | 0.904 | NeuroX_rs10140164 | 0.003227 | 0.8288 [0.7314-0.9391] |
| PD_MegaMeta:TMEM229B | rs1555399 | 6.63x-14 | 0.897 | exm1110045 | 0.03622 | 1.149 [1.009-1.308] |
| AD_IGAP2013:SLC24A4-RIN3 | rs10498633 | 5.5x-9 | 0.91 | exm1122623 | 0.04507 | 1.147 [1.003-1.311] |
| PD_MegaMeta:VPS13C | rs2414739 | 1.23x-11 | 1.113 | exm1166899 | 0.006645 | 1.347 [1.086-1.671] |
| PD_MegaMeta:BCKDK/STX1B | rs14235 | 2.43x-12 | 1.103 | NeuroX_rs72799316 | 0.001476 | 0.8136 [0.7164-0.9239] |
| PD_MegaMeta:SREBF/RAI1 | rs11868035 | 5.98x-05 | 0.939 | exm1300132 | 0.002889 | 1.308 [1.096-1.561] |
| PD_MegaMeta:MAPT | rs17649553 | 2.37x-48 | 0.769 | NeuroX_rs17660132 | 0.001158 | 0.7761 [0.6661-0.9043] |
| AD_IGAP2013:DSG2 | rs8093731 | 1.0x-4 | 0.73 | exm2253388 | 0.03106 | 1.144 [1.012-1.292] |
| PD_MegaMeta:RIT2 | rs12456492 | 7.74x-12 | 0.904 | NeuroX_rs6507515 | 0.001946 | 1.357 [1.119-1.646] |
| AD_IGAP2013:ABCA7 | rs4147929 | 1.1x-15 | 1.15 | exm1397738 | 0.0004783 | 2.614 [1.524-4.482] |
| PD_MegaMeta:SPPL2B | rs62120679 | 5.57x-07 | 1.097 | exm1403990 | 0.02339 | 2.696 [1.144-6.354] |
| AD_IGAP2013:APOE | NA | NA | NA | exm-rs769449 | 7.086E-35 | 2.711 [2.313-3.177] |
| AD_IGAP2013:CD33 | rs3865444 | 3.0x-6 | 0.94 | exm1495693 | 0.02666 | 3.495 [1.156-10.57] |
| PD_MegaMeta:DDRGK1 | rs8118008 | 3.04x-11 | 1.111 | NeuroX_dbSNP_rs117725082 | 0.0003362 | 0.5672 [0.4161-0.7733] |
| AD_IGAP2013:CASS4 | rs7274581 | 2.5x-8 | 0.88 | exm1551966 | 0.00733 | 4.509 [1.5-13.56] |
| PD_MegaMeta:USP25 | rs2823357 | 0.02675 | 1.031 | exm-rs1297265 | 0.07995 | 0.8949 [0.7903-1.013] |

Study/Named Region: GWAS study that identified the specific locus and mane attributed to the locus. Reported top hit, p-value and OR refer to the original publication values. Best DLB hit: most significant variant in the DLB cohort.

Supplementary Table 3: Top significantly associated variants when comparing pathologically confirmed DLB cases with controls.

| CHR | BP | SNP | A1 | OR | P | Reporting Study |
| --- | --- | --- | --- | --- | --- | --- |
| 19 | 45410002 | exm-rs769449 | A | 2.711 | 7.09E-35 | AD_IGAP2013:APOE |
| 19 | 45395909 | NeuroX_rs34404554 | G | 2.328 | 1.06E-27 | AD_IGAP2013:APOE |
| 19 | 45395619 | exm-rs2075650 | G | 2.314 | 2.07E-27 | AD_IGAP2013:APOE |
| 19 | 45396665 | NeuroX_dbSNP_rs59007384_rep | T | 2.123 | 8.55E-27 | AD_IGAP2013:APOE |
| 19 | 45396219 | NeuroX_dbSNP_rs157582_rep | A | 2.105 | 3.56E-26 | AD_IGAP2013:APOE |
| 19 | 45395714 | NeuroX_rs157581 | G | 2.081 | 1.52E-25 | AD_IGAP2013:APOE |
| 19 | 45394336 | NeuroX_rs71352238 | C | 2.222 | 6.87E-25 | AD_IGAP2013:APOE |
| 19 | 45394969 | NeuroX_rs184017 | C | 2.03 | 8.88E-24 | AD_IGAP2013:APOE |
| 19 | 45388130 | NeuroX_rs34342646 | A | 2.149 | 2.88E-23 | AD_IGAP2013:APOE |
| 19 | 45395844 | NeuroX_rs34095326 | A | 2.028 | 3.14E-17 | AD_IGAP2013:APOE |
| 19 | 45414451 | exm-rs439401 | T | 0.6253 | 2.23E-11 | AD_IGAP2013:APOE |
| 19 | 45395266 | exm-rs157580 | G | 0.6417 | 4.99E-11 | AD_IGAP2013:APOE |
| 4 | 90760883 | NeuroX_rs894280 | C | 0.7492 | 4.18E-06 | PD_MegaMeta:SNCA |
| 4 | 90764699 | NeuroX_rs7687945 | C | 0.7507 | 4.87E-06 | PD_MegaMeta:SNCA |
| 19 | 45403412 | NeuroX_rs1160985 | T | 0.7542 | 8.72E-06 | AD_IGAP2013:APOE |
| 19 | 45405062 | NeuroX_rs1038026 | G | 0.7561 | 1.03E-05 | AD_IGAP2013:APOE |
| 4 | 90757272 | NeuroX_rs3756059 | G | 0.7587 | 1.05E-05 | PD_MegaMeta:SNCA |
| 19 | 45407788 | NeuroX_rs7259620 | A | 0.7574 | 1.22E-05 | AD_IGAP2013:APOE |
| 19 | 45412079 | exm1479366 | T | 0.5444 | 1.64E-05 | AD_IGAP2013:APOE |

Chr: Chromosome; BP position according to hg19; SNP: marker name in the NeuroX array; A1: minor allele in the whole dataset; OR: odds ratio; P: association p-value.
